# Global, regional, and national trends and burden of multiple sclerosis in adolescents and young adults: a data analysis from 1990 to 2021 and projections to 2040

**DOI:** 10.3389/fimmu.2025.1685316

**Published:** 2025-10-22

**Authors:** Peixi Zhao, Chang Guan, Jing Lu, Ying Zhang, Baitong Wang, Lin Huang, Ding Ye, Hongmei Nan, Jian Wang, Peng Xu

**Affiliations:** ^1^ College of Traditional Chinese Medicine, Changchun University of Chinese Medicine, Changchun, Jilin, China; ^2^ Research Center of Traditional Chinese Medicine, The Affiliated Hospital of Changchun University of Chinese Medicine, Changchun, Jilin, China; ^3^ Center for Brain Disorders, The Affiliated Hospital of Changchun University of Chinese Medicine, Changchun, Jilin, China; ^4^ Zhejiang Chinese Medical University School of Public Health, Hangzhou, Zhejiang, China; ^5^ Zhejiang Chinese Medical University School of Basic Medical Sciences, Hangzhou, Zhejiang, China

**Keywords:** multiple sclerosis, epidemiology, global burden of disease, adolescents and young adults, disease projections

## Abstract

**Background and Objectives:**

Multiple sclerosis (MS) significantly impacts adolescents and young adults (aged 15–39 years), causing substantial neurological disability. Despite therapeutic advances, the global burden persists due to disparities in healthcare access and lifestyle factors. This study analyzes global, regional, and national trends and burdens of MS using Global Burden of Disease (GBD) 2021 data, emphasizing the influence of the Socio-demographic Index (SDI).

**Methods:**

Using data from the GBD 2021 data, we conducted a secondary analysis. We assessed the prevalence, mortality, and disability-adjusted life years (DALYs) of MS across 204 countries/territories from 1990 to 2021, calculating age-standardized rates.

**Results:**

In 2021, global MS prevalence was 489,310 cases, associated with 1,424 deaths and 215,869 disability-adjusted life years (DALYs). The global age-standardized rates per 100,000 were: prevalence 22.2 (19.8 - 24.8), deaths 0.2 (0.2 - 0.2), and DALYs 11.4 (9.8 - 13.2). Compared to 1990, these rates decreased by 0.4%, 12.8%, and 11.0% respectively, while the number of prevalent cases increased by 52.4%. Sweden (161.6), Canada (134.2), and Norway (131.5) had the highest age-standardized prevalence. The UK (71.3) had the highest and Nauru (0.4) the lowest age-standardized DALY rate. Female MS death rates exceeded those of males across all ages, peaking at 20–24 years. Regionally, age-standardized DALY rates showed an inverse V-shaped relationship with the SDI.

**Discussion:**

Although the global MS burden among adolescents and young adults (AYAs) decreased, the substantial and rising prevalence demands attention. The burden from premature mortality and stark geographic variations in prevalence and DALYs indicate inadequate disease management, underscoring the need for enhanced awareness and effective interventions. Urgent, targeted healthcare policies are required to address geographical inequities, sex-specific pathophysiology, and modifiable risk factors such as smoking.

## Graphical Abstract

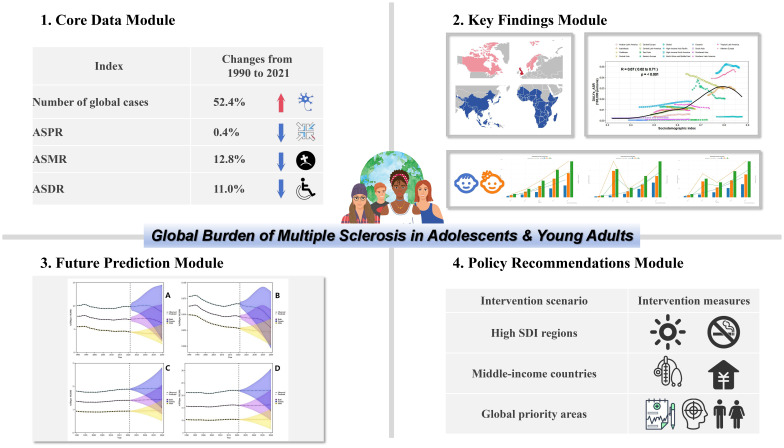

## Introduction

Multiple sclerosis (MS) is a chronic autoimmune disorder of the central nervous system characterized by inflammation, demyelination, and axonal damage. It is a leading cause of non-traumatic neurological disability in young adults, with a typical onset between 20 and 40 years of age and a marked female predominance (female-to-male ratio of approximately 3:1) (1–4). Notably, adolescents and young adults (AYAs, aged 15–39 years) represent a critically affected demographic. This life stage is pivotal for educational attainment, career establishment, and family planning, meaning that an MS diagnosis can incur substantial personal, societal, and economic burdens (5).

The epidemiological landscape of MS is complex and geographically heterogeneous, traditionally categorized into high-, medium-, and low-prevalence regions (6). While incidence rates are highest in Europe and North America, emerging evidence suggests changing patterns, possibly influenced by improved diagnostics and environmental factors (7). Understanding these trends, especially among AYAs, is crucial for effective healthcare planning and resource allocation.

Previous reports from the GBD study have quantified the MS burden globally. However, a comprehensive analysis specifically focused on the AYA population, characterized by unique socio-economic vulnerabilities and a high potential for therapeutic responsiveness, remains lacking. This gap is significant, as disease-modifying therapies (DMTs) can profoundly alter the disease course, underscoring the importance of early detection and intervention in this age group (3).

Therefore, leveraging data from the GBD 2021 study, we conducted a secondary analysis to evaluate the incidence, prevalence, mortality, and disability-adjusted life years (DALYs) attributable to MS from 1990 to 2021, with projections to 2040, exclusively among individuals aged 15–39 years. This study aims to provide a detailed assessment of the global, regional, and national burden of MS in AYAs, thereby informing targeted public health strategies and guiding future research efforts.

## Materials and methods

### Overview

The GBD 2021 study examined the effects of 369 diseases and injuries, together with 87 risk factors, between 1990 and 2021. This assessment covered 204 countries and territories, along with 21 regions. Building on the findings from GBD 2021 (8), we estimated the prevalence of MS in AYAs aged 15 to 39 years, disaggregated by sex, across the same 204 countries and territories from 1990 to 2021. Prevalence data was stratified into 5-year age brackets (15–19, 20–24, 25–29, 30–34, and 35–39 years) and by sex for all locations. This extensive age range (15–39 years) reflects a redefinition influenced by the interaction of biological evolutionary dynamics and socio-structural transformations (9). Consistent with previous iterations of the GBD, the definition of MS cases in GBD 2021 adhered to the McDonald criteria, while other established criteria, including the Poser criteria, Schumacher criteria, and Allison and Millar criteria, were also considered as references (10). The International Classification of Diseases (ICD) code for MS is 340 in the ninth version and G35 in the tenth revision.

### Case definition and data sources

MS predominantly affects young adults, leading to neurological disabilities that result in significant healthcare expenditures and diminished employment opportunities (11). The methodology adopted in the GBD 2021 study represents an advancement over previous iterations, with strict adherence to its standardized data curation protocols. Significantly, a systematic literature review was not conducted for the GBD 2021 data; the most recent systematic review was conducted for the GBD 2019 study by the Institute for Health Metrics and Evaluation. The search terms utilized in the previous systematic literature review, along with additional information, can be found in **Supplementary Table S1**. This study specifically examines the incidence of MS among AYAs, utilizing data from the GBD 2021. We acquired data from 204 countries and 21 regions, facilitating a thorough examination of the global and regional burden of MS. MS cases were identified using ICD codes G35 and 340. The GBD 2021 dataset supplied age-standardized incidence rates (ASIR), prevalence rates (ASPR), mortality rates (ASMR), and DALYs for MS. Data spanning the period from 1990 to 2021 were examined to evaluate worldwide and regional patterns, with particular attention to variations by age, sex, and the SDI.

The SDI encompasses metrics pertaining to income, education, and fertility. This research specifically focused on AYAs, incorporating data from 204 countries and regions. The prevalence, mortality, and DALYs associated with MS were obtained from the GBD 2021 dataset. MS cases were accurately identified using the ICD-9 code 340 and ICD-10 code G35. The SDI served as a key instrument for assessing the socio-demographic development status of each nation.

### Age standardization

To guarantee consistency in assessing the burden of the MS burden across populations with diverse age structures, we utilized age standardization using the direct method. Age-standardized rates (ASR) for incidence, prevalence, mortality, and DALYs were estimated using the GBD standard population as a reference. This methodological strategy tackles variations in age distribution, enabling significant comparisons across various regions and time periods. The ASR is derived by calculating a weighted mean of age-specific rates, with each rate being modified based on the population size of the corresponding age group in the standard population. This approach offers a projection of the rates that would be expected if all populations had an identical age distribution, thus enhancing precision cross-country and cross-regional comparisons of the MS burden.

### Compilation of results

To estimate the years of life lost (YLL) in accordance with the GBD standard life table, the mortality count within each age group was multiplied by the remaining life expectancy for that specific group. These YLL values were then combined with the years lived with disability (YLD) to compute the DALYs. The degree of uncertainty was evaluated by performing 1000 simulations at each computational step, taking into account variations from various origins such as input data, corrections for measurement inaccuracies, and assessments of remaining non-sampling error. The uncertainty intervals were determined by the 25th and 975th percentiles of the ordered draws. The association between the burden of multiple sclerosis, expressed in DALYs, and the SDI across 21 regions and 204 countries and territories was analyzed using Smoothing Splines models (12). The SDI serves as a composite metric that ranges from 0, representing the lowest level of development, to 1, indicating the highest level of development. This index incorporates multiple factors, such as per capita income adjusted for delays, the average per capita GDP over the past ten years, the mean years of schooling for individuals aged 15 and above, and the total fertility rate among women younger than 25. Furthermore, age-standardized point prevalence, mortality rates, and DALY rates were visual using R software (version 4.4.2).

### Bayesian age-period-cohort prediction

To project the future burden of MS among AYAs, we employed Bayesian Age-Period-Cohort (BAPC) models to forecast the incidence, prevalence, mortality, and disability-adjusted life years (DALY) rates from 2021 to 2040 at both global and national levels, leveraging data from 204 countries and territories spanning 1990 to 2021. The BAPC framework was implemented using the Integrated Nested Laplace Approximations (INLA) within the R statistical computing environment, which efficiently estimates marginal posterior distributions and circumvents the convergence and mixing challenges often associated with traditional Markov Chain Monte Carlo (MCMC) methods for Bayesian inference (13–15). The model’s predictive performance was rigorously evaluated through an out-of-sample validation procedure. Specifically, the model was fitted using data from 1990 to 2010, and forecasts were generated for the period 2011–2021. The predicted values were then compared against the observed data from the GBD 2021 study to assess accuracy. To guard against overfitting, we incorporated penalized complexity priors within the INLA framework, which regularize model parameters by penalizing unnecessary complexity. This approach has been shown to yield superior predictive performance for age-standardized rates compared to alternative models such as Joinpoint and Poisson regression (16, 17). The projections derived from this robust modeling strategy will provide critical insights for informing long-term public health planning and the development of targeted prevention and control strategies for MS in the AYA population.

## Results

### Global trends

The 2021 GBD report indicated that globally, 489,300 cases of MS were diagnosed within the AYAs. The age-standardized point prevalence stood at 22·2 per 100,000, signifying a 0·4% decline since 1990. In the same year, MS accounted for 1,423·9 deaths within this age group, with an age-standardized mortality rate was 18·9 per 1,000, representing a substantial 12·8% reduce since 1990. Moreover, in 2021, the DALYs caused by MS among AYAs aged 15 to 39 years amounted to 215,900. The age-standardized rate was 11·4 DALYs per 100,000, demonstrating an 11·0% reduction since 1990 (**Table 1**).

**Table 1:** Prevalent cases, deaths, and DALYs for MS disease diagnosed among AYAs in 2021, and percentage change in age standardized rates (ASRs), by GBD region, from 1990 to 2021 (generated from data available at https://ghdx.healthdata.org/gbd-results-tool).

	Prevalence (95% UI)			Deaths (95% UI)			DALYs (95% UI)		
	No, in thousands (95% UI)	ASRs per 100 000 (95% UI)	Percentage change in ASRs from 1990 to 2021	Number (95% UI)	ASRs per 100 (95%UI)	Percentage change in ASRs from 1990 to 2021	No, in thousands (95% UI)	ASRs per 100 000 (95% UI)	Percentage change in ASRs from 1990 to 2021
Global	489·3 (406·4, 583·5)	22·2 (19·8, 24·8)	-0·4 (-3·6, 3·3)	1423·9 (1252·7, 1606·8)	1890·8 (1776·1, 1978·6)	-12·8 (-17·5, -8·3)	215·9 (173·5, 268·5)	11·4 (9·8, 13·2)	-11 (-14, -8)
High-income Asia Pacific	4 (3, 5·2)	9·2 (7·6, 11·2)	6·9 (3·4, 10·3)	6·8 (6·3, 7·6)	310·5 (287·4, 331)	-11·1 (-17, -5·2)	1·5 (1·1, 2)	3·7 (2·8, 4·7)	-0·3 (-3·8, 2·7)
High-income North America	115·2 (103·5, 126·8)	103·6 (96·4, 111·3)	6·9 (-1·7, 16·3)	161·4 (154·6, 168·1)	8133·1 (7632·3, 8568·1)	40·4 (31·7, 49·2)	38·9 (30, 48·3)	49·2 (41·8, 56·5)	11·8 (5·6, 18·5)
Western Europe	107·2 (87·7, 126·6)	91·4 (80·9, 103)	34·1 (29·5, 40)	200·5 (189·9, 211·4)	7272·4 (6773, 7662·4)	10·5 (3·7, 17·1)	39·3 (30·4, 49·3)	45·3 (38·2, 52·1)	15·9 (10·5, 20·4)
Australasia	5·2 (4·1, 6·4)	59·9 (52, 68·8)	48·7 (36·3, 61·9)	10·6 (9·5, 11·9)	4482·6 (3925·4, 5043·5)	5·5 (-10·9, 21·6)	2 (1·4, 2·6)	28·8 (23·7, 34·4)	22·2 (9·5, 36·6)
Andean Latin America	1·6 (1·1, 2·2)	9·2 (7·5, 11)	46·8 (40·5, 54·3)	7·3 (5·1, 10·3)	1080·1 (815·6, 1397·9)	84 (38·7, 141·9)	0·9 (0·7, 1·1)	5·9 (4·7, 7·3)	62·1 (38·3, 89·5)
Tropical Latin America	10 (7·3, 13·3)	21·2 (18·1, 25)	24·3 (19·1, 30·6)	33·1 (31·2, 35·3)	1319·8 (1204·7, 1410·6)	20·9 (11·1, 31·2)	4·7 (3·7, 6·1)	9·5 (7·8, 11·5)	23·7 (17·3, 31·4)
Central Latin America	7·7 (5·7, 9·9)	10·4 (8·5, 12·2)	56·9 (47·8, 66·5)	94·5 (84·1, 105·9)	2154·8 (1917·6, 2432·3)	110·9 (84·7, 140·1)	7·7 (6·6, 8·9)	10·5 (9·2, 11·9)	91·2 (71·8, 111·6)
Southern Latin America	5·1 (3·9, 6·3)	23 (19, 27·4)	1·5 (-3·2, 6·1)	13·8 (12·1, 15·6)	1591·4 (1427·9, 1745·1)	-40·4 (-47·2, -33·7)	2·2 (1·6, 2·8)	11·4 (9·4, 13·7)	-24·8 (-32, -17·8)
Caribbean	1·7 (1·3, 2·1)	11·7 (9·7, 13·8)	25·1 (18·8, 31·3)	15·3 (12·4, 19·8)	2118·7 (1835·8, 2445·8)	21·9 (5·1, 40·7)	1·4 (1·1, 1·7)	10·7 (9·3, 12·6)	17·9 (5·5, 31)
Central Europe	16·7 (14·2, 19·4)	42·8 (38·6, 47·3)	11·4 (7, 16·9)	65·4 (57·5, 74·4)	5539 (5032·8, 6114·4)	-38·1 (-45, -31·2)	8·2 (6·6, 9·9)	29·9 (26·3, 33·7)	-31·1 (-36·7, -25·5)
Eastern Europe	21·5 (18·8, 24·3)	27·2 (25, 29·9)	15·8 (6·6, 28·1)	142·3 (123·1, 163·5)	3391·1 (3018, 3798)	-37·9 (-45·3, -29·7)	13·8 (11·7, 15·8)	20·4 (17·7, 22·8)	-28·8 (-36·1, -21·8)
Central Asia	4·2 (3·1, 5·6)	29·1 (25·8, 32·7)	-0·8 (-4·9, 4)	12·2 (9·3, 14·7)	1480·5 (1125·2, 1764·7)	-35·6 (-50·9, -20·1)	1·9 (1·4, 2·5)	10·8 (8·4, 13·7)	-18·3 (-27·9, -9·8)
North Africa and Middle East	91·2 (73·9, 111)	45·1 (39, 52)	30·6 (27, 34·5)	198·9 (161·4, 239)	1883·8 (1539·5, 2212·9)	80 (24·6, 214·4)	35·7 (27·8, 45·3)	17·9 (14·5, 21·7)	41·5 (24, 63·3)
South Asia	56·6 (42·6, 73·4)	8·4 (6·9, 10·1)	18·9 (14·4, 23·4)	55·8 (34·5, 75·3)	182·6 (119, 241·7)	85·6 (24·9, 286·3)	19·2 (13·4, 26·6)	3 (2·2, 3·9)	28·4 (18·5, 39·2)
Southeast Asia	6·9 (4·9, 9·8)	2·4 (1·9, 3)	10 (6·9, 13·2)	25·2 (20·8, 29·8)	126·8 (106·8, 146·3)	76·4 (30·9, 173·4)	3·5 (2·7, 4·5)	1·2 (0·9, 1·4)	30·8 (16·5, 49·4)
East Asia	12·7 (9, 17·9)	2·3 (1·8, 2·9)	45·2 (38·7, 52·6)	17·2 (13·1, 22·2)	59·7 (47·1, 74·2)	13·3 (-30·6, 92)	4·6 (3·2, 6·4)	0·9 (0·6, 1·1)	35·7 (14·2, 55·7)
Oceania	0·1 (0·1, 0·1)	1·6 (1·2, 2·1)	0·6 (-2·6, 4)	0 (0, 0)	0·5 (0·1, 0·8)	25·6 (-11·9, 89·1)	0 (0, 0)	0·5 (0·3, 0·7)	0·7 (-2·6, 4·3)
Western Sub-Saharan Africa	11·3 (8·5, 14·8)	8·3 (7, 9·9)	28·6 (23·8, 35·3)	343·1 (209·3, 498·5)	606 (370, 880·1)	71·5 (5·6, 203·7)	26·4 (17·2, 37·2)	6·4 (4·7, 8·5)	52·5 (13·4, 109)
Eastern Sub-Saharan Africa	6·6 (4·8, 8·8)	4·8 (3·8, 5·9)	5·6 (2·9, 8·7)	11·6 (4·5, 18·1)	202 (78·9, 306·1)	65·9 (18·2, 178·2)	2·6 (1·7, 3·6)	2 (1·4, 2·7)	19·8 (8·2, 35·7)
Central Sub-Saharan Africa	1·9 (1·3, 2·6)	4·3 (3·5, 5·4)	8·3 (3·8, 13)	3·3 (1·5, 5·5)	216·4 (103·2, 332·7)	62·2 (4·5, 159·6)	0·7 (0·5, 1)	1·9 (1·4, 2·6)	23·3 (5·2, 43·3)
Southern Sub-Saharan Africa	2·1 (1·5, 2·7)	7·3 (6, 8·8)	6·4 (2·7, 10·5)	5·5 (4·4, 6·7)	1017·5 (798·8, 1238·4)	29·7 (4, 75·7)	0·9 (0·7, 1·1)	5·3 (4·3, 6·4)	14·9 (-0·4, 34·1)

95% UI = 95% uncertainty intervals.

### Regional level

In 2021, regions with higher income levels, such as North America (103·6), Western Europe (91·4), and Australasia (59·9) exhibited the highest age-standardized point prevalences of MS per 100,000 individuals. Conversely, the lowest rates were reported in Oceania (1·6), East Asia (2·3), and Southeast Asia (2·4) (**Table 1**). Similarly, high-income regions such as North America (8133·1), Western Europe (7272·4), and Central Europe (5539·0) exhibited the highest age-standardized death rates from MS per 100,000 individuals in 2021, whereas the lowest rates were recorded in Oceania (0·5), East Asia (59·7), and Southeast Asia (126·8) (**Table 1**). **Supplementary Figures S1**-**S3** illustrate the age-standardized point prevalence, mortality, and DALY rates of MS by sex across all regions included in the GBD study for the year 2021.

From 1990 to 2021, the largest rises in age-standardized point prevalence of MS were observed in Central Latin America (56·9%), Australasia (48·7%), and Andean Latin America (46·8%), whereas Central Asia exhibited the most significant decrease (−0·8%) (**Table 1**). Concurrently, the period witnessed the most substantial increases in age-standardized death rates due to MS in Central Latin America (110·9%), South Asia (85·6%), and Andean Latin America (84·0%). In contrast, the most notable reductions in mortality rates were recorded in Southern Latin America (−40·4%), Central Europe (−38·1%), and Eastern Europe (−37·9%) (**Table 1**). Additionally, the most substantial rises in age-standardized DALY rates of MS during this timeframe were identified in Central Latin America (91·2%), Andean Latin America (62·1%), and Western Sub-Saharan Africa (52·5%), while Eastern Europe (−28·8%), Central Europe (−31·1%), and Southern Latin America (−24·8%) experienced the most significant decreases (**Table 1**). **Supplementary Figures S4**-**S6** depict the percentage changes in age-standardized point prevalence, mortality, and DALY rates for MS by gender between 1990 and 2021.

Between 1990 to 2021, the global prevalence of MS cases increased by 34·5%, rising from 160,530 to 215,869 cases. In 1990, the highest numbers of prevalent cases were recorded in high-income regions such as North America, North Africa and the Middle East, and Western Europe, and these same regions continued to report the highest numbers in 2021 (**Supplementary Table S2**). Moreover, the number of deaths attributed to MS rose from 1,276 in 1990 to 1,424 in 2021, with Western Sub-Saharan Africa, Western Europe, and North Africa and the Middle East reporting the highest mortality figures in 2021 (**Supplementary Table S3**). Furthermore, the number of DALYs due to MS increased from 160,530 in 1990 to 215,869 in 2021, with Western Europe, high-income North America, and North Africa and the Middle East exhibiting the highest DALY counts in 2021 (**Supplementary Table S4**).

### National level

**Figure 1:**
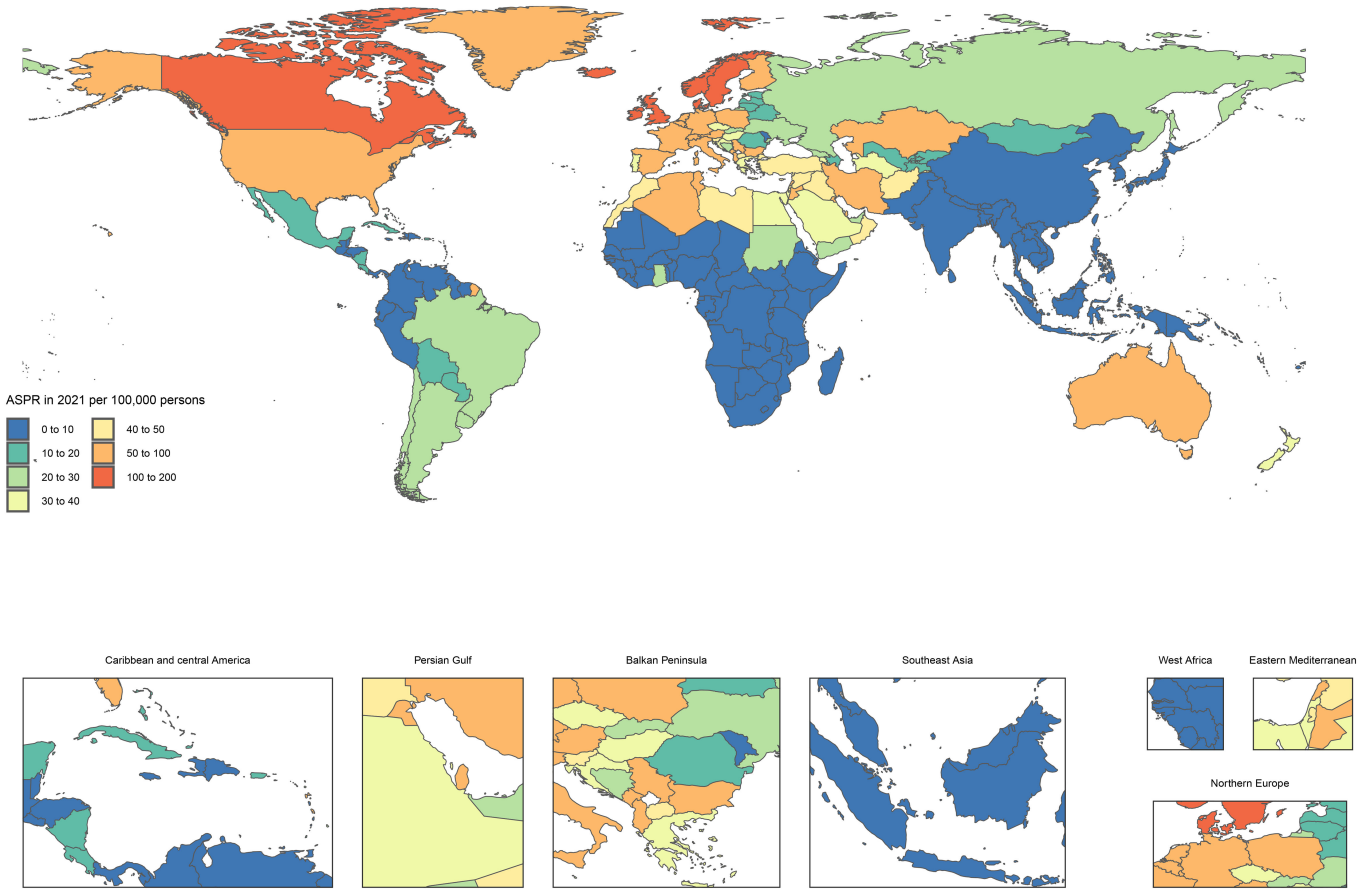
Age standardized point prevalence of MS diagnosed among AYAs per 100,000 population in 2021, by country (generated from data available at https://ghdx.healthdata.org/gbd-results-tool).

In 2021, there was considerable variation in the national age-standardized point prevalence of MS across different countries. The rates varied between 1.5 and 161.6 cases per 100,000 individuals. The highest prevalences were recorded in Sweden (161·6), Canada (134·2), and Norway (131·5), whereas the lowest estimates were reported in Papua New Guinea (1·5), Nauru (1·6), Tuvalu (1·7), and Kiribati (1·7) (**Figure 1**, **Supplementary Table S2**).

**Figure 2:**
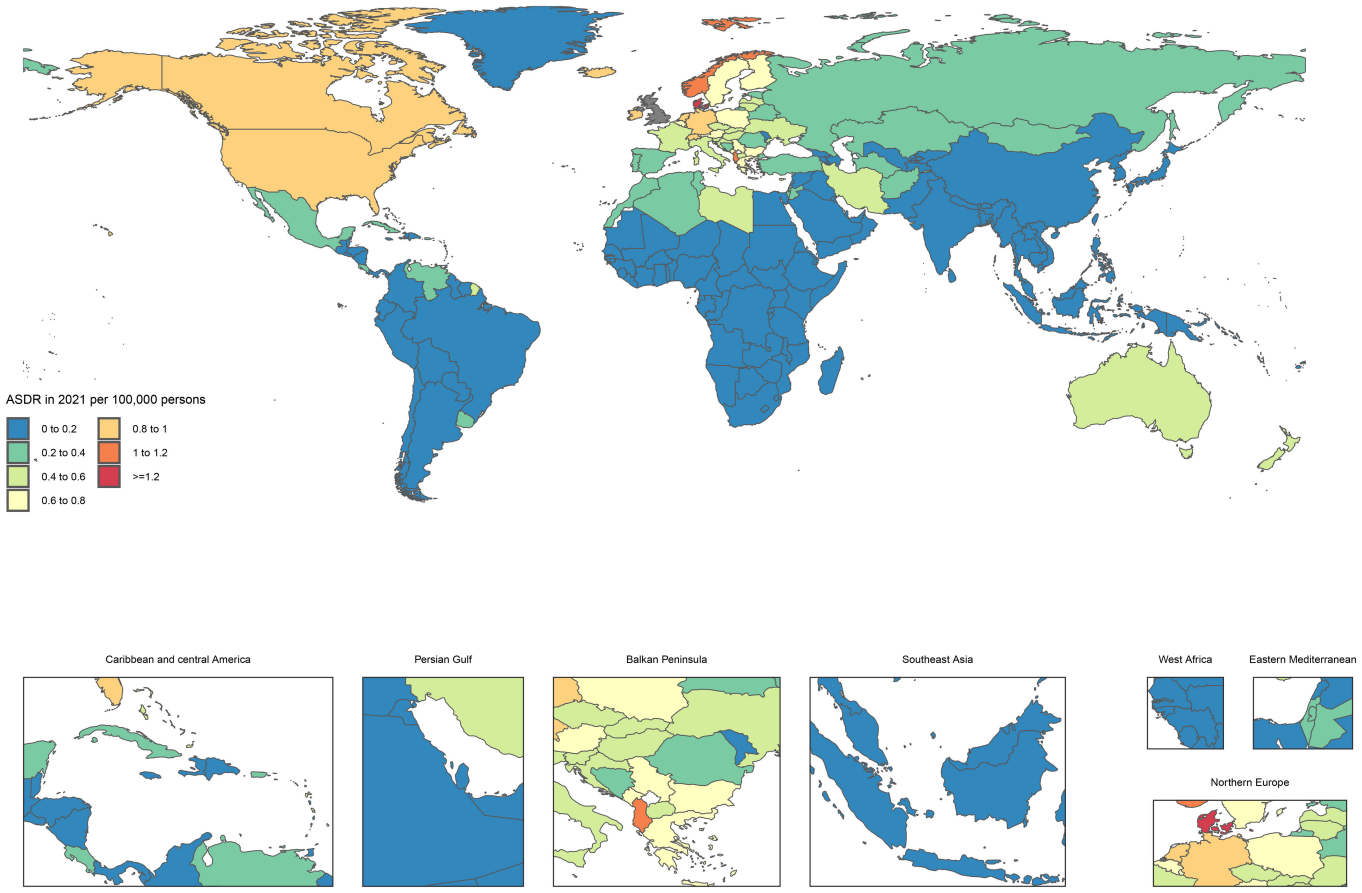
Age standardized death rate of MS diagnosed among AYAs per 100,000 population in 2021, by country (generated from data available at https://ghdx.healthdata.org/gbd-results-tool).

MS is typically characterized as a chronic disease with a slow progression, allowing patients to survive for many years or even decades following diagnosis. Consequently, despite the potential for severe neurological impairments, the mortality rate directly attributable to MS remains relatively low in comparison to other diseases. In 2021, the global age-standardized death rate due to MS was less than 1 per 100,000 individuals in most countries (**Figure 2**, **Supplementary Table S3**).

In 2021, the national age-standardized DALY rate for MS varied significantly, ranging from 0·2 to 71·3 per 100,000 individuals. The highest DALY rates were reported in the United Kingdom (71·3), Denmark (65·8), and Norway (62·9), while the minimum rates were noted in Nauru (0·4) and Papua New Guinea (0·4) (**Supplementary Figure S7**, **Supplementary Table S4**).

The age-standardized point prevalence showed considerable variation in percentage change across various countries between 1990 and 2021. Taiwan (Province of China) exhibited the largest increase at 149·9%, followed by Egypt at 115·8% and Ghana at 80·1%. Conversely, the most notable decreases were observed in Hungary (−20·5%), Uzbekistan (−20%), and New Zealand (−9%) (**Supplementary Table S2**). During the same period, Mauritius (27425·1%), Kuwait (24716·6%), and Bahrain (996·3%) experienced the highest increases in age-standardized death rates; however, these figures should be cautiously interpreted because of their low baseline values. The most significant reductions in death rates were recorded in Estonia (−63·4%), Slovenia (−53·1%), and Latvia (−50·3%) (**Supplementary Table S3**). Furthermore, Mauritius (303·1%), Libya (147·4%), and Taiwan (Province of China) (137·1%) showed the most substantial rises in age-standardized DALY for MS from 1990 to 2021. In contrast, the most considerable reductions during this period occurred in Estonia (−59·3%), Latvia (−47·2%), and Slovenia (−43·6%) (**Supplementary Table S4**).

### Age and sex patterns

**Figure 3:**
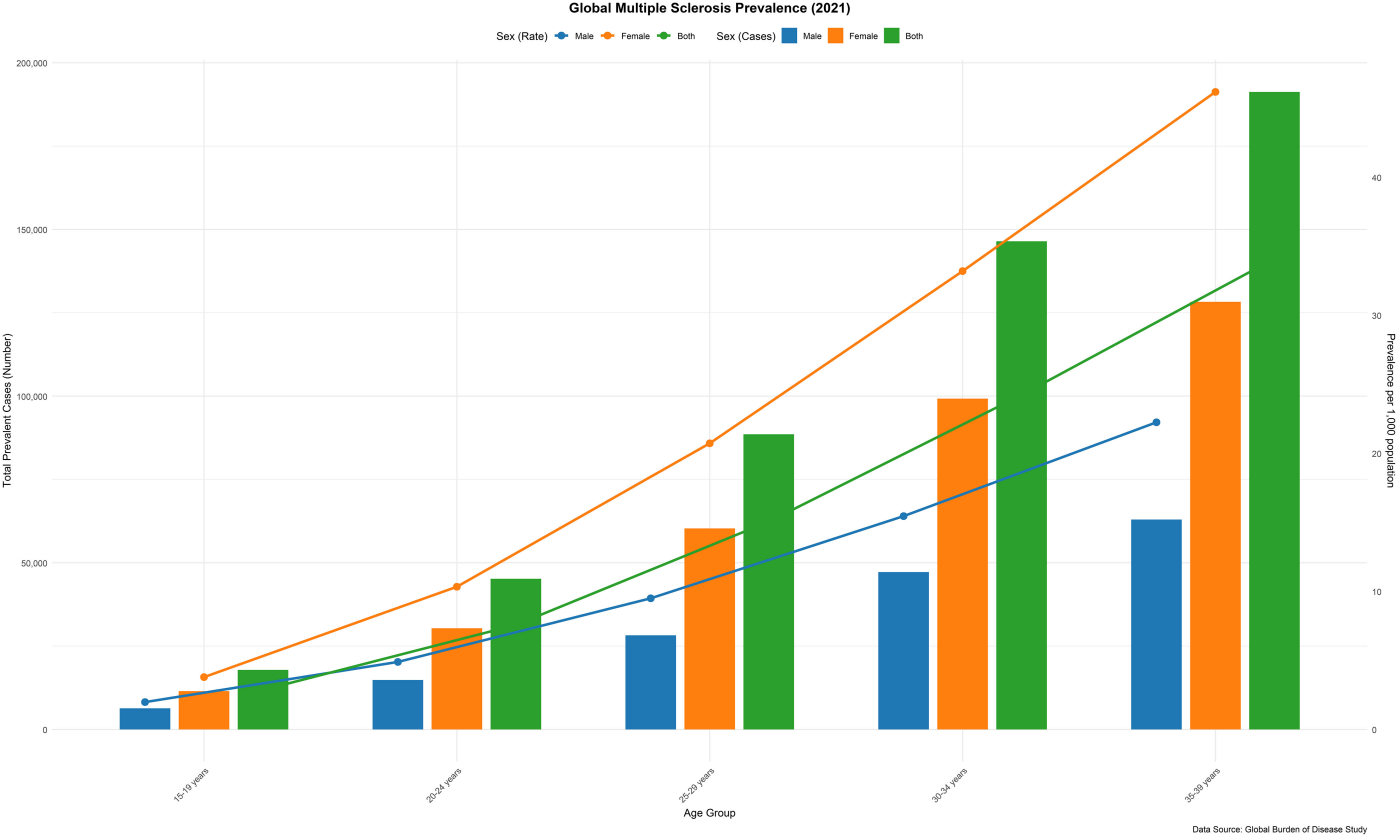
Number of prevalent cases globally and prevalence of MS per 100–000 population, by age and sex in 2021. (generated from data available at https://ghdx.healthdata.org/gbd-results-tool).

In 2021, the global point prevalence of MS in AYAs continued to increase. Similarly, the prevalence of cases rose as age increased. The prevalence of MS was significantly higher in females compared to males (**Figure 3**). In the same year, the global death rate from MS was elevated across all age groups in females, reaching its highest in the 20–24 age group. The highest number of deaths occurred in the 35–39 age group for both sexes (**Supplementary Figure S8**). Among females, the global DALY rate for MS increased until the 20–24 age group, subsequently declining before rising again. In contrast, the DALY rate for males consistently increased with age, yet remained lower than that of females across all age groups. Additionally, the number of DALYs peaked in the 35–39 age group among AYAs (**Supplementary Figure S9**). Females accounted for 68% of global DALYs, with peak mortality occurring in the 20–24 age group.

### Association with the SDI

**Figure 4:**
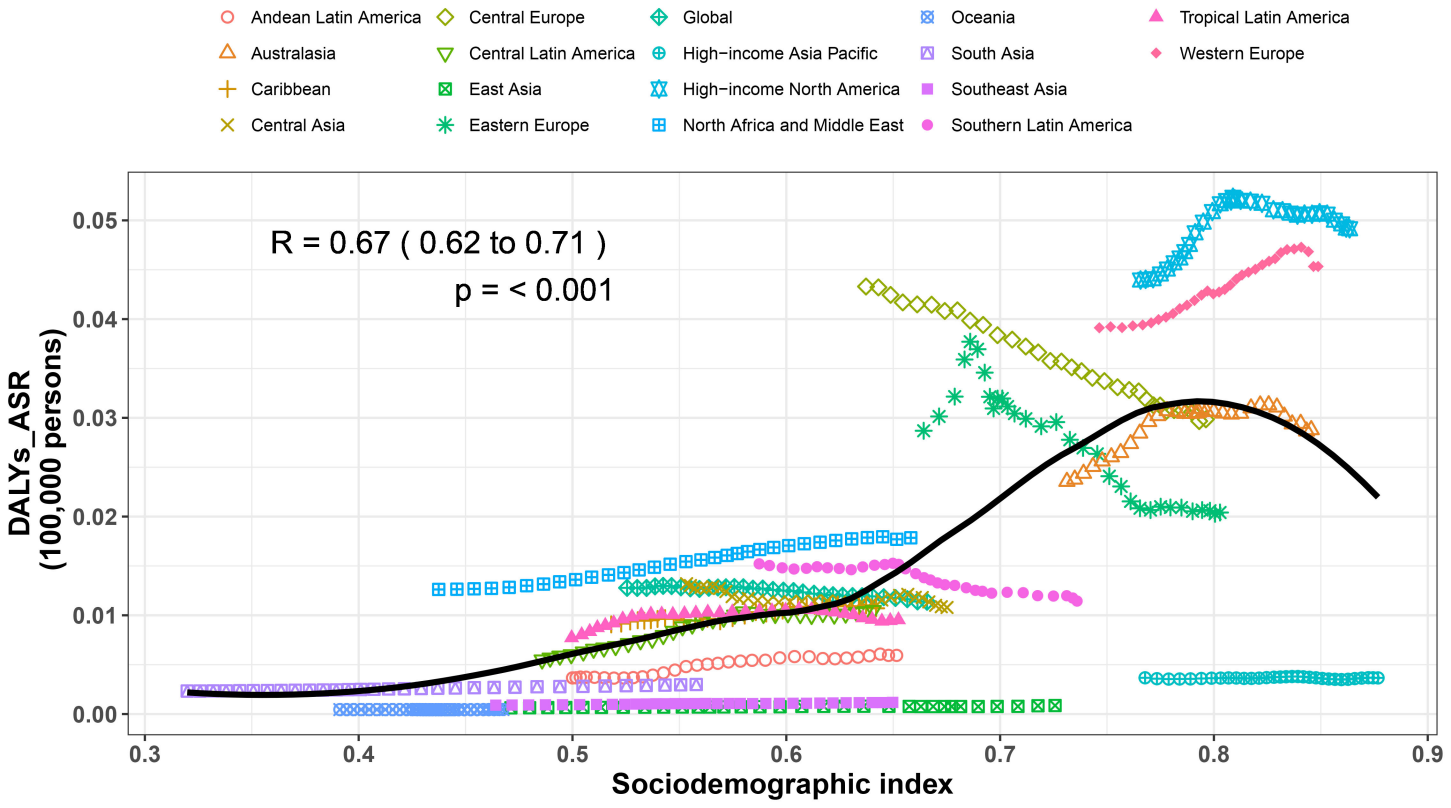
Age standardized disability adjusted life year (DALY) rates of MS for the 21 GBD regions by sociodemographic index, 1990–2021. Thirty points are plotted for each region and show the observed age standardized DALY rates from 1990 to 2021 for that region. Expected values, based on sociodemographic index and disease rates in all locations, are shown as a solid line. Regions above the solid line represent a higher than expected burden (eg, High−income North America) and regions below the line show a lower than expected burden (eg, High−income Asia Pacific) (generated from data available at https://ghdx.healthdata.org/gbd-results-tool).

At the regional level, our analysis demonstrated a reversed V-shaped relationship between the SDI and the age-adjusted DALY rate for MS from 1990 to 2021. The age-standardized DALY rates exhibited an exponential increase with rising SDI values, reaching a peak at approximately 0·8, followed by a subsequent declining. During this period, regions such as Western Europe, high-income North America, and North Africa, as well as the Middle East experienced DALY rates that surpassed what was anticipated given their SDI levels during the same period. In contrast, areas including Oceania, high-income Asia Pacific, Andean Latin America, Southeast Asia, and East Asia witnessed a lower-than-anticipated burden between 1990 and 2021 (**Figure 4**). Specific countries and territories, including Afghanistan, Albania, Denmark, Palestine, Morocco, and Serbia, demonstrated significantly higher burdens than expected, while Singapore, Japan, Guam, Monaco, and San Marino exhibited considerably lower burdens than anticipated (**Supplementary Figure S10**).

### Risk factors

Globally, smoking has been identified as the leading contributor to DALYs associated with MS. The percentage of DALYs related to smoking varies significantly across different GBD regions. Notably, among males, the share of DALYs resulting from MS that is linked to smoking is greater (**Supplementary Figures S11**, **S12**).

### Bayesian age-period-cohort prediction model

**Figure 5:**
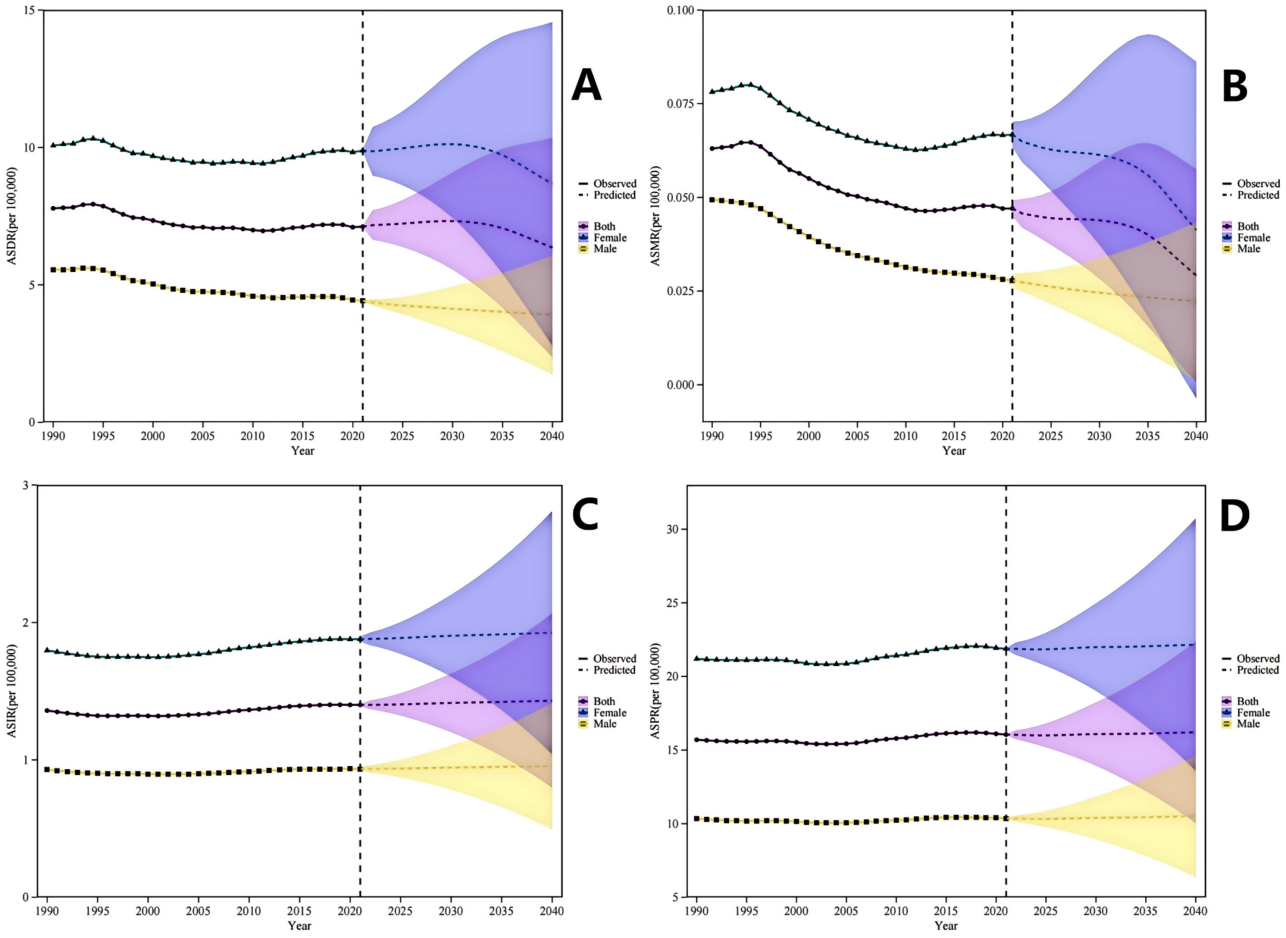
Observed and Projected Sex-Specific Trends in Multiple Sclerosis Burden Among Adolescents and Young Adults, 1990-2040. **(A)** Age-Standardized Disability-Adjusted Life Years Rate (ASDR) demonstrates a declining trajectory for both sexes combined, with males exhibiting a steeper projected decline relative to females during the 2022–2040 period. **(B)** Age-Standardized Mortality Rate (ASMR) depicts a decreasing trend from 1990 to 2040, with the decline being more pronounced among males compared to females. **(C)** Age-Standardized Incidence Rate (ASIR) trends are presented for both sexes combined, males, and females, encompassing observed data (1990-2021) and projected estimates (2022-2040). **(D)** Age-Standardized Prevalence Rate (ASPR) displays a stable pattern for both sexes combined, males, and females throughout the observation and projection interval. ASIR, Age-Standardized Incidence Rate; ASPR, Age-Standardized Prevalence Rate; ASMR, Age-Standardized Mortality Rate; ASDR, Age-Standardized Disability-Adjusted Life Years Rate; DALY, Disability-Adjusted Life Years.

The four projections show the trends in observed and projected values of different indicators from 1990 to 2040, and are presented by sex (female, male, overall). Based on the prediction data, between 2021 and 2040, the ASDR and ASMR caused by MS are anticipated to decrease for both men and women, with a more rapid decline observed in men compared to women. Nevertheless, during this same timeframe, the ASIR and ASPR of MS for all genders are expected to stabilize (**Figure 5**).

## Discussion

### Principal findings

This research provides an in-depth examination of the prevalence, mortality rates, and DALYs related to MS between 1990 and 2021, utilizing data from the GBD 2021 study. The examination covers ASRs in 204 countries and territories. Notably, the global burden of MS in AYAs, has exhibited a declining tendency. This decline is mainly manifested through reductions in the ASRs for incidence, prevalence, mortality, and DALYs. These developments emphasize major progress in global healthcare systems, the handling of chronic illnesses, and the treatment of MS. However, despite this progress, the rising prevalence of MS, alongside declining ASRs, suggests s improvements in diagnostic capabilities and survival rates, yet also underscores unmet needs in progressive MS therapeutics—particularly regarding disability driven by PIRA, which remains inadequately addressed by conventional disease-modifying therapies (DMTs).

In 2021, MS accounted for 489,300 prevalent cases, 1,424 deaths, and 215,900 DALYs. Although there has been a decrease in age-standardized point prevalence, mortality, and DALY rates associated with MS over the past thirty years, the absolute numbers have risen. This increase can likely be explained by factors including population growth, an aging demographic, and longer life expectancy. The inverted V-shaped relationship between the SDI and DALYs suggests that early industrialization (SDI 0·4-0·8) increases MS risk through lifestyle changes (e.g., smoking, vitamin D deficiency), while advanced development (SDI >0·8) reduces the burden via improved healthcare access. This nonlinear relationship reflects the dual effect of economic transition.

This study provides a comprehensively analysis of the burden of MS in AYAs, highlighting the notably high incidence among young females, the substantial burden in high-income regions, and the influence of risk factors such as smoking. The results strongly support the improvement of medical resource distribution, strengthening health education, and advancing the prevention and management of chronic diseases.

### Comparison to other studies

Epidemiological research reveals that the prevalence of MS exhibits a distinct pattern marked by a latitude gradient, a higher incidence in females, and a temporal increase over time. Furthermore, these research findings indicate that the interplay between environmental and genetic factors plays a critical role in the onset of the disease. The aging population affected by MS, coupled with its considerable economic impact, necessitates a reassessment of public health strategies. Notably, there is a clear stratification in economic burden, with annual social costs increasing sharply as disability levels rise (18, 19).

In contrast to the studies previously mentioned, our research specifically focuses on the global youth demographic, aged 15 to 39. Upon reviewing the GBD database for MS-related studies, two studies focusing on China were identified. All MS indicators in China are below the global average. Despite these relatively low indicator values, the absolute number of patients is significant due to China’s large population base. The burden of MS in China is characterized by low prevalence rates, a high absolute number of cases, and premature mortality (20, 21).

Furthermore, a comprehensive global epidemiological analysis of MS across all age groups reveals that the burden is higher in northern regions, more prevalent among females, and rapidly increasing in developed areas. Latitude and healthcare expenditure are identified as significant predictors of MS prevalence (22).

### Age and sex differences in MS

Consistently, gender variations in MS show a greater prevalence and severity in women than in men, with this disparity being especially significant among the AYA population. Traditionally, it is hypothesized that sex hormones significantly role in the pathogenesis of autoimmune diseases affecting the central nervous system (23). Early menarche has been identified as a risk factor for MS, and menstrual cycles may precipitate micro-relapses (24). Research indicates that the phenethylamine-dopamine D2 receptor-lysozyme signaling axis in the gut is a critical determinant of susceptibility to MS in female animals and young to middle-aged women, representing a novel extension of traditional perspectives (25).

For individuals in the AYA age group, fertility emerges as a significant concern. Although fertility is generally unaffected, sexual dysfunction is notably prevalent. Additionally, the use of assisted reproductive technologies may elevate the risk of relapse. The recurrence rate of MS typically decreases during pregnancy but experiences a significant rise in the postpartum period. Notably, pregnancy does not exacerbate MS progression, nor does MS elevate the risk of adverse pregnancy outcomes. The initial three months following childbirth constitute a high-risk period for relapses, making it essential to promptly resume DMT. However, due to the unique physiological conditions of pregnant women and the limited data regarding medication use during breastfeeding, adjustments to MS treatment must be made based on a physician’s assessment (26).

MS is particularly prevalent among young females, with associated comorbidities— including depression, anxiety, migraines, reproductive health issues, and urological and bowel problems—adversely affect patients’ quality of life. Implementing a multidisciplinary and holistic management approach may enhance treatment efficacy and enhance the standard of living for these individuals (24).

### Region

Data from the Valencian region suggest that individuals with MS often demonstrate a deficiency in vitamin D deficiency, with intake levels falling below 5 μg per day. This condition is exacerbated by high saturated fat consumption. To mitigate this issue, public health initiatives should prioritize the promotion of vitamin D-fortified foods and advocate for UV-safe outdoor activities (27).

Another study highlights a paradox: while the global prevalence of MS is roughly 33 cases per 100,000 individuals, official estimates within the Asia-Pacific region are conspicuously lower, ranging from 0 to 20 cases per 100,000. Among the 32 countries and regions in the Asia-Pacific, only six—namely China, Japan, and the Republic of Korea—have conducted relevant studies, collectively covering merely 8% of the entire region’s population (28).

Previously, it was a common belief that Caucasian populations exhibited the highest prevalence of MS. However, recent findings reveal a significant increase in MS prevalence among black individuals. The influence of geographical latitude remains considerable, with the prevalence rising by 11·7 cases per 100,000 for each additional latitude degree. Surprisingly, high prevalence rates have also been observed in mountainous regions, indicating possible new risk factors related to altitude or exposure to ultraviolet radiation (29).

Moving forward, it is imperative to mitigate the burden through hepatitis B virus (HBV) prevention, vitamin D supplementation, and optimizing healthcare resources. Particular attention should be given to rectifying diagnostic and treatment deficiencies that are prevalent in middle-income countries.

### Implications for policy and practice

Based on our findings—which reveal a persistent global burden, a complex inverse V-shaped relationship with socioeconomic development, significant female preponderance, and smoking as a leading risk factor—we propose the following targeted clinical and public health recommendations to mitigate the burden of MS in AYAs:

1. Tiered Strategies Based on Socioeconomic Development (SDI):

For High-SDI Regions (e.g., Western Europe, North America): While age-standardized rates are declining, the high absolute burden demands a strategic shift. Policymakers and healthcare systems should prioritize closing the gaps in managing progressive MS and disability accumulation independent of relapse activity (PIRA). This includes incentivizing research into neuroprotective and remyelinating therapies and integrating sensitive biomarkers (e.g., serum neurofilament light chain) into routine clinical practice for early detection of subclinical progression.

For Low- and Middle-SDI Regions (e.g., parts of Latin America, South Asia, Sub-Saharan Africa): The rising trends in ASRs observed in many of these regions call for urgent investment in neurological healthcare infrastructure. This involves training neurologists and primary care physicians to reduce diagnostic delays. Public health initiatives must prioritize cost-effective, primary prevention strategies tailored to young populations, such as nationwide smoking cessation campaigns and promoting vitamin D sufficiency through dietary fortification or safe sun exposure.

2. Implementing Sex-Specific and Life-Course Informed Care Pathways: The disproportionate burden among young women, peaking in the 20–24 age group, warrants the immediate development and dissemination of integrated care models. These models should seamlessly address family planning, pregnancy management (including the safe use of disease-modifying therapies before, during, and after pregnancy), and the management of prevalent comorbidities like depression and anxiety. Clear, evidence-based guidelines for healthcare providers on managing MS throughout a woman’s reproductive life are essential.

3. Strengthening Epidemiological Surveillance and Data Quality: The apparently low prevalence in regions like East and Southeast Asia may reflect significant under-diagnosis and surveillance gaps. Enhancing epidemiological capacity through the establishment of national MS registries and incorporating neurological assessments into broader national health surveys is critical. This will enable a more accurate quantification of the burden, ensuring that resources are allocated effectively and that regional specificities are understood.

4. Addressing Modifiable Risk Factors through Public Policy: Our study reinforces that smoking is a leading contributor to MS-related disability. This finding provides a strong mandate for public health authorities to intensify tobacco control measures specifically aimed at adolescents and young adults. Policies should include higher taxation, plain packaging, and digital media campaigns highlighting the link between smoking and MS risk/progression.

5. Fostering a Multidisciplinary and Patient-Centered Care Model: Given the multifaceted impact of MS on AYAs’ lives—affecting education, employment, and mental health—care should extend beyond neurology. Integrating services such as vocational rehabilitation, psychological support, and urology into MS care pathways is vital to improve overall quality of life and reduce the long-term socioeconomic impact of the disease.

## Limitations

This research provides an in-depth analysis of the global burden of MS among AYAs, utilizing data from GBD 2021. However, it is not without its limitations. Firstly, the data quality and accessibility in low-income countries are frequently less than ideal or completely absent, which may result in an underestimation of the true disease burden. Secondly, the study does not sufficiently investigate disparities between rural and urban populations, nor does it sufficiently consider the impact of socioeconomic factors and cultural influences on health behaviors (30). Furthermore, the reliance of GBD on ICD codes may result in an undercount of cases in regions that lack access to neurospecialists. Finally, while the Bayesian model provides valuable projections, its accuracy is contingent on the assumption that past trends will continue, which may be altered by unforeseen breakthroughs in treatment or prevention. Future studies should concentrate on enhancing data accuracy, improving the examination of internal disparities, and integrating more dynamic social and cultural elements.

## Conclusion

In conclusion, this study demonstrates that while the age-standardized burden of MS in AYAs is decreasing globally, the rising absolute number of cases demands sustained attention. The complex relationship with socioeconomic development, significant gender gap, and marked geographic heterogeneity revealed by our analysis call for differentiated public health responses. Future efforts must focus on improving data quality, elucidating the drivers of sex-based differences, and developing cost-effective interventions tailored to the specific needs of young adults with MS across diverse resource settings.

## Data Availability

The datasets presented in this study can be found in online repositories. The names of the repository/repositories and accession number(s) can be found below: https://ghdx.healthdata.org/gbd-results-tool.
