# Supplementary material for: Global, regional, and national trends and burden of multiple sclerosis in adolescents and young adults: a data analysis from 1990 to 2021 and projections to 2040
Source: Front Immunol. 2025 Oct 22;16:1685316. doi: 10.3389/fimmu.2025.1685316 (PMC12586062; doi:10.3389/fimmu.2025.1685316)
Supplement: Supplementary file 1 [file DataSheet1.zip › Table 3 (3).DOCX]

| **Table S3: Deaths due to MS in 1990 and 2021 and the percentage change in the age-standardised rates (ASRs) per 100,000, by location (Generated from data available from [http://ghdx·healthdata·org/gbd-results-tool](http://ghdx.healthdata.org/gbd-results-tool))** | | | | | |
| --- | --- | --- | --- | --- | --- |
|  | **1990** | | **2021** | | Percentage change in the ASRs per 100000 |
|  | No (95%UI) | ASRs per 100000 (95%UI) | No (95%UI) | ASRs per 100000 (95%UI) |  |
| **Global** | 1276 (1185,1362) | 0·2 (0·2,0·2) | 1424 (1253,1607) | 0·2 (0·2,0·2) | -12·8 (-17·5,-8·3) |
| **Andean Latin America** | 2 (2,3) | 0·1 (0,0·1) | 7 (5,10) | 0·1 (0·1,0·1) | 84 (38·7,141·9) |
| **Bolivia (Plurinational State of)** | 0 (0,1) | 0·1 (0,0·1) | 2 (1,3) | 0·1 (0·1,0·2) | 73·3 (-2·6,235·8) |
| **Ecuador** | 1 (1,1) | 0·1 (0·1,0·1) | 3 (2,3) | 0·1 (0·1,0·2) | 66·3 (29·6,115·6) |
| **Peru** | 1 (1,1) | 0 (0,0·1) | 3 (2,5) | 0·1 (0·1,0·1) | 101·1 (32·6,199·5) |
| **Australasia** | 8 (7,8) | 0·4 (0·4,0·5) | 11 (9,12) | 0·4 (0·4,0·5) | 5·5 (-10·9,21·6) |
| **Australia** | 6 (6,7) | 0·4 (0·4,0·4) | 9 (8,10) | 0·4 (0·4,0·5) | 8·5 (-9·3,26·4) |
| **New Zealand** | 1 (1,1) | 0·5 (0·5,0·6) | 2 (1,2) | 0·5 (0·4,0·6) | -5·6 (-22,14) |
| **Caribbean** | 12 (11,13) | 0·2 (0·2,0·2) | 15 (12,20) | 0·2 (0·2,0·2) | 21·9 (5·1,40·7) |
| **Antigua and Barbuda** | 0 (0,0) | 0·2 (0·2,0·3) | 0 (0,0) | 0·3 (0·3,0·3) | 32 (11,57·4) |
| **Bahamas** | 0 (0,0) | 0·3 (0·3,0·3) | 0 (0,1) | 0·4 (0·3,0·5) | 45·5 (8·6,89·9) |
| **Barbados** | 0 (0,0) | 0·3 (0·3,0·4) | 0 (0,0) | 0·4 (0·3,0·6) | 39·8 (4·8,82·2) |
| **Belize** | 0 (0,0) | 0·1 (0,0·1) | 0 (0,0) | 0·1 (0·1,0·1) | 100·9 (67·2,138·3) |
| **Bermuda** | 0 (0,0) | 0·3 (0·2,0·3) | 0 (0,0) | 0·2 (0·2,0·3) | -19·1 (-35·3,0·7) |
| **Cuba** | 6 (6,7) | 0·2 (0·2,0·3) | 5 (4,5) | 0·3 (0·3,0·4) | 33·3 (11·7,57) |
| **Dominica** | 0 (0,0) | 0·1 (0·1,0·1) | 0 (0,0) | 0·1 (0·1,0·2) | 59·5 (9·6,130·2) |
| **Dominican Republic** | 1 (1,1) | 0 (0,0·1) | 2 (1,3) | 0·1 (0,0·1) | 42·1 (-12·3,158·7) |
| **Grenada** | 0 (0,0) | 0·3 (0·2,0·3) | 0 (0,0) | 0·3 (0·3,0·4) | 22·3 (-0·7,50·5) |
| **Guyana** | 0 (0,0) | 0·1 (0·1,0·1) | 0 (0,0) | 0·1 (0·1,0·1) | 67·8 (13·3,136·1) |
| **Haiti** | 1 (1,2) | 0·1 (0,0·2) | 4 (2,8) | 0·1 (0·1,0·3) | 43·3 (-20·2,155·6) |
| **Jamaica** | 0 (0,0) | 0·1 (0·1,0·1) | 1 (1,1) | 0·2 (0·1,0·2) | 91·3 (41·5,149·5) |
| **Puerto Rico** | 2 (2,2) | 0·3 (0·3,0·3) | 2 (1,2) | 0·3 (0·2,0·3) | 4·9 (-16·1,28·2) |
| **Saint Kitts and Nevis** | 0 (0,0) | 0·5 (0·4,0·5) | 0 (0,0) | 0·4 (0·3,0·5) | -6·8 (-25·5,14·7) |
| **Saint Lucia** | 0 (0,0) | 0·2 (0·2,0·2) | 0 (0,0) | 0·2 (0·2,0·2) | 17·6 (-6·5,44·9) |
| **Saint Vincent and the Grenadines** | 0 (0,0) | 0·1 (0·1,0·1) | 0 (0,0) | 0·1 (0·1,0·1) | 35·1 (8·5,68·5) |
| **Suriname** | 0 (0,0) | 0·1 (0·1,0·1) | 0 (0,0) | 0·1 (0·1,0·2) | 41·3 (-9·4,112·6) |
| **Trinidad and Tobago** | 0 (0,0) | 0·1 (0·1,0·1) | 1 (0,1) | 0·2 (0·1,0·2) | 43·2 (4·5,89·7) |
| **United States Virgin Islands** | 0 (0,0) | 0·2 (0·1,0·2) | 0 (0,0) | 0·2 (0·1,0·2) | -2·1 (-40·1,54·1) |
| **Central Asia** | 16 (14,18) | 0·2 (0·2,0·3) | 12 (9,15) | 0·1 (0·1,0·2) | -35·6 (-50·9,-20·1) |
| **Armenia** | 0 (0,1) | 0·2 (0·1,0·2) | 0 (0,0) | 0·2 (0·2,0·2) | 2 (-20,29·3) |
| **Azerbaijan** | 1 (0,1) | 0·1 (0·1,0·1) | 1 (0,1) | 0·1 (0,0·1) | -21·6 (-62·4,68·5) |
| **Georgia** | 0 (0,1) | 0·1 (0·1,0·1) | 0 (0,0) | 0·1 (0·1,0·1) | 16·8 (-2·2,43·3) |
| **Kazakhstan** | 10 (9,12) | 0·5 (0·4,0·6) | 5 (3,7) | 0·3 (0·2,0·3) | -50·2 (-68·4,-31·2) |
| **Kyrgyzstan** | 1 (0,1) | 0·1 (0·1,0·1) | 1 (0,1) | 0·1 (0·1,0·1) | -12·6 (-32,11·4) |
| **Mongolia** | 0 (0,1) | 0·3 (0·1,0·6) | 1 (0,2) | 0·3 (0·2,0·6) | 20 (-56·9,265·8) |
| **Tajikistan** | 0 (0,0) | 0·1 (0,0·1) | 1 (0,1) | 0·1 (0,0·1) | -11·2 (-54·3,52·8) |
| **Turkmenistan** | 1 (1,2) | 0·4 (0·3,0·5) | 1 (1,2) | 0·3 (0·2,0·4) | -28·3 (-58·2,5·8) |
| **Uzbekistan** | 2 (1,2) | 0·1 (0·1,0·1) | 3 (2,3) | 0·1 (0·1,0·1) | -10·7 (-33·9,20·6) |
| **Central Europe** | 202 (192,215) | 0·9 (0·8,1) | 65 (58,74) | 0·6 (0·5,0·6) | -38·1 (-45,-31·2) |
| **Albania** | 8 (5,12) | 1·6 (1·1,2·1) | 4 (2,7) | 1·1 (0·6,1·8) | -30·6 (-64·5,28·2) |
| **Bosnia and Herzegovina** | 4 (3,5) | 0·5 (0·4,0·7) | 1 (1,2) | 0·3 (0·2,0·5) | -37·9 (-63·4,10·9) |
| **Bulgaria** | 11 (10,12) | 0·8 (0·7,0·8) | 5 (4,7) | 0·6 (0·5,0·8) | -16·1 (-34,5·3) |
| **Croatia** | 5 (5,6) | 0·7 (0·7,0·8) | 2 (1,2) | 0·5 (0·4,0·5) | -38·5 (-49·5,-26·7) |
| **Czechia** | 15 (14,17) | 1·1 (1,1·2) | 6 (5,7) | 0·6 (0·5,0·7) | -49 (-58,-38·9) |
| **Hungary** | 15 (13,18) | 0·8 (0·8,0·9) | 4 (4,5) | 0·5 (0·4,0·6) | -38·6 (-48·1,-26·6) |
| **Montenegro** | 1 (0,1) | 0·7 (0·4,1) | 1 (0,1) | 0·7 (0·5,0·9) | -1·2 (-40·3,63) |
| **North Macedonia** | 2 (1,2) | 0·6 (0·4,0·8) | 1 (1,2) | 0·5 (0·3,0·7) | -14·7 (-46·4,37) |
| **Poland** | 89 (85,93) | 1·2 (1·2,1·3) | 23 (21,26) | 0·6 (0·6,0·7) | -46·8 (-52·8,-40) |
| **Romania** | 27 (24,30) | 0·6 (0·5,0·6) | 6 (5,7) | 0·3 (0·3,0·4) | -46·6 (-55·4,-35·2) |
| **Serbia** | 12 (8,20) | 0·8 (0·5,1·2) | 7 (4,11) | 0·7 (0·5,1) | -7·1 (-45·3,53·2) |
| **Slovakia** | 5 (3,7) | 0·6 (0·4,0·8) | 3 (2,5) | 0·5 (0·3,0·7) | -16·9 (-46·2,31·3) |
| **Slovenia** | 4 (3,4) | 1·1 (1·1,1·2) | 1 (1,1) | 0·5 (0·4,0·7) | -53·1 (-61·5,-42·4) |
| **Central Latin America** | 29 (27,30) | 0·1 (0·1,0·1) | 94 (84,106) | 0·2 (0·2,0·2) | 110·9 (84·7,140·1) |
| **Colombia** | 6 (5,6) | 0·1 (0·1,0·1) | 13 (11,15) | 0·1 (0·1,0·2) | 46·6 (18·9,78·3) |
| **Costa Rica** | 0 (0,1) | 0·1 (0·1,0·1) | 2 (2,2) | 0·2 (0·2,0·3) | 106 (75·6,138·9) |
| **El Salvador** | 0 (0,0) | 0 (0,0·1) | 1 (1,2) | 0·1 (0·1,0·1) | 128·5 (57·5,237·7) |
| **Guatemala** | 1 (1,1) | 0·1 (0·1,0·1) | 3 (3,4) | 0·1 (0·1,0·1) | 60·6 (31·7,92) |
| **Honduras** | 0 (0,0) | 0 (0,0) | 0 (0,1) | 0 (0,0) | 45·8 (-24·9,202·8) |
| **Mexico** | 17 (16,17) | 0·1 (0·1,0·1) | 60 (53,68) | 0·3 (0·2,0·3) | 134·5 (99·6,170·7) |
| **Nicaragua** | 0 (0,0) | 0·1 (0,0·1) | 1 (1,2) | 0·1 (0·1,0·1) | 88·7 (31·9,173·1) |
| **Panama** | 0 (0,0) | 0·1 (0·1,0·1) | 1 (1,1) | 0·1 (0·1,0·2) | 112·4 (62·1,167·4) |
| **Venezuela (Bolivarian Republic of)** | 3 (3,4) | 0·1 (0·1,0·1) | 13 (10,17) | 0·3 (0·2,0·3) | 121·3 (67·4,189·4) |
| **Central Sub-Saharan Africa** | 1 (0,1) | 0 (0,0) | 3 (2,5) | 0 (0,0) | 62·2 (4·5,159·6) |
| **Angola** | 0 (0,0) | 0 (0,0) | 1 (0,2) | 0 (0,0) | 93·1 (12,334·5) |
| **Central African Republic** | 0 (0,0) | 0 (0,0) | 0 (0,0) | 0 (0,0) | 31·3 (-13·6,123·8) |
| **Congo** | 0 (0,0) | 0 (0,0) | 0 (0,0) | 0 (0,0) | 52·5 (-12·3,147·6) |
| **Democratic Republic of the Congo** | 0 (0,1) | 0 (0,0) | 2 (1,3) | 0 (0,0) | 53 (-5·6,162·7) |
| **Equatorial Guinea** | 0 (0,0) | 0 (0,0) | 0 (0,0) | 0 (0,0) | 139·4 (-0·9,608·8) |
| **Gabon** | 0 (0,0) | 0 (0,0) | 0 (0,0) | 0 (0,0) | 59·3 (-16·3,242·7) |
| **East Asia** | 14 (9,22) | 0 (0,0) | 17 (13,22) | 0 (0,0) | 13·3 (-30·6,92) |
| **China** | 14 (8,21) | 0 (0,0) | 16 (12,21) | 0 (0,0) | 11·7 (-33·4,94·5) |
| **Democratic People's Republic of Korea** | 0 (0,1) | 0 (0,0) | 0 (0,1) | 0 (0,0) | 30·4 (-17·4,134·8) |
| **Taiwan (Province of China)** | 0 (0,0) | 0 (0,0) | 1 (0,1) | 0 (0,0) | 69·1 (43·9,94·5) |
| **Eastern Europe** | 358 (342,375) | 0·5 (0·5,0·6) | 142 (123,163) | 0·3 (0·3,0·4) | -37·9 (-45·3,-29·7) |
| **Belarus** | 11 (10,13) | 0·4 (0·4,0·5) | 6 (5,8) | 0·3 (0·2,0·4) | -27·1 (-44·4,-7·4) |
| **Estonia** | 4 (3,4) | 1 (0·9,1·1) | 1 (1,1) | 0·4 (0·3,0·4) | -63·4 (-70·4,-55·7) |
| **Latvia** | 7 (6,8) | 1·2 (1·1,1·3) | 2 (1,2) | 0·6 (0·5,0·7) | -50·3 (-59·5,-39·9) |
| **Lithuania** | 9 (8,10) | 1·1 (1,1·2) | 2 (2,3) | 0·6 (0·5,0·7) | -46 (-56·1,-35) |
| **Republic of Moldova** | 2 (2,3) | 0·2 (0·2,0·2) | 1 (1,1) | 0·1 (0·1,0·1) | -46·4 (-56·4,-36) |
| **Russian Federation** | 219 (210,232) | 0·5 (0·5,0·5) | 83 (75,90) | 0·3 (0·3,0·3) | -36·5 (-43·6,-28·8) |
| **Ukraine** | 105 (95,118) | 0·6 (0·6,0·7) | 47 (32,65) | 0·4 (0·3,0·5) | -36·7 (-55·8,-14·5) |
| **Eastern Sub-Saharan Africa** | 3 (1,5) | 0 (0,0) | 12 (4,18) | 0 (0,0) | 65·9 (18·2,178·2) |
| **Burundi** | 0 (0,0) | 0 (0,0) | 0 (0,0) | 0 (0,0) | 22·7 (-33,93·1) |
| **Comoros** | 0 (0,0) | 0 (0,0) | 0 (0,0) | 0 (0,0) | 78·2 (5·7,349·9) |
| **Djibouti** | 0 (0,0) | 0 (0,0) | 0 (0,0) | 0 (0,0) | 67·1 (-2·3,210) |
| **Eritrea** | 0 (0,0) | 0 (0,0) | 0 (0,1) | 0 (0,0·1) | 86·1 (13·9,248·5) |
| **Ethiopia** | 1 (0,1) | 0 (0,0) | 3 (1,5) | 0 (0,0) | 47·3 (-11·4,119·8) |
| **Kenya** | 0 (0,0) | 0 (0,0) | 1 (1,2) | 0 (0,0) | 98·4 (32·9,344·8) |
| **Madagascar** | 0 (0,0) | 0 (0,0) | 1 (0,2) | 0 (0,0) | 41·8 (-10·8,143·1) |
| **Malawi** | 0 (0,0) | 0 (0,0) | 1 (0,1) | 0 (0,0) | 92·1 (25·2,259·8) |
| **Mozambique** | 0 (0,0) | 0 (0,0) | 1 (0,2) | 0 (0,0) | 92·7 (22·3,325·1) |
| **Rwanda** | 0 (0,0) | 0 (0,0) | 0 (0,1) | 0 (0,0) | 37·9 (-22·9,119·6) |
| **Somalia** | 0 (0,0) | 0 (0,0) | 0 (0,1) | 0 (0,0) | 24·4 (-29·2,90·3) |
| **South Sudan** | 0 (0,0) | 0 (0,0) | 0 (0,0) | 0 (0,0) | 65·5 (0·9,196·7) |
| **Uganda** | 0 (0,0) | 0 (0,0) | 1 (0,2) | 0 (0,0) | 124·9 (34·5,537·7) |
| **United Republic of Tanzania** | 0 (0,1) | 0 (0,0) | 2 (1,3) | 0 (0,0) | 62·5 (-4,239·3) |
| **Zambia** | 0 (0,0) | 0 (0,0) | 1 (0,2) | 0 (0,0) | 76·4 (-5·8,303) |
| **High-income Asia Pacific** | 10 (9,11) | 0 (0,0) | 7 (6,8) | 0 (0,0) | -11·1 (-17,-5·2) |
| **Brunei Darussalam** | 0 (0,0) | 0 (0,0·1) | 0 (0,0) | 0 (0,0·1) | 8·7 (-47,94·2) |
| **Japan** | 6 (6,6) | 0 (0,0) | 5 (5,5) | 0 (0,0) | 4 (-1·6,9·4) |
| **Republic of Korea** | 4 (3,5) | 0 (0,0) | 2 (1,3) | 0 (0,0) | -42·1 (-55·6,-26) |
| **Singapore** | 0 (0,0) | 0 (0,0) | 0 (0,0) | 0 (0,0) | -44·7 (-51,-37) |
| **High-income North America** | 176 (170,182) | 0·6 (0·6,0·6) | 161 (155,168) | 0·8 (0·8,0·9) | 40·4 (31·7,49·2) |
| **Canada** | 18 (16,20) | 0·7 (0·7,0·8) | 21 (19,24) | 0·8 (0·8,0·9) | 14·6 (1·5,27·2) |
| **Greenland** | 0 (0,0) | 0·1 (0,0·1) | 0 (0,0) | 0·2 (0·1,0·3) | 202·2 (86·9,413·6) |
| **United States of America** | 158 (153,163) | 0·6 (0·5,0·6) | 140 (134,146) | 0·8 (0·8,0·9) | 43·8 (34·5,53·6) |
| **North Africa and Middle East** | 53 (30,78) | 0·1 (0·1,0·2) | 199 (161,239) | 0·2 (0·2,0·2) | 80 (24·6,214·4) |
| **Afghanistan** | 1 (0,2) | 0·1 (0,0·2) | 10 (4,19) | 0·2 (0·1,0·4) | 156·2 (54·7,455·1) |
| **Algeria** | 3 (2,5) | 0·1 (0,0·1) | 19 (11,31) | 0·2 (0·1,0·3) | 156·7 (43·2,380·3) |
| **Bahrain** | 0 (0,0) | 0 (0,0) | 0 (0,0) | 0·1 (0·1,0·1) | 996·3 (668·3,1426·4) |
| **Egypt** | 1 (0,1) | 0 (0,0) | 2 (1,3) | 0 (0,0) | 58·2 (11·7,140·6) |
| **Iran (Islamic Republic of)** | 22 (11,33) | 0·3 (0·1,0·4) | 74 (61,92) | 0·4 (0·3,0·5) | 53·4 (0·7,190·7) |
| **Iraq** | 1 (1,3) | 0·1 (0,0·1) | 6 (3,11) | 0·1 (0·1,0·1) | 56·9 (-24·1,286·9) |
| **Jordan** | 1 (1,2) | 0·2 (0·2,0·3) | 4 (3,6) | 0·2 (0·2,0·3) | -5·3 (-43·4,60·7) |
| **Kuwait** | 0 (0,0) | 0 (0,0) | 1 (0,1) | 0·1 (0,0·1) | 24716·6 (19820·5,31944·2) |
| **Lebanon** | 0 (0,1) | 0·1 (0·1,0·2) | 1 (1,2) | 0·1 (0·1,0·2) | 26 (-24·3,115·7) |
| **Libya** | 1 (0,1) | 0·1 (0·1,0·1) | 6 (3,10) | 0·4 (0·3,0·6) | 352·1 (160·9,740·5) |
| **Morocco** | 3 (1,5) | 0·1 (0,0·1) | 15 (6,28) | 0·2 (0·1,0·3) | 206·6 (72·8,650·3) |
| **Oman** | 0 (0,0) | 0·1 (0,0·1) | 2 (1,3) | 0·2 (0·1,0·3) | 162·2 (3·2,675·2) |
| **Palestine** | 0 (0,1) | 0·2 (0·1,0·2) | 2 (1,3) | 0·3 (0·2,0·4) | 105·2 (12·2,315·8) |
| **Qatar** | 0 (0,0) | 0 (0,0) | 0 (0,0) | 0 (0,0·1) | 120·1 (29·6,351·7) |
| **Saudi Arabia** | 1 (0,1) | 0 (0,0) | 8 (4,15) | 0·1 (0·1,0·1) | 188·4 (42·8,619·8) |
| **Sudan** | 1 (1,2) | 0 (0,0·1) | 11 (5,19) | 0·1 (0·1,0·2) | 177·2 (38·1,603·6) |
| **Syrian Arab Republic** | 1 (0,2) | 0·1 (0,0·1) | 2 (1,3) | 0·1 (0·1,0·1) | 54·4 (-18·5,230·6) |
| **Tunisia** | 1 (0,2) | 0·1 (0,0·1) | 6 (3,10) | 0·2 (0·1,0·4) | 169·6 (44·1,500·6) |
| **Turkey** | 14 (7,27) | 0·2 (0·1,0·2) | 25 (16,37) | 0·2 (0·2,0·3) | 32·9 (-22,152·2) |
| **United Arab Emirates** | 0 (0,0) | 0·1 (0,0·1) | 1 (0,2) | 0·1 (0,0·1) | 8·2 (-36·3,145·1) |
| **Yemen** | 0 (0,1) | 0 (0,0·1) | 5 (2,9) | 0·1 (0·1,0·2) | 176·5 (53,665·1) |
| **Oceania** | 0 (0,0) | 0 (0,0) | 0 (0,0) | 0 (0,0) | 25·6 (-11·9,89·1) |
| **American Samoa** | 0 (0,0) | 0 (0,0) | 0 (0,0) | 0 (0,0) | 39·5 (-14·2,143) |
| **Cook Islands** | 0 (0,0) | 0 (0,0) | 0 (0,0) | 0 (0,0) | 32·5 (-22·5,155) |
| **Fiji** | 0 (0,0) | 0 (0,0) | 0 (0,0) | 0 (0,0) | 31·6 (-24·4,167·8) |
| **Guam** | 0 (0,0) | 0 (0,0) | 0 (0,0) | 0 (0,0) | -15·4 (-51·7,55·7) |
| **Kiribati** | 0 (0,0) | 0 (0,0) | 0 (0,0) | 0 (0,0) | 20·1 (-29·7,109·2) |
| **Marshall Islands** | 0 (0,0) | 0 (0,0) | 0 (0,0) | 0 (0,0) | 33·9 (-23·3,178·9) |
| **Micronesia (Federated States of)** | 0 (0,0) | 0 (0,0) | 0 (0,0) | 0 (0,0) | 33·5 (-18·3,135·4) |
| **Nauru** | 0 (0,0) | 0 (0,0) | 0 (0,0) | 0 (0,0) | 35 (-23·8,144·7) |
| **Niue** | 0 (0,0) | 0 (0,0) | 0 (0,0) | 0 (0,0) | 103·3 (15·7,293·8) |
| **Northern Mariana Islands** | 0 (0,0) | 0 (0,0) | 0 (0,0) | 0 (0,0) | 21·3 (-23·5,101·1) |
| **Palau** | 0 (0,0) | 0 (0,0) | 0 (0,0) | 0 (0,0) | 55·4 (-17·8,187) |
| **Papua New Guinea** | 0 (0,0) | 0 (0,0) | 0 (0,0) | 0 (0,0) | 39·7 (-19·2,174·8) |
| **Samoa** | 0 (0,0) | 0 (0,0) | 0 (0,0) | 0 (0,0) | 31·8 (-24·8,133·9) |
| **Solomon Islands** | 0 (0,0) | 0 (0,0) | 0 (0,0) | 0 (0,0) | 50·5 (-6,230·6) |
| **Tokelau** | 0 (0,0) | 0 (0,0) | 0 (0,0) | 0 (0,0) | 115·9 (19,318·6) |
| **Tonga** | 0 (0,0) | 0 (0,0) | 0 (0,0) | 0 (0,0) | 50·9 (-12·4,200·7) |
| **Tuvalu** | 0 (0,0) | 0 (0,0) | 0 (0,0) | 0 (0,0) | 38·9 (-15·6,171·8) |
| **Vanuatu** | 0 (0,0) | 0 (0,0) | 0 (0,0) | 0 (0,0) | 47·7 (-14·5,202·9) |
| **South Asia** | 15 (7,25) | 0 (0,0) | 56 (34,75) | 0 (0,0) | 85·6 (24·9,286·3) |
| **Bangladesh** | 1 (0,3) | 0 (0,0) | 4 (1,8) | 0 (0,0) | 79·7 (5·6,315·3) |
| **Bhutan** | 0 (0,0) | 0 (0,0) | 0 (0,0) | 0 (0,0) | 99·6 (20·1,400·7) |
| **India** | 12 (6,20) | 0 (0,0) | 42 (27,56) | 0 (0,0) | 87 (22·2,291·6) |
| **Nepal** | 0 (0,0) | 0 (0,0) | 1 (0,2) | 0 (0,0) | 94·1 (24·5,342·1) |
| **Pakistan** | 2 (0,3) | 0 (0,0) | 9 (4,14) | 0 (0,0) | 82 (11·3,364·8) |
| **Southeast Asia** | 10 (7,13) | 0 (0,0) | 25 (21,30) | 0 (0,0) | 76·4 (30·9,173·4) |
| **Cambodia** | 0 (0,0) | 0 (0,0) | 0 (0,1) | 0 (0,0) | 114·8 (28·7,269·8) |
| **Indonesia** | 2 (1,2) | 0 (0,0) | 6 (4,9) | 0 (0,0) | 119·7 (33·1,286) |
| **Lao People's Democratic Republic** | 0 (0,0) | 0 (0,0) | 0 (0,0) | 0 (0,0) | 113·2 (20·5,347·2) |
| **Malaysia** | 0 (0,1) | 0 (0,0) | 2 (1,2) | 0 (0,0) | 65·2 (-8·8,239·9) |
| **Maldives** | 0 (0,0) | 0 (0,0) | 0 (0,0) | 0 (0,0) | 66 (-8·4,187) |
| **Mauritius** | 0 (0,0) | 0 (0,0) | 0 (0,0) | 0·1 (0,0·1) | 27425·1 (22127·7,33661·5) |
| **Myanmar** | 1 (0,1) | 0 (0,0) | 2 (1,3) | 0 (0,0) | 94·6 (6·5,264·9) |
| **Philippines** | 5 (4,6) | 0 (0,0) | 11 (9,13) | 0 (0,0) | 31·1 (-3·6,107·9) |
| **Seychelles** | 0 (0,0) | 0 (0,0) | 0 (0,0) | 0 (0,0) | 217·8 (64,490·1) |
| **Sri Lanka** | 0 (0,1) | 0 (0,0) | 0 (0,1) | 0 (0,0) | 8·2 (-36·3,70·9) |
| **Thailand** | 1 (0,1) | 0 (0,0) | 1 (1,2) | 0 (0,0) | 95 (24·3,242) |
| **Timor-Leste** | 0 (0,0) | 0 (0,0) | 0 (0,0) | 0 (0,0) | 136·7 (42·1,396·6) |
| **Viet Nam** | 1 (0,2) | 0 (0,0) | 3 (1,5) | 0 (0,0) | 122·2 (33·1,423·2) |
| **Southern Latin America** | 17 (16,19) | 0·3 (0·3,0·3) | 14 (12,16) | 0·2 (0·1,0·2) | -40·4 (-47·2,-33·7) |
| **Uruguay** | 2 (1,2) | 0·4 (0·4,0·5) | 1 (1,2) | 0·3 (0·3,0·4) | -25·6 (-35·6,-16) |
| **Argentina** | 13 (11,14) | 0·3 (0·3,0·3) | 10 (9,12) | 0·2 (0·2,0·2) | -36·7 (-44·3,-29) |
| **Chile** | 3 (3,3) | 0·2 (0·1,0·2) | 2 (2,2) | 0·1 (0·1,0·1) | -48·5 (-55·7,-41·3) |
| **Southern Sub-Saharan Africa** | 4 (3,6) | 0·1 (0·1,0·1) | 5 (4,7) | 0·1 (0·1,0·1) | 29·7 (4,75·7) |
| **Botswana** | 0 (0,0) | 0 (0,0) | 0 (0,0) | 0 (0,0) | 27·6 (-32·7,200·8) |
| **Eswatini** | 0 (0,0) | 0 (0,0) | 0 (0,0) | 0 (0,0) | 69·4 (-15·9,345·8) |
| **Lesotho** | 0 (0,0) | 0 (0,0) | 0 (0,0) | 0 (0,0) | 97·8 (-5,561·6) |
| **Namibia** | 0 (0,0) | 0 (0,0) | 0 (0,0) | 0 (0,0) | 61 (-9·9,228·2) |
| **South Africa** | 4 (3,6) | 0·1 (0·1,0·1) | 5 (4,7) | 0·1 (0·1,0·2) | 23·6 (-1·1,66·8) |
| **Zimbabwe** | 0 (0,0) | 0 (0,0) | 0 (0,0) | 0 (0,0) | 53·5 (-6·5,200·5) |
| **Tropical Latin America** | 18 (17,18) | 0·1 (0·1,0·1) | 33 (31,35) | 0·1 (0·1,0·1) | 20·9 (11·1,31·2) |
| **Brazil** | 17 (17,18) | 0·1 (0·1,0·1) | 32 (30,35) | 0·1 (0·1,0·1) | 19·5 (9·9,29·7) |
| **Paraguay** | 0 (0,0) | 0·1 (0,0·1) | 1 (0,1) | 0·1 (0·1,0·2) | 128·7 (59·4,236·3) |
| **Western Europe** | 254 (245,264) | 0·7 (0·6,0·7) | 201 (190,211) | 0·7 (0·7,0·8) | 10·5 (3·7,17·1) |
| **Andorra** | 0 (0,0) | 0·6 (0·4,1·1) | 0 (0,0) | 0·6 (0·4,0·9) | -5·2 (-52·3,84·9) |
| **Austria** | 5 (4,5) | 0·6 (0·6,0·7) | 4 (4,5) | 0·7 (0·7,0·8) | 15·7 (1·3,29·8) |
| **Belgium** | 7 (6,8) | 0·7 (0·6,0·7) | 7 (6,8) | 0·8 (0·7,0·8) | 12·8 (0,25·1) |
| **Cyprus** | 0 (0,0) | 0·4 (0·2,0·7) | 0 (0,1) | 0·4 (0·3,0·6) | -4·4 (-46·6,100·3) |
| **Denmark** | 5 (5,6) | 1·3 (1·2,1·5) | 4 (3,4) | 1·2 (1·1,1·4) | -8·5 (-23,7·6) |
| **Finland** | 4 (4,5) | 0·7 (0·6,0·8) | 3 (3,4) | 0·7 (0·7,0·8) | 6·4 (-8·2,21·4) |
| **France** | 33 (30,37) | 0·5 (0·5,0·6) | 24 (20,27) | 0·6 (0·5,0·6) | 6·8 (-8·6,21·5) |
| **Germany** | 66 (61,73) | 0·8 (0·7,0·8) | 33 (29,37) | 0·8 (0·8,0·9) | 7·7 (-1·6,20) |
| **Greece** | 4 (3,4) | 0·3 (0·3,0·4) | 4 (4,5) | 0·6 (0·5,0·7) | 75·6 (55·2,97·2) |
| **Iceland** | 0 (0,0) | 0·7 (0·7,0·8) | 0 (0,0) | 0·8 (0·7,0·9) | 12·9 (-2·9,31·6) |
| **Ireland** | 3 (2,3) | 1 (0·9,1) | 3 (3,4) | 0·8 (0·7,0·9) | -12·8 (-27·5,0·8) |
| **Israel** | 1 (1,1) | 0·2 (0·2,0·2) | 2 (1,2) | 0·2 (0·2,0·3) | 8 (-4·4,21·6) |
| **Italy** | 27 (25,28) | 0·4 (0·4,0·4) | 18 (17,20) | 0·5 (0·4,0·5) | 28·3 (17·8,39·1) |
| **Luxembourg** | 0 (0,0) | 0·8 (0·7,0·8) | 0 (0,0) | 0·7 (0·6,0·7) | -16·3 (-26·8,-4·7) |
| **Malta** | 0 (0,0) | 0·3 (0·2,0·3) | 0 (0,0) | 0·3 (0·3,0·4) | 16·7 (0,36·6) |
| **Monaco** | 0 (0,0) | 0·2 (0·1,0·3) | 0 (0,0) | 0·3 (0·2,0·5) | 58·8 (-13,186·4) |
| **Netherlands** | 11 (10,13) | 0·8 (0·8,0·9) | 10 (9,11) | 0·9 (0·8,0·9) | 3·9 (-6·4,15·6) |
| **Norway** | 3 (3,3) | 1·1 (1,1·2) | 3 (2,3) | 1 (0·9,1·1) | -7·2 (-14·6,1·2) |
| **Portugal** | 4 (3,4) | 0·3 (0·3,0·3) | 2 (2,3) | 0·3 (0·3,0·3) | 1·8 (-9·1,13·2) |
| **San Marino** | 0 (0,0) | 0 (0,0) | 0 (0,0) | 0 (0,0) | -22·5 (-63·1,38·6) |
| **Spain** | 13 (11,14) | 0·3 (0·3,0·3) | 8 (7,10) | 0·3 (0·3,0·3) | 8·5 (-3·4,22·2) |
| **Sweden** | 5 (4,5) | 0·7 (0·6,0·7) | 5 (4,6) | 0·8 (0·7,0·9) | 16·3 (0·4,32·5) |
| **Switzerland** | 7 (6,9) | 1·1 (1,1·2) | 5 (4,6) | 0·9 (0·8,1) | -20 (-32·9,-6·3) |
| **United Kingdom** | 56 (54,57) | 1·1 (1·1,1·1) | 65 (63,68) | 1·3 (1·3,1·4) | 20·4 (14·7,26) |
| **Western Sub-Saharan Africa** | 75 (40,113) | 0 (0,0·1) | 343 (209,499) | 0·1 (0,0·1) | 71·5 (5·6,203·7) |
| **Benin** | 2 (0,4) | 0 (0,0·1) | 9 (3,22) | 0·1 (0,0·1) | 63·7 (-54,557·3) |
| **Burkina Faso** | 3 (1,8) | 0 (0,0·1) | 14 (3,39) | 0·1 (0,0·2) | 64 (-59·5,610·4) |
| **Cabo Verde** | 0 (0,1) | 0 (0,0·1) | 0 (0,1) | 0·1 (0,0·1) | 11 (-69·7,346·6) |
| **Cameroon** | 5 (2,11) | 0 (0,0·1) | 26 (8,66) | 0·1 (0,0·2) | 55·9 (-65·6,536·8) |
| **Chad** | 1 (0,4) | 0 (0,0·1) | 8 (2,23) | 0 (0,0·1) | 76·9 (-59·8,639·3) |
| **CÃ´te d'Ivoire** | 5 (1,13) | 0 (0,0·1) | 19 (6,53) | 0·1 (0,0·2) | 78·4 (-54·1,595·1) |
| **Gambia** | 0 (0,1) | 0 (0,0·1) | 2 (1,6) | 0·1 (0,0·2) | 133·7 (-51,959·9) |
| **Ghana** | 10 (3,24) | 0·1 (0,0·1) | 35 (11,88) | 0·1 (0,0·2) | 39·4 (-62·9,450·3) |
| **Guinea** | 2 (0,5) | 0 (0,0·1) | 9 (3,24) | 0·1 (0,0·2) | 107·4 (-44·7,765·6) |
| **Guinea-Bissau** | 1 (0,1) | 0 (0,0·1) | 2 (1,5) | 0·1 (0,0·2) | 62·6 (-55,475·3) |
| **Liberia** | 1 (0,2) | 0 (0,0·1) | 4 (1,11) | 0·1 (0,0·2) | 99·7 (-47·5,608·7) |
| **Mali** | 4 (1,10) | 0 (0,0·1) | 20 (4,48) | 0·1 (0,0·2) | 60·6 (-56·6,452·3) |
| **Mauritania** | 1 (0,3) | 0 (0,0·1) | 4 (1,10) | 0·1 (0,0·2) | 69 (-57·3,553·9) |
| **Niger** | 2 (0,7) | 0 (0,0·1) | 10 (1,29) | 0 (0,0·1) | 31 (-69·1,435·6) |
| **Nigeria** | 31 (14,53) | 0 (0,0·1) | 154 (82,274) | 0·1 (0,0·1) | 90·2 (-2·9,306·7) |
| **Sao Tome and Principe** | 0 (0,0) | 0 (0,0) | 0 (0,0) | 0 (0,0) | 115·3 (-65,1061·4) |
| **Senegal** | 4 (1,10) | 0 (0,0·1) | 14 (4,37) | 0·1 (0,0·2) | 60·8 (-65·4,546·5) |
| **Sierra Leone** | 1 (0,4) | 0 (0,0·1) | 6 (2,16) | 0·1 (0,0·1) | 118·3 (-53·9,1098·5) |
| **Togo** | 2 (1,4) | 0 (0,0·1) | 6 (2,15) | 0·1 (0,0·2) | 48·2 (-62·2,417·5) |
