# Supplementary material for: Global, regional, and national trends and burden of multiple sclerosis in adolescents and young adults: a data analysis from 1990 to 2021 and projections to 2040
Source: Front Immunol. 2025 Oct 22;16:1685316. doi: 10.3389/fimmu.2025.1685316 (PMC12586062; doi:10.3389/fimmu.2025.1685316)
Supplement: Supplementary file 1 [file DataSheet1.zip › Table 4 (2).DOCX]

| **Table S4: DALYs due to MS in 1990 and 2021 and the percentage change in the age-standardised rates (ASRs) per 100,000, by location (Generated from data available from [http://ghdx·healthdata·org/gbd-results-tool](http://ghdx.healthdata.org/gbd-results-tool))** | | | | | |
| --- | --- | --- | --- | --- | --- |
|  | **1990** | | **2021** | | Percentage change in the ASRs per 100000 |
|  | No (95%UI) | ASRs per 100000 (95%UI) | No (95%UI) | ASRs per 100000 (95%UI) |  |
| **Global** | 160530 (130796,196736) | 12·8 (11·1,14·7) | 215869 (173537,268521) | 11·4 (9·8,13·2) | -11 (-14,-8) |
| **Andean Latin America** | 310 (241,401) | 3·7 (3,4·6) | 875 (653,1130) | 5·9 (4·7,7·3) | 62·1 (38·3,89·5) |
| **Bolivia (Plurinational State of)** | 63 (40,94) | 4·7 (3·2,6·8) | 195 (117,296) | 7·5 (5·3,10·5) | 58·2 (13·6,127·7) |
| **Ecuador** | 88 (73,108) | 4 (3·5,4·6) | 249 (199,306) | 6·2 (5,7·5) | 55·8 (31·9,88·4) |
| **Peru** | 159 (119,212) | 3·2 (2·5,4·1) | 431 (305,581) | 5·4 (4·1,7) | 66·1 (34·3,109·2) |
| **Australasia** | 1133 (882,1429) | 23·6 (20·1,27·4) | 1954 (1447,2579) | 28·8 (23·7,34·4) | 22·2 (9·5,36·6) |
| **Australia** | 955 (733,1226) | 23 (19·5,27) | 1722 (1262,2304) | 29·7 (24·4,36) | 29 (14,46·4) |
| **New Zealand** | 178 (135,232) | 26·2 (22·7,30·1) | 233 (177,294) | 24·1 (20·4,27·6) | -8·1 (-20·4,4·8) |
| **Caribbean** | 985 (865,1134) | 9·1 (8·2,10·4) | 1353 (1111,1683) | 10·7 (9·3,12·6) | 17·9 (5·5,31) |
| **Antigua and Barbuda** | 2 (2,3) | 11·9 (10·4,13·6) | 4 (3,5) | 15·7 (13·5,18) | 32 (14·7,52·3) |
| **Bahamas** | 12 (11,14) | 13·8 (12·4,15·2) | 29 (23,36) | 19·9 (15·8,24·4) | 44·4 (13,79·3) |
| **Barbados** | 14 (12,15) | 15·6 (14·1,17·4) | 17 (13,22) | 21·7 (17·3,26·5) | 39·2 (11·2,70·2) |
| **Belize** | 2 (2,3) | 4 (3·3,4·8) | 9 (8,11) | 6·4 (5·5,7·6) | 61·8 (43·8,84) |
| **Bermuda** | 3 (2,3) | 13 (11·6,14·6) | 2 (1,2) | 12 (10·1,14·5) | -7·4 (-20·6,7·9) |
| **Cuba** | 466 (415,523) | 12·3 (11·2,13·7) | 388 (321,466) | 15·5 (13·3,18) | 25·5 (9·3,43·5) |
| **Dominica** | 1 (1,1) | 4·6 (3·7,5·7) | 1 (1,2) | 6·6 (5,8·9) | 41·9 (11·1,81·5) |
| **Dominican Republic** | 97 (72,124) | 4·1 (3·2,5·2) | 204 (138,293) | 5·3 (4,6·9) | 30·7 (3,78·3) |
| **Grenada** | 4 (3,4) | 13·7 (12·2,15·3) | 5 (4,6) | 16·1 (13·5,18·7) | 17·8 (-0·7,39·2) |
| **Guyana** | 9 (7,11) | 3·9 (3·3,4·6) | 15 (11,19) | 6 (4·6,7·6) | 54·9 (19·1,97·4) |
| **Haiti** | 118 (75,198) | 6·2 (3·8,9·5) | 369 (223,619) | 8·2 (5,13·5) | 33·5 (-11·5,97) |
| **Jamaica** | 30 (24,37) | 5 (4·3,6) | 71 (55,92) | 8·1 (6·4,10·3) | 61·6 (31·1,98·7) |
| **Puerto Rico** | 155 (138,171) | 13·1 (12,14·5) | 122 (102,146) | 14·6 (12·1,17·4) | 11·3 (-6·1,30·9) |
| **Saint Kitts and Nevis** | 3 (2,3) | 20·8 (19·1,22·9) | 3 (2,4) | 19·4 (16·1,23·2) | -7 (-23·6,10·8) |
| **Saint Lucia** | 3 (3,4) | 8·3 (7·5,9·4) | 6 (5,7) | 10 (8·2,12) | 19·9 (1·7,41) |
| **Saint Vincent and the Grenadines** | 2 (1,2) | 5·4 (4·6,6·3) | 3 (2,3) | 7·2 (6,8·6) | 33·2 (14·8,55·9) |
| **Suriname** | 6 (4,8) | 4·8 (3·7,5·7) | 12 (8,17) | 6·5 (4·7,8·7) | 36·4 (1·6,81·8) |
| **Trinidad and Tobago** | 23 (20,27) | 6·4 (5·7,7·2) | 45 (35,58) | 9·3 (7·4,11·8) | 44·7 (14·3,80·1) |
| **United States Virgin Islands** | 3 (2,4) | 8·9 (6·4,11·5) | 3 (1,4) | 9·9 (6·8,13·7) | 10·2 (-24·9,55·6) |
| **Central Asia** | 1691 (1381,2068) | 13·2 (10·9,15·7) | 1866 (1387,2455) | 10·8 (8·4,13·7) | -18·3 (-27·9,-9·8) |
| **Armenia** | 71 (54,92) | 9·9 (7·7,12·3) | 56 (40,76) | 11·6 (9,14·4) | 17·6 (-0·5,37·6) |
| **Azerbaijan** | 115 (81,159) | 6·8 (5·2,8·9) | 167 (111,235) | 6·7 (5·1,8·9) | -0·8 (-19·1,21·2) |
| **Georgia** | 84 (61,113) | 7·2 (5·5,9·1) | 51 (34,70) | 8·7 (6·5,11) | 20·9 (5·7,39·1) |
| **Kazakhstan** | 819 (696,975) | 26·2 (21·9,30·9) | 573 (408,762) | 21·1 (15·9,26·8) | -19·8 (-35·2,-5·5) |
| **Kyrgyzstan** | 77 (59,99) | 7·7 (6·2,9·5) | 110 (81,145) | 7·2 (5·6,9·2) | -6·5 (-18·5,7) |
| **Mongolia** | 42 (25,72) | 10·6 (6·7,16·9) | 85 (51,138) | 11·9 (7·8,18·5) | 12·1 (-39·5,99·6) |
| **Tajikistan** | 61 (43,86) | 5·8 (4·3,7·8) | 137 (91,197) | 5·6 (4·1,7·6) | -3·9 (-22·6,18·2) |
| **Turkmenistan** | 112 (91,137) | 16·7 (13·8,19·5) | 142 (96,193) | 15·1 (10·7,19·1) | -9·6 (-32·9,13·9) |
| **Uzbekistan** | 310 (222,418) | 8·6 (6·6,11·1) | 545 (382,743) | 7 (5·4,8·9) | -18·4 (-29·9,-6·5) |
| **Central Europe** | 15909 (14141,17986) | 43·3 (40,47·1) | 8182 (6642,9853) | 29·9 (26·3,33·7) | -31·1 (-36·7,-25·5) |
| **Albania** | 586 (411,819) | 72·8 (55·5,93) | 341 (208,551) | 56·7 (40·3,82·3) | -22·2 (-49·5,17) |
| **Bosnia and Herzegovina** | 360 (272,464) | 26·4 (20·7,33·6) | 138 (101,189) | 18·7 (13·8,25) | -29 (-49·8,5) |
| **Bulgaria** | 839 (724,960) | 38·6 (34·5,43·2) | 502 (408,624) | 35·6 (30·3,41·7) | -7·9 (-22,8·3) |
| **Croatia** | 409 (361,468) | 31·1 (28·1,34·6) | 203 (157,254) | 23·3 (19·7,27·1) | -25·1 (-36·4,-13) |
| **Czechia** | 1191 (1025,1405) | 48·6 (44·3,53·6) | 642 (507,805) | 28 (23·4,33·1) | -42·4 (-50·9,-33·2) |
| **Hungary** | 1277 (1086,1492) | 42·8 (38·4,47·8) | 540 (416,679) | 27 (23·1,31·2) | -36·9 (-44·5,-26·7) |
| **Montenegro** | 62 (44,87) | 34·9 (25·5,47·3) | 54 (40,70) | 35·3 (27·4,44) | 1·3 (-31·2,45·7) |
| **North Macedonia** | 154 (115,201) | 28·2 (21·9,35·7) | 152 (110,204) | 27·2 (21·2,34·8) | -3·3 (-26·5,29·6) |
| **Poland** | 7113 (6283,8003) | 60 (55·3,65·1) | 3594 (2860,4368) | 36·3 (31·3,41) | -39·5 (-45·1,-33·5) |
| **Romania** | 1942 (1719,2178) | 26·7 (24·1,29·4) | 622 (502,753) | 15·4 (13,17·7) | -42·3 (-51·1,-33·4) |
| **Serbia** | 1052 (736,1494) | 37·9 (28,52·3) | 829 (567,1112) | 38·4 (28·8,49·5) | 1·3 (-30·7,41·6) |
| **Slovakia** | 399 (298,539) | 26·5 (20·9,33·9) | 327 (233,448) | 23·5 (17·9,31·4) | -11 (-36·8,25·2) |
| **Slovenia** | 272 (235,313) | 50·6 (45·8,55·6) | 119 (92,152) | 28·5 (23·5,33·9) | -43·6 (-51·7,-34·2) |
| **Central Latin America** | 2554 (2227,2964) | 5·5 (4·9,6·2) | 7656 (6611,8873) | 10·5 (9·2,11·9) | 91·2 (71·8,111·6) |
| **Colombia** | 478 (418,541) | 4·8 (4·3,5·3) | 1027 (852,1216) | 6·8 (5·7,8·1) | 43·7 (22,66) |
| **Costa Rica** | 44 (37,53) | 5·8 (5·1,6·7) | 147 (122,174) | 10·8 (9·4,12·4) | 87·7 (65·3,111·9) |
| **El Salvador** | 42 (32,54) | 3·1 (2·5,3·8) | 99 (75,130) | 5·7 (4·4,7·1) | 83·9 (48·2,137·1) |
| **Guatemala** | 87 (75,103) | 4·1 (3·6,4·7) | 286 (234,347) | 6·3 (5·3,7·6) | 55·6 (37·2,75·1) |
| **Honduras** | 24 (17,34) | 2·1 (1·6,2·8) | 79 (54,116) | 2·9 (2·1,3·8) | 34·3 (8·5,67·2) |
| **Mexico** | 1527 (1326,1777) | 6·3 (5·6,7·2) | 4909 (4178,5697) | 13 (11·2,14·9) | 107·7 (84·8,132·3) |
| **Nicaragua** | 34 (26,44) | 3·5 (2·9,4·4) | 111 (82,149) | 6·1 (4·7,7·6) | 70·7 (38·2,112) |
| **Panama** | 26 (21,31) | 3·7 (3·2,4·4) | 82 (65,100) | 6·7 (5·4,8·1) | 80 (48·8,114·4) |
| **Venezuela (Bolivarian Republic of)** | 291 (251,337) | 5·8 (5·2,6·5) | 916 (710,1166) | 11·9 (9·4,14·9) | 105·4 (62·3,160·4) |
| **Central Sub-Saharan Africa** | 236 (161,347) | 1·6 (1·1,2·2) | 727 (495,1022) | 1·9 (1·4,2·6) | 23·3 (5·2,43·3) |
| **Angola** | 53 (36,79) | 1·8 (1·2,2·6) | 203 (133,300) | 2·4 (1·6,3·3) | 32·5 (10,61·4) |
| **Central African Republic** | 12 (8,19) | 1·6 (1·1,2·3) | 28 (19,40) | 1·8 (1·2,2·5) | 10·6 (-3·9,31·4) |
| **Congo** | 11 (7,15) | 1·7 (1·2,2·2) | 34 (23,47) | 2·1 (1·6,2·8) | 25·6 (2,58·3) |
| **Democratic Republic of the Congo** | 154 (103,230) | 1·5 (1,2·1) | 440 (293,625) | 1·8 (1·2,2·4) | 18·7 (1,43·6) |
| **Equatorial Guinea** | 2 (1,2) | 1·5 (1,2·1) | 10 (7,14) | 2·2 (1·6,3) | 51·2 (8·6,121·5) |
| **Gabon** | 4 (3,6) | 1·7 (1·3,2·2) | 11 (8,16) | 2·2 (1·6,2·9) | 30·3 (0,80·6) |
| **East Asia** | 3703 (2513,5419) | 0·6 (0·5,0·9) | 4573 (3163,6426) | 0·9 (0·6,1·1) | 35·7 (14·2,55·7) |
| **China** | 3519 (2383,5146) | 0·6 (0·5,0·9) | 4241 (2912,6008) | 0·8 (0·6,1·1) | 33·6 (11·5,54) |
| **Democratic People's Republic of Korea** | 100 (66,149) | 1·1 (0·8,1·5) | 144 (95,204) | 1·3 (0·9,1·8) | 19 (3·6,39·7) |
| **Taiwan (Province of China)** | 85 (61,116) | 0·9 (0·7,1·2) | 188 (130,246) | 2·2 (1·6,2·8) | 137·1 (112·4,171·7) |
| **Eastern Europe** | 25878 (23698,28604) | 28·7 (26·6,31·1) | 13759 (11693,15783) | 20·4 (17·7,22·8) | -28·8 (-36·1,-21·8) |
| **Belarus** | 770 (682,871) | 21 (19,23·3) | 467 (376,575) | 16·5 (13·3,20) | -21·3 (-35·9,-5·2) |
| **Estonia** | 237 (210,267) | 45·6 (41·5,49·6) | 64 (52,76) | 18·5 (15·5,21·5) | -59·4 (-66·6,-52·4) |
| **Latvia** | 442 (390,502) | 51·4 (46·9,56) | 126 (103,150) | 27·1 (23,31·3) | -47·2 (-56,-38·1) |
| **Lithuania** | 574 (511,648) | 48·3 (42·9,54·1) | 177 (146,206) | 27·2 (22·7,31·6) | -43·6 (-53·2,-33) |
| **Republic of Moldova** | 161 (139,183) | 10 (9,11·1) | 83 (67,100) | 6·9 (5·7,8·2) | -31·7 (-41·9,-20·9) |
| **Russian Federation** | 16558 (15002,18460) | 27 (24·8,29·7) | 9048 (7662,10474) | 19·8 (17·5,22·2) | -26·6 (-33·2,-19·8) |
| **Ukraine** | 7136 (6302,8021) | 33·5 (30·6,37) | 3795 (2837,4896) | 23·8 (17·8,30·1) | -29·2 (-45·9,-11·2) |
| **Eastern Sub-Saharan Africa** | 898 (607,1326) | 1·7 (1·2,2·4) | 2552 (1697,3593) | 2 (1·4,2·7) | 19·8 (8·2,35·7) |
| **Burundi** | 24 (16,36) | 1·5 (1,2·1) | 65 (43,92) | 1·6 (1,2·2) | 5·3 (-8·2,25·6) |
| **Comoros** | 2 (2,4) | 1·9 (1·3,2·7) | 6 (4,8) | 2·4 (1·7,3·3) | 24·4 (4·3,53·3) |
| **Djibouti** | 2 (1,3) | 1·9 (1·2,2·7) | 10 (6,14) | 2·3 (1·5,3·3) | 23·5 (2·5,53·7) |
| **Eritrea** | 19 (12,28) | 2 (1·4,2·9) | 52 (34,77) | 2·6 (1·7,3·6) | 27·6 (8·4,57·6) |
| **Ethiopia** | 244 (165,365) | 1·7 (1·2,2·5) | 649 (421,946) | 2 (1·3,2·7) | 12·8 (-4·1,35·8) |
| **Kenya** | 91 (61,130) | 1·5 (1,2) | 299 (213,403) | 2 (1·5,2·6) | 34 (17·8,57·1) |
| **Madagascar** | 75 (49,107) | 2·1 (1·5,3) | 218 (144,313) | 2·4 (1·6,3·4) | 12·7 (-2·6,36·7) |
| **Malawi** | 51 (34,77) | 1·8 (1·2,2·5) | 130 (81,196) | 2·3 (1·4,3·3) | 25·1 (9·1,50·3) |
| **Mozambique** | 75 (48,111) | 2 (1·3,2·8) | 215 (127,333) | 2·5 (1·6,3·6) | 26·4 (9·1,56·5) |
| **Rwanda** | 34 (23,48) | 1·6 (1·2,2·2) | 77 (51,112) | 1·8 (1·3,2·5) | 15·3 (-5·3,42·4) |
| **Somalia** | 33 (21,50) | 1·4 (1,2·1) | 91 (59,132) | 1·5 (1,2·1) | 5·7 (-6·4,22·1) |
| **South Sudan** | 25 (16,37) | 1·5 (1,2·1) | 45 (29,66) | 1·8 (1·1,2·5) | 18·3 (2·6,44·9) |
| **Uganda** | 60 (39,90) | 1·3 (0·9,1·8) | 201 (132,288) | 1·7 (1·1,2·4) | 30·5 (13·8,59·6) |
| **United Republic of Tanzania** | 117 (77,170) | 1·7 (1·1,2·3) | 339 (224,487) | 2 (1·4,2·8) | 21·1 (1,48·7) |
| **Zambia** | 44 (29,65) | 2 (1·3,2·8) | 153 (102,216) | 2·5 (1·8,3·5) | 27·2 (0·6,56·1) |
| **High-income Asia Pacific** | 1917 (1429,2553) | 3·7 (2·9,4·7) | 1523 (1096,2006) | 3·7 (2·8,4·7) | -0·3 (-3·8,2·7) |
| **Brunei Darussalam** | 2 (1,3) | 2·1 (1·4,3·3) | 4 (2,6) | 2·3 (1·4,3·7) | 7·7 (-14·1,44·2) |
| **Japan** | 1236 (898,1654) | 3·6 (2·8,4·6) | 1001 (728,1320) | 3·9 (3,4·9) | 6·7 (3·7,9·6) |
| **Republic of Korea** | 658 (493,873) | 3·9 (3·1,5) | 490 (355,658) | 3·4 (2·6,4·5) | -12·3 (-20·4,-4·7) |
| **Singapore** | 21 (15,29) | 1·8 (1·4,2·3) | 28 (19,40) | 1·6 (1·2,2·1) | -11·9 (-18,-6·6) |
| **High-income North America** | 35119 (26940,44246) | 44 (36·7,51·9) | 38917 (30004,48310) | 49·2 (41·8,56·5) | 11·8 (5·6,18·5) |
| **Canada** | 3879 (2952,4869) | 52·1 (44·3,60·5) | 4717 (3555,5865) | 59·2 (49,69·1) | 13·7 (4·9,23·2) |
| **Greenland** | 6 (4,8) | 16·5 (11·9,21·5) | 5 (3,7) | 21·5 (16·6,27·9) | 30·1 (9·1,58·3) |
| **United States of America** | 31233 (23765,39779) | 43·1 (35·9,51·1) | 34195 (26374,42720) | 48 (40·8,55·2) | 11·4 (4·8,18·9) |
| **North Africa and Middle East** | 12059 (8953,16145) | 12·6 (9·7,16·5) | 35679 (27758,45344) | 17·9 (14·5,21·7) | 41·5 (24,63·3) |
| **Afghanistan** | 241 (159,351) | 11·6 (7·9,16·6) | 1645 (1118,2365) | 19·7 (13·9,27) | 69·4 (35·8,111·3) |
| **Algeria** | 820 (559,1142) | 11·4 (8·4,15·6) | 3056 (2136,4179) | 20·4 (16·1,26·2) | 79·3 (40·9,121·1) |
| **Bahrain** | 16 (10,23) | 7 (4·7,9·7) | 73 (49,103) | 12·7 (9·6,16·5) | 80·2 (50·9,115·6) |
| **Egypt** | 817 (545,1146) | 4·8 (3·3,6·6) | 3450 (2159,4952) | 9·7 (6·6,13·6) | 100·6 (71·5,133·2) |
| **Iran (Islamic Republic of)** | 3470 (2440,4608) | 22·9 (16·7,29·7) | 8549 (6957,10532) | 28 (23·3,33·1) | 22·1 (-0·1,54·7) |
| **Iraq** | 496 (335,722) | 9·8 (7·2,13·4) | 1787 (1175,2510) | 13·6 (9·7,17·9) | 38·8 (15,69) |
| **Jordan** | 200 (147,273) | 20·9 (16·5,26·8) | 836 (595,1128) | 21·7 (17,27·4) | 3·8 (-17·5,33·4) |
| **Kuwait** | 62 (40,92) | 8·2 (5·5,11·4) | 360 (240,513) | 15·9 (11·5,21) | 94·9 (66·8,132·2) |
| **Lebanon** | 113 (76,162) | 13·3 (9·8,18·1) | 399 (274,572) | 19·6 (15·1,25·7) | 47·3 (19·1,79·1) |
| **Libya** | 131 (91,187) | 11·3 (8·4,14·8) | 679 (457,957) | 27·9 (21·3,36·8) | 147·4 (86·2,237·1) |
| **Morocco** | 847 (572,1198) | 10·7 (7·5,14·6) | 2420 (1600,3446) | 20·4 (15·2,27·5) | 90·4 (48·8,145·5) |
| **Oman** | 60 (40,86) | 9·4 (6·9,12·5) | 355 (239,496) | 17·4 (12·3,23·1) | 85·2 (37·4,161·8) |
| **Palestine** | 67 (45,95) | 13·8 (10·1,18·7) | 337 (244,444) | 22·9 (18·2,28·6) | 66·3 (26·6,113·4) |
| **Qatar** | 23 (14,35) | 10·2 (6·8,14·4) | 298 (196,420) | 17·4 (12·4,22·7) | 71·2 (44·2,106·7) |
| **Saudi Arabia** | 351 (230,513) | 6·9 (4·8,9·3) | 1977 (1319,2814) | 11·3 (8·5,14·9) | 64 (36·7,99·9) |
| **Sudan** | 399 (274,563) | 7·1 (5·1,9·6) | 1771 (1237,2423) | 12·7 (9·6,16·2) | 78·7 (40·3,142·4) |
| **Syrian Arab Republic** | 340 (231,472) | 10 (7·3,13·3) | 536 (363,767) | 14·7 (10·9,19·7) | 47·4 (20,78·3) |
| **Tunisia** | 317 (220,458) | 12·5 (9·1,17·1) | 894 (601,1242) | 23·1 (17·1,29·8) | 84·3 (49·9,135·9) |
| **Turkey** | 2998 (2188,4079) | 17·6 (13·4,22·9) | 4738 (3552,6143) | 19·4 (15·5,23·7) | 10·3 (-10·6,36·5) |
| **United Arab Emirates** | 66 (43,95) | 7·9 (5·6,10·8) | 400 (253,582) | 8·3 (6·2,10·9) | 6·1 (-12·5,31·7) |
| **Yemen** | 221 (142,328) | 6·7 (4·7,9·4) | 1086 (743,1576) | 11·3 (8·3,15·6) | 69 (39·7,114·6) |
| **Oceania** | 13 (8,21) | 0·5 (0·3,0·7) | 29 (17,45) | 0·5 (0·3,0·7) | 0·7 (-2·6,4·3) |
| **American Samoa** | 0 (0,0) | 0·5 (0·4,0·8) | 0 (0,0) | 0·6 (0·4,0·8) | 4·8 (0·2,10) |
| **Cook Islands** | 0 (0,0) | 0·7 (0·4,1) | 0 (0,0) | 0·7 (0·5,1) | 6·8 (1·4,11·9) |
| **Fiji** | 2 (1,3) | 0·6 (0·4,0·8) | 2 (1,4) | 0·6 (0·4,0·8) | 4·2 (-0·5,9·5) |
| **Guam** | 0 (0,1) | 0·6 (0·4,0·8) | 0 (0,1) | 0·6 (0·4,0·8) | 3·2 (-1·2,8·5) |
| **Kiribati** | 0 (0,0) | 0·4 (0·3,0·6) | 0 (0,0) | 0·5 (0·3,0·7) | 11·2 (5·5,17·8) |
| **Marshall Islands** | 0 (0,0) | 0·5 (0·3,0·7) | 0 (0,0) | 0·5 (0·3,0·7) | 8 (2·2,14·6) |
| **Micronesia (Federated States of)** | 0 (0,0) | 0·5 (0·3,0·7) | 0 (0,0) | 0·5 (0·3,0·7) | 9·8 (4·4,15·7) |
| **Nauru** | 0 (0,0) | 0·4 (0·3,0·6) | 0 (0,0) | 0·4 (0·3,0·6) | 8·6 (4·4,14·8) |
| **Niue** | 0 (0,0) | 0·7 (0·4,0·9) | 0 (0,0) | 0·7 (0·5,1) | 5·7 (0·7,11·3) |
| **Northern Mariana Islands** | 0 (0,0) | 0·6 (0·4,0·9) | 0 (0,0) | 0·7 (0·4,0·9) | 7·2 (2·3,12·3) |
| **Palau** | 0 (0,0) | 0·5 (0·3,0·7) | 0 (0,0) | 0·5 (0·3,0·7) | 6·1 (1,11·1) |
| **Papua New Guinea** | 7 (4,12) | 0·4 (0·3,0·6) | 20 (12,32) | 0·4 (0·3,0·6) | 3·2 (-1·9,8·6) |
| **Samoa** | 0 (0,1) | 0·6 (0·4,0·8) | 0 (0,1) | 0·6 (0·4,0·8) | 6 (1·2,11) |
| **Solomon Islands** | 1 (0,1) | 0·6 (0·4,0·8) | 2 (1,3) | 0·6 (0·4,0·9) | 11 (5·6,18·7) |
| **Tokelau** | 0 (0,0) | 0·5 (0·3,0·7) | 0 (0,0) | 0·5 (0·4,0·8) | 7·5 (3·1,12·3) |
| **Tonga** | 0 (0,0) | 0·6 (0·4,0·9) | 0 (0,0) | 0·7 (0·5,1) | 6·2 (1·4,11) |
| **Tuvalu** | 0 (0,0) | 0·5 (0·3,0·7) | 0 (0,0) | 0·5 (0·3,0·7) | 5·4 (0·7,11·2) |
| **Vanuatu** | 0 (0,1) | 0·5 (0·3,0·8) | 1 (0,1) | 0·6 (0·4,0·8) | 6·3 (0·4,11·9) |
| **South Asia** | 8001 (5408,11623) | 2·3 (1·7,3·2) | 19216 (13387,26558) | 3 (2·2,3·9) | 28·4 (18·5,39·2) |
| **Bangladesh** | 730 (477,1081) | 2·2 (1·6,3·1) | 1562 (1063,2233) | 2·8 (2,3·7) | 22·7 (10,39·6) |
| **Bhutan** | 5 (3,7) | 2·4 (1·7,3·3) | 9 (6,12) | 3 (2·2,4·1) | 28·1 (15·9,46·5) |
| **India** | 6287 (4258,9095) | 2·3 (1·6,3·1) | 14499 (10087,19985) | 2·9 (2·2,3·8) | 28·8 (17·4,40·7) |
| **Nepal** | 141 (93,208) | 2·4 (1·7,3·3) | 324 (217,467) | 3 (2·2,4·2) | 28·9 (17·7,44·2) |
| **Pakistan** | 838 (554,1215) | 2·7 (1·9,3·8) | 2822 (1974,3947) | 3·5 (2·5,4·6) | 26·5 (15,41·1) |
| **Southeast Asia** | 1862 (1353,2596) | 0·9 (0·7,1·2) | 3487 (2702,4544) | 1·2 (0·9,1·4) | 30·8 (16·5,49·4) |
| **Cambodia** | 29 (20,43) | 0·7 (0·5,1) | 74 (48,111) | 1 (0·7,1·3) | 34·1 (15·6,64) |
| **Indonesia** | 562 (380,812) | 0·7 (0·5,0·9) | 1098 (776,1500) | 0·9 (0·7,1·2) | 32·4 (15,60·3) |
| **Lao People's Democratic Republic** | 13 (8,20) | 0·8 (0·5,1·1) | 36 (23,53) | 1 (0·7,1·4) | 30·2 (12,60·4) |
| **Malaysia** | 66 (43,98) | 0·9 (0·6,1·3) | 169 (122,228) | 1·3 (1,1·6) | 36·3 (0·8,86·9) |
| **Maldives** | 0 (0,1) | 0·6 (0·4,0·9) | 2 (1,3) | 0·7 (0·5,1) | 17·3 (0·7,41·4) |
| **Mauritius** | 4 (2,6) | 0·7 (0·5,1) | 15 (13,17) | 2·9 (2·6,3·4) | 303·1 (207·9,468·6) |
| **Myanmar** | 171 (116,254) | 0·9 (0·6,1·2) | 280 (194,398) | 1·1 (0·8,1·6) | 28·6 (6·7,57·8) |
| **Philippines** | 474 (364,610) | 1·7 (1·3,2·1) | 986 (799,1216) | 2 (1·7,2·4) | 18·5 (-2·2,54·8) |
| **Seychelles** | 0 (0,0) | 0·8 (0·6,1·1) | 1 (0,1) | 1·2 (0·9,1·7) | 54·7 (30·8,89·6) |
| **Sri Lanka** | 73 (52,104) | 0·9 (0·7,1·2) | 83 (58,117) | 1 (0·8,1·3) | 9·7 (-6·6,29·8) |
| **Thailand** | 217 (144,315) | 0·8 (0·6,1·1) | 250 (177,342) | 1 (0·8,1·4) | 26·6 (12·4,46·6) |
| **Timor-Leste** | 2 (1,3) | 0·6 (0·4,0·8) | 4 (3,6) | 0·8 (0·5,1·1) | 31·7 (16·3,56·9) |
| **Viet Nam** | 247 (158,378) | 0·9 (0·6,1·3) | 484 (321,699) | 1·2 (0·9,1·7) | 38·6 (17·7,68·5) |
| **Southern Latin America** | 1928 (1579,2387) | 15·2 (13·3,17·7) | 2189 (1630,2779) | 11·4 (9·4,13·7) | -24·8 (-32,-17·8) |
| **Uruguay** | 152 (126,180) | 21·1 (18·7,23·7) | 154 (124,191) | 17·9 (15·3,20·9) | -15·2 (-24·4,-4·4) |
| **Argentina** | 1374 (1125,1687) | 16·4 (14·3,19·2) | 1572 (1167,1977) | 12·4 (10·2,14·9) | -23·9 (-31·9,-15·6) |
| **Chile** | 402 (313,515) | 10·4 (8·7,12·4) | 462 (329,624) | 8·1 (6·4,10·2) | -22·2 (-31·2,-12) |
| **Southern Sub-Saharan Africa** | 576 (439,751) | 4·6 (3·6,5·7) | 886 (672,1148) | 5·3 (4·3,6·4) | 14·9 (-0·4,34·1) |
| **Botswana** | 7 (5,10) | 2 (1·4,2·7) | 19 (13,28) | 2·2 (1·6,3) | 11·3 (-3·6,27·2) |
| **Eswatini** | 5 (3,7) | 2·2 (1·6,2·9) | 9 (6,13) | 2·6 (1·9,3·4) | 18·1 (0·9,41·8) |
| **Lesotho** | 9 (6,13) | 2·2 (1·5,3) | 15 (10,22) | 2·6 (1·9,3·5) | 18·7 (4,38·7) |
| **Namibia** | 8 (5,11) | 2 (1·4,2·7) | 17 (11,24) | 2·3 (1·7,3·1) | 18·6 (4·2,38·3) |
| **South Africa** | 502 (392,642) | 5·5 (4·3,6·8) | 742 (577,944) | 6·2 (5·1,7·5) | 12·1 (-3·9,31·9) |
| **Zimbabwe** | 46 (28,69) | 1·5 (1,2·1) | 84 (52,123) | 1·6 (1·1,2·2) | 7·1 (1,13·9) |
| **Tropical Latin America** | 2581 (1985,3307) | 7·7 (6·3,9·5) | 4715 (3675,6054) | 9·5 (7·8,11·5) | 23·7 (17·3,31·4) |
| **Brazil** | 2534 (1950,3241) | 7·8 (6·3,9·6) | 4591 (3565,5898) | 9·6 (7·9,11·6) | 23·4 (17,31·4) |
| **Paraguay** | 47 (34,64) | 5·1 (3·9,6·8) | 125 (88,169) | 7·3 (5·8,9·4) | 42·3 (20,73·4) |
| **Western Europe** | 37087 (29297,45959) | 39·1 (33·9,44·8) | 39336 (30393,49273) | 45·3 (38·2,52·1) | 15·9 (10·5,20·4) |
| **Andorra** | 6 (4,8) | 37·3 (27·8,52·2) | 8 (5,10) | 40·6 (29·4,54·7) | 8·6 (-27·1,59·9) |
| **Austria** | 672 (515,860) | 35·9 (31,42·1) | 790 (581,1016) | 43·4 (36,50·6) | 20·8 (7·7,34·5) |
| **Belgium** | 942 (733,1183) | 38·9 (33·7,44·4) | 1057 (794,1387) | 45·2 (37·7,52·9) | 16·2 (4·2,28·8) |
| **Cyprus** | 36 (24,54) | 21 (13·6,30·3) | 88 (62,121) | 25·2 (19·7,32·2) | 20·2 (-16,76) |
| **Denmark** | 747 (578,941) | 69·8 (62·1,79·4) | 676 (501,886) | 65·8 (56·6,76·1) | -5·7 (-16·8,5·3) |
| **Finland** | 530 (416,656) | 40·3 (35·1,45·9) | 491 (379,627) | 44·1 (37·6,50·7) | 9·3 (-1,20) |
| **France** | 5336 (3992,6869) | 33·4 (28·3,39·3) | 5871 (4236,7600) | 40·4 (33·2,47·8) | 20·8 (10·3,32·6) |
| **Germany** | 8934 (6966,11412) | 43·8 (38,51) | 7304 (5439,9702) | 47·2 (40·3,54·9) | 7·7 (-2·1,18·3) |
| **Greece** | 444 (365,549) | 17·8 (15·5,20·3) | 503 (394,622) | 29·2 (25·5,33·1) | 64·3 (48·3,81·9) |
| **Iceland** | 33 (25,40) | 49·5 (41·6,56·4) | 45 (35,58) | 54·7 (45·5,64·9) | 10·5 (-2·9,23·4) |
| **Ireland** | 482 (360,636) | 58·2 (49·1,67) | 658 (476,856) | 58·8 (47·8,70) | 1 (-10·4,12·2) |
| **Israel** | 186 (136,246) | 15·2 (12·6,18·3) | 368 (261,495) | 17·1 (14,20·5) | 12·1 (1·3,25·2) |
| **Italy** | 4817 (3729,6101) | 29·6 (24·6,35·4) | 4534 (3343,5916) | 36·8 (30,44·6) | 24·2 (17·4,30·5) |
| **Luxembourg** | 45 (35,58) | 44·4 (37·7,51·7) | 65 (46,86) | 42·6 (35·6,50·1) | -4 (-14·6,7·2) |
| **Malta** | 11 (9,14) | 13·6 (11·9,15·7) | 15 (11,19) | 17·2 (14·7,20·3) | 26·6 (13·2,41·6) |
| **Monaco** | 1 (1,2) | 18·1 (13·7,23·5) | 2 (1,3) | 24·9 (18·3,33) | 37·1 (5·6,81·4) |
| **Netherlands** | 1762 (1320,2249) | 47·1 (40·7,54) | 1694 (1243,2144) | 50·9 (42·8,58·8) | 8·1 (-1·3,18·9) |
| **Norway** | 456 (358,578) | 55·7 (49·1,63) | 615 (444,799) | 62·9 (52·3,74·2) | 13 (4·4,21·5) |
| **Portugal** | 482 (377,606) | 18·4 (15·6,21·6) | 353 (278,433) | 18·4 (15·8,21·3) | -0·1 (-10·6,11·4) |
| **San Marino** | 1 (1,1) | 9·8 (6·4,13·9) | 1 (1,1) | 10·7 (7·2,15·2) | 9·7 (-6·1,30·6) |
| **Spain** | 2509 (1871,3177) | 22·3 (18·5,26·7) | 2859 (2013,3869) | 28·5 (22·8,34·4) | 27·6 (13·4,43·3) |
| **Sweden** | 1066 (788,1367) | 53·6 (44·1,64·8) | 1391 (980,1840) | 63·5 (51,77·8) | 18·5 (8·4,27) |
| **Switzerland** | 931 (740,1154) | 56 (48·5,64·7) | 911 (670,1193) | 49·7 (41·8,57·8) | -11·2 (-22·1,-1) |
| **United Kingdom** | 6629 (5456,7981) | 57·1 (51·2,63·5) | 9004 (7203,10947) | 71·3 (61·8,80·8) | 24·9 (19·6,29·2) |
| **Western Sub-Saharan Africa** | 6087 (3592,8747) | 4·2 (2·9,5·7) | 26395 (17195,37211) | 6·4 (4·7,8·5) | 52·5 (13·4,109) |
| **Benin** | 138 (52,314) | 4·1 (2·2,7·8) | 671 (250,1558) | 5·7 (3,11·4) | 39·6 (-36·8,213·7) |
| **Burkina Faso** | 254 (81,611) | 4·1 (2·1,8) | 1060 (353,2800) | 5·7 (2·7,12·3) | 37·7 (-37·9,217·9) |
| **Cabo Verde** | 16 (5,38) | 5·5 (2·6,10·5) | 28 (10,69) | 6·1 (3·1,12·4) | 11·8 (-49·5,140·8) |
| **Cameroon** | 390 (169,817) | 4·7 (2·6,8·5) | 1932 (688,4695) | 6·6 (3·1,14·2) | 40·9 (-46·5,275·5) |
| **Chad** | 133 (46,328) | 3·6 (2,6·9) | 656 (213,1616) | 5 (2·5,9·8) | 38·4 (-35·3,188·5) |
| **CÃ´te d'Ivoire** | 392 (149,931) | 3·9 (2·1,7·9) | 1463 (542,3736) | 5·9 (2·9,13·1) | 50·4 (-39·3,272·5) |
| **Gambia** | 32 (11,79) | 4·2 (2·2,8·2) | 180 (60,417) | 7·6 (3·4,15·6) | 83·2 (-28·9,345) |
| **Ghana** | 827 (345,1772) | 7·3 (4·1,13·1) | 2857 (1169,6468) | 11·2 (6·8,20·3) | 54·3 (-27·2,237·2) |
| **Guinea** | 146 (57,338) | 3·8 (2·1,7·1) | 706 (264,1691) | 6·2 (3·1,12·8) | 62·2 (-26·5,250·4) |
| **Guinea-Bissau** | 41 (16,92) | 5·2 (2·7,10·1) | 141 (58,331) | 7·4 (3·8,15) | 44 (-38·9,216·1) |
| **Liberia** | 72 (29,164) | 3·9 (2·2,7·6) | 327 (118,774) | 6·4 (2·9,13·6) | 64·3 (-32·4,257·2) |
| **Mali** | 312 (101,749) | 5·1 (2·5,10·3) | 1478 (456,3402) | 7·1 (3·3,14) | 38·4 (-38·6,188·2) |
| **Mauritania** | 88 (39,192) | 5·7 (3·4,10·2) | 311 (121,706) | 8·2 (4·2,16·2) | 44 (-38·8,222·5) |
| **Niger** | 203 (59,517) | 4 (2,7·8) | 848 (215,2094) | 4·7 (2·3,9·4) | 19·6 (-43·3,159·8) |
| **Nigeria** | 2508 (1409,3942) | 3·6 (2·4,5·2) | 11740 (6803,19930) | 5·9 (3·9,9) | 62·9 (7·6,160·1) |
| **Sao Tome and Principe** | 1 (1,2) | 1·9 (1·2,2·9) | 4 (2,8) | 2·7 (1·7,4·4) | 41·3 (-19·6,150·1) |
| **Senegal** | 288 (94,727) | 4·9 (2·5,10·2) | 1028 (372,2606) | 7·1 (3·6,15·4) | 42·6 (-47,240) |
| **Sierra Leone** | 103 (39,275) | 3·4 (1·9,7) | 481 (173,1129) | 5·6 (2·8,11·3) | 66·2 (-36·1,286·6) |
| **Togo** | 144 (61,309) | 4·8 (2·7,8·9) | 484 (184,1060) | 6·5 (3·2,12·9) | 34·9 (-46·1,193·4) |
