# Supplementary material for: Global, regional, and national trends and burden of multiple sclerosis in adolescents and young adults: a data analysis from 1990 to 2021 and projections to 2040
Source: Front Immunol. 2025 Oct 22;16:1685316. doi: 10.3389/fimmu.2025.1685316 (PMC12586062; doi:10.3389/fimmu.2025.1685316)
Supplement: Supplementary file 1 [file DataSheet1.zip › Table 2 (10).DOCX]

| **Table S2: Prevalent cases of MS in 1990 and 2021 and the percentage change in the age-standardised rates (ASRs) per 100,000, by location (Generated from data available from http://ghdx.healthdata.org/gbd-results-tool)** | | | | | |
| --- | --- | --- | --- | --- | --- |
|  | **1990** | | **2021** | | Percentage change in the ASRs per 100000 |
|  | No (95%UI) | ASRs per 100000 (95%UI) | No (95%UI) | ASRs per 100000 (95%UI) |  |
| **Global** | 324521 (258177,397847) | 22·3 (19·3,25·6) | 489310 (406397,583453) | 22·2 (19·8,24·8) | -0·4 (-3·6,3·3) |
| **Andean Latin America** | 607 (434,828) | 6·2 (5,7·7) | 1587 (1149,2151) | 9·2 (7·5,11) | 46·8 (40·5,54·3) |
| **Bolivia (Plurinational State of)** | 124 (89,168) | 7·7 (6·2,9·3) | 356 (257,479) | 11·5 (9·6,13·5) | 50·1 (41·4,60·5) |
| **Ecuador** | 133 (94,184) | 5·1 (4,6·3) | 351 (256,468) | 7·5 (6·1,9·1) | 47·5 (39·4,57·5) |
| **Peru** | 349 (250,478) | 6·4 (5·1,7·9) | 880 (632,1191) | 9·3 (7·5,11·2) | 45·5 (37·2,55·4) |
| **Australasia** | 2632 (2147,3141) | 40·3 (35·6,45·8) | 5197 (4077,6374) | 59·9 (52,68·8) | 48·7 (36·3,61·9) |
| **Australia** | 2240 (1837,2660) | 40·7 (36·2,46·1) | 4683 (3660,5768) | 64·9 (56·3,74·9) | 59·2 (44·5,74·5) |
| **New Zealand** | 392 (296,491) | 38·1 (31·8,44·3) | 514 (391,641) | 34·7 (29·8,40·3) | -9 (-13·7,-4·9) |
| **Caribbean** | 1009 (751,1320) | 9·3 (7·7,11·2) | 1657 (1259,2096) | 11·7 (9·7,13·8) | 25·1 (18·8,31·3) |
| **Antigua and Barbuda** | 3 (2,3) | 13·1 (10·8,15·4) | 5 (4,7) | 20·8 (18·2,23·9) | 59·1 (47·4,75·8) |
| **Bahamas** | 10 (7,12) | 10·3 (8·3,12·4) | 18 (14,23) | 13·3 (11,15·9) | 29·6 (22·2,38·8) |
| **Barbados** | 11 (8,14) | 13 (10·8,15·4) | 16 (12,21) | 20·6 (17·8,23·8) | 58·1 (45·6,71·4) |
| **Belize** | 4 (3,5) | 7·1 (5·7,8·8) | 13 (9,16) | 8·6 (7,10·3) | 20·2 (13·7,26·8) |
| **Bermuda** | 3 (2,4) | 13·4 (11,16) | 3 (2,3) | 16·7 (13·9,19·9) | 24·2 (17·3,31·7) |
| **Cuba** | 392 (292,514) | 11·1 (9·1,13·2) | 460 (353,575) | 15·2 (12·6,17·9) | 36·6 (26·9,45·9) |
| **Dominica** | 2 (1,2) | 7·4 (5·9,9·1) | 2 (1,2) | 8·4 (6·8,10) | 13·7 (6·4,19·3) |
| **Dominican Republic** | 168 (124,228) | 7·4 (6,9·2) | 329 (247,423) | 9 (7·3,10·7) | 21·2 (13·3,29·5) |
| **Grenada** | 2 (2,3) | 10 (8·2,11·9) | 4 (3,5) | 13·1 (10·9,15·5) | 30·7 (23,39·5) |
| **Guyana** | 14 (10,19) | 5·3 (4·2,6·6) | 16 (12,21) | 6·5 (5·3,7·9) | 22 (15·1,30·3) |
| **Haiti** | 162 (119,211) | 8·1 (6·6,9·7) | 463 (354,591) | 9·6 (7·9,11·5) | 19·4 (12·7,28·5) |
| **Jamaica** | 54 (40,72) | 7·8 (6·2,9·6) | 87 (64,113) | 9 (7·4,10·9) | 16·2 (9·7,23·2) |
| **Puerto Rico** | 109 (80,141) | 10·1 (8·3,12·1) | 119 (91,151) | 14·2 (11·8,16·8) | 40 (30·8,52·2) |
| **Saint Kitts and Nevis** | 2 (1,2) | 13 (10·8,15·4) | 3 (2,4) | 18 (15·3,21·3) | 38·4 (28,48·5) |
| **Saint Lucia** | 3 (2,4) | 7·5 (6,9·1) | 5 (4,7) | 9·1 (7·5,11) | 22·5 (14·9,30·9) |
| **Saint Vincent and the Grenadines** | 2 (2,3) | 7·3 (5·9,8·9) | 3 (2,4) | 8·9 (7·4,10·6) | 21·8 (15·2,29·7) |
| **Suriname** | 6 (5,9) | 5·5 (4·4,6·8) | 12 (9,16) | 6·8 (5·6,8·4) | 24·2 (18·3,32·2) |
| **Trinidad and Tobago** | 26 (19,35) | 6·6 (5·3,8·1) | 40 (30,51) | 8·3 (6·8,10·1) | 27·1 (18·7,33·7) |
| **United States Virgin Islands** | 3 (2,4) | 9·7 (7·9,11·6) | 2 (2,3) | 11·9 (9·7,14·1) | 22 (15·2,29) |
| **Central Asia** | 2769 (2058,3767) | 29·3 (25·8,33·1) | 4173 (3105,5565) | 29·1 (25·8,32·7) | -0·8 (-4·9,4) |
| **Armenia** | 149 (111,203) | 22·7 (19·4,26·2) | 155 (114,204) | 32·3 (28·3,36·6) | 42·3 (30·5,57·9) |
| **Azerbaijan** | 273 (198,368) | 17·1 (14·1,20·2) | 448 (331,590) | 18·9 (16,22·2) | 10·7 (1·8,19·3) |
| **Georgia** | 209 (152,286) | 19 (15·5,22·5) | 139 (101,186) | 24·3 (21,27·9) | 28·1 (16,42·2) |
| **Kazakhstan** | 841 (616,1116) | 52·2 (46·5,58·3) | 1063 (797,1418) | 59·2 (53·6,65·5) | 13·4 (4·5,23·7) |
| **Kyrgyzstan** | 157 (114,214) | 17·4 (14·3,20·5) | 262 (193,349) | 18·3 (15·4,21·4) | 5·3 (-1·7,12·1) |
| **Mongolia** | 77 (55,106) | 17·5 (14·3,21) | 138 (102,185) | 18·3 (15·3,21·7) | 4·8 (-1·6,12·2) |
| **Tajikistan** | 159 (115,214) | 16·1 (13·3,19) | 356 (265,479) | 15·9 (13·3,18·6) | -1·4 (-7·2,5·6) |
| **Turkmenistan** | 132 (98,177) | 29 (25·7,32·4) | 210 (156,285) | 32·2 (28·9,35·8) | 11·2 (5·4,18·4) |
| **Uzbekistan** | 773 (572,1036) | 24·2 (20·8,27·7) | 1403 (1030,1852) | 19·3 (16·5,22·5) | -20 (-25·2,-14·5) |
| **Central Europe** | 16913 (13355,20917) | 38·5 (33·7,43·7) | 16735 (14162,19431) | 42·8 (38·6,47·3) | 11·4 (7,16·9) |
| **Albania** | 397 (299,530) | 88·6 (80·7,97·4) | 431 (318,565) | 84·3 (76·5,93·3) | -4·9 (-11·1,1·9) |
| **Bosnia and Herzegovina** | 486 (373,618) | 27·2 (23·1,32) | 270 (220,324) | 28·2 (25·2,31·5) | 3·8 (-3·4,11·8) |
| **Bulgaria** | 790 (589,1038) | 46·4 (41·3,52·1) | 739 (550,948) | 50·5 (45·6,56) | 8·9 (2·6,15·4) |
| **Croatia** | 368 (282,465) | 20·8 (17·8,24·2) | 402 (307,496) | 32·5 (28·6,36·9) | 56·1 (46·9,68·7) |
| **Czechia** | 1229 (937,1560) | 34·9 (30·3,40) | 1148 (915,1406) | 35·2 (30·9,39·8) | 0·9 (-4·8,7·9) |
| **Hungary** | 1577 (1360,1817) | 44·7 (40·8,48·6) | 1093 (861,1336) | 35·5 (30·9,40·6) | -20·5 (-26·7,-13·7) |
| **Montenegro** | 86 (65,108) | 41·5 (36·4,47·2) | 93 (72,114) | 50·9 (45·7,56·7) | 22·6 (15·5,31·2) |
| **North Macedonia** | 204 (156,261) | 30·8 (26·5,35·8) | 311 (241,385) | 44·4 (38·9,50·1) | 44 (35·4,53·9) |
| **Poland** | 7951 (6250,9844) | 53·3 (45·4,62·1) | 8559 (7491,9659) | 57·5 (52·2,63) | 8 (-0·4,18·6) |
| **Romania** | 1492 (1156,1862) | 16·3 (13·6,19·2) | 1019 (809,1257) | 17·1 (14·5,19·9) | 4·8 (-0·2,9·6) |
| **Serbia** | 1327 (986,1644) | 36·6 (32·1,41·5) | 1641 (1296,1962) | 50·5 (45·5,55·8) | 38 (29·4,48·3) |
| **Slovakia** | 478 (368,598) | 22·2 (18·9,26) | 538 (425,660) | 27·4 (23·9,31·5) | 23·3 (16·3,32·2) |
| **Slovenia** | 257 (195,326) | 37·2 (32·4,42·6) | 247 (191,307) | 44·5 (39·6,50) | 19·4 (13·5,27·1) |
| **Central Latin America** | 3056 (2232,4119) | 6·6 (5·4,8·1) | 7683 (5710,9933) | 10·4 (8·5,12·2) | 56·9 (47·8,66·5) |
| **Colombia** | 454 (319,621) | 4·6 (3·7,5·8) | 973 (713,1294) | 6·7 (5·5,8·1) | 43·9 (35·7,53·1) |
| **Costa Rica** | 58 (42,77) | 7·2 (5·9,8·8) | 141 (101,186) | 10·7 (8·9,12·7) | 47·6 (37·5,59·8) |
| **El Salvador** | 72 (52,98) | 5·1 (4·1,6·4) | 126 (93,167) | 7 (5·7,8·5) | 36·1 (28·7,44·8) |
| **Guatemala** | 111 (78,151) | 5·2 (4·2,6·5) | 360 (264,485) | 7·8 (6·4,9·5) | 49·8 (40·4,59·8) |
| **Honduras** | 65 (46,88) | 5·2 (4·1,6·4) | 220 (161,294) | 7 (5·6,8·4) | 34·1 (27·3,43·1) |
| **Mexico** | 1907 (1409,2556) | 8 (6·5,9·7) | 5027 (3795,6421) | 13·1 (10·9,15·5) | 63·7 (52·8,75·7) |
| **Nicaragua** | 55 (40,75) | 6 (4·8,7·3) | 174 (125,236) | 10 (8·4,12) | 67·7 (55·9,83·2) |
| **Panama** | 35 (26,48) | 5·2 (4·2,6·3) | 81 (59,107) | 6·8 (5·6,8·3) | 32·5 (26·1,39·8) |
| **Venezuela (Bolivarian Republic of)** | 300 (217,413) | 5·4 (4·4,6·7) | 581 (422,765) | 7·7 (6·3,9·2) | 42·7 (32·5,52·6) |
| **Central Sub-Saharan Africa** | 678 (479,937) | 4 (3·1,5) | 1877 (1348,2591) | 4·3 (3·5,5·4) | 8·3 (3·8,13) |
| **Angola** | 155 (111,213) | 4·7 (3·7,5·9) | 505 (369,684) | 5·2 (4·2,6·5) | 11·4 (6·3,17·6) |
| **Central African Republic** | 36 (26,50) | 4·1 (3·2,5·1) | 77 (55,106) | 4·2 (3·3,5·2) | 2·8 (-2·4,7·8) |
| **Congo** | 29 (20,39) | 3·9 (3,4·9) | 79 (57,106) | 4·2 (3·4,5·2) | 9 (3·3,14·7) |
| **Democratic Republic of the Congo** | 442 (310,611) | 3·8 (3,4·8) | 1168 (835,1619) | 4·1 (3·2,5·1) | 6·2 (0·4,11·7) |
| **Equatorial Guinea** | 5 (3,7) | 3·7 (2·9,4·7) | 22 (16,31) | 4·4 (3·5,5·4) | 17·1 (9·6,24·6) |
| **Gabon** | 12 (8,16) | 3·9 (3·1,4·8) | 26 (19,36) | 4·4 (3·5,5·4) | 13·3 (7·5,19·3) |
| **East Asia** | 10128 (6877,14793) | 1·6 (1·2,2·1) | 12706 (9035,17894) | 2·3 (1·8,2·9) | 45·2 (38·7,52·6) |
| **China** | 9592 (6488,14052) | 1·6 (1·2,2) | 11750 (8275,16661) | 2·2 (1·7,2·8) | 43·3 (37,50·2) |
| **Democratic People's Republic of Korea** | 297 (213,413) | 3 (2·3,3·8) | 406 (292,553) | 3·4 (2·7,4·3) | 14·9 (7·8,21) |
| **Taiwan (Province of China)** | 238 (182,313) | 2·4 (2·1,2·9) | 550 (423,683) | 6 (4·9,7·1) | 149·9 (120·7,199·9) |
| **Eastern Europe** | 20412 (15736,25668) | 23·5 (20,27·4) | 21505 (18843,24332) | 27·2 (25,29·9) | 15·8 (6·6,28·1) |
| **Belarus** | 483 (365,622) | 13·3 (11·2,15·9) | 473 (366,609) | 16·1 (14,18·8) | 21·7 (14,31·3) |
| **Estonia** | 97 (74,124) | 16·5 (14·2,19·3) | 77 (58,97) | 17·6 (15·2,20·3) | 6·3 (-0·5,13) |
| **Latvia** | 168 (128,220) | 17·2 (14·8,20·1) | 120 (92,153) | 19·9 (17·3,22·8) | 15·6 (8·4,23·2) |
| **Lithuania** | 222 (168,295) | 16·6 (14·2,19·7) | 156 (118,197) | 19 (16·5,21·7) | 14·4 (7·5,22·2) |
| **Republic of Moldova** | 114 (82,155) | 5·6 (4·4,7·1) | 134 (103,174) | 8·8 (7·1,10·8) | 56·2 (39·6,80·9) |
| **Russian Federation** | 15189 (11753,19125) | 24·3 (20·7,28·4) | 16323 (14660,17941) | 29 (26·8,31·4) | 19·6 (7·3,34·8) |
| **Ukraine** | 4139 (3141,5321) | 25·4 (21·6,29·6) | 4222 (3345,5173) | 26·4 (23·1,30·3) | 3·9 (-3·3,11·4) |
| **Eastern Sub-Saharan Africa** | 2581 (1846,3532) | 4·5 (3·6,5·7) | 6580 (4781,8783) | 4·8 (3·8,5·9) | 5·6 (2·9,8·7) |
| **Burundi** | 69 (49,96) | 4 (3·1,4·9) | 178 (128,243) | 4 (3·1,4·9) | 0 (-4·4,4·9) |
| **Comoros** | 7 (5,9) | 5 (4,6·2) | 14 (10,19) | 5·3 (4·3,6·5) | 6·1 (0·9,11·6) |
| **Djibouti** | 7 (5,9) | 4·9 (3·9,6) | 25 (18,34) | 5·2 (4·3,6·4) | 7·9 (2·4,13) |
| **Eritrea** | 55 (40,75) | 5·3 (4·2,6·5) | 133 (97,174) | 5·7 (4·6,7) | 8·3 (2·5,14) |
| **Ethiopia** | 702 (500,960) | 4·6 (3·6,5·8) | 1704 (1236,2270) | 4·8 (3·8,5·9) | 2·9 (-0·2,6·4) |
| **Kenya** | 257 (184,354) | 4 (3·1,5) | 752 (545,1027) | 4·5 (3·6,5·6) | 13·6 (9·4,18·1) |
| **Madagascar** | 214 (155,289) | 5·8 (4·6,7·2) | 567 (418,745) | 5·9 (4·8,7·2) | 2·9 (-1·8,8) |
| **Malawi** | 149 (106,202) | 4·9 (3·8,6·1) | 332 (242,442) | 5·2 (4·1,6·4) | 6·2 (2·1,11) |
| **Mozambique** | 221 (160,301) | 5·5 (4·3,6·8) | 550 (404,731) | 5·9 (4·8,7·2) | 8 (2·1,14·3) |
| **Rwanda** | 89 (63,121) | 3·9 (3·1,4·9) | 193 (138,265) | 4·2 (3·3,5·2) | 7·1 (1·7,12·5) |
| **Somalia** | 101 (72,142) | 4·1 (3·2,5·2) | 273 (196,370) | 4·1 (3·3,5·1) | 1·5 (-3·5,6) |
| **South Sudan** | 76 (54,106) | 4·2 (3·3,5·3) | 123 (88,168) | 4·4 (3·5,5·4) | 5·5 (0·7,10·7) |
| **Uganda** | 185 (131,259) | 3·7 (2·9,4·7) | 515 (373,704) | 4 (3·1,5) | 7·1 (2·3,12·5) |
| **United Republic of Tanzania** | 329 (236,449) | 4·3 (3·4,5·4) | 853 (624,1146) | 4·6 (3·7,5·8) | 6·7 (1·6,11·5) |
| **Zambia** | 119 (85,162) | 5·1 (4,6·2) | 361 (264,481) | 5·5 (4·4,6·7) | 8·9 (3·5,15·9) |
| **High-income Asia Pacific** | 4716 (3513,6265) | 8·6 (7,10·6) | 4004 (3004,5188) | 9·2 (7·6,11·2) | 6·9 (3·4,10·3) |
| **Brunei Darussalam** | 5 (3,6) | 4·4 (3·5,5·5) | 9 (6,12) | 4·8 (3·9,5·9) | 9·1 (4·3,14·9) |
| **Japan** | 3127 (2325,4159) | 8·6 (7,10·6) | 2582 (1931,3351) | 9·4 (7·7,11·4) | 9 (5·8,12·5) |
| **Republic of Korea** | 1531 (1133,2020) | 8·8 (7·2,11) | 1330 (999,1730) | 9·4 (7·7,11·3) | 5·8 (-0·5,11·2) |
| **Singapore** | 53 (38,73) | 4·3 (3·4,5·5) | 84 (60,115) | 4·5 (3·7,5·7) | 5·3 (1·3,10·2) |
| **High-income North America** | 96565 (77789,114917) | 96·9 (84·3,112·1) | 115154 (103469,126764) | 103·6 (96·4,111·3) | 6·9 (-1·7,16·3) |
| **Canada** | 10875 (10376,11397) | 112·2 (109·4,115·3) | 13322 (12691,13949) | 134·2 (130·8,137·7) | 19·6 (16·8,22·3) |
| **Greenland** | 22 (19,25) | 56·3 (48·3,65) | 17 (15,20) | 60·3 (51·8,69·4) | 7·1 (-2·5,17·7) |
| **United States of America** | 85665 (67261,103614) | 95·2 (81·4,111·7) | 101813 (90508,113191) | 100 (92·1,108·3) | 5 (-4·4,15·5) |
| **North Africa and Middle East** | 33049 (27036,39707) | 34·5 (29·9,39·6) | 91169 (73900,111046) | 45·1 (39,52) | 30·6 (27,34·5) |
| **Afghanistan** | 699 (545,862) | 33·3 (27·5,39·4) | 3977 (3055,4945) | 47·1 (40,55·4) | 41·3 (30·7,51·5) |
| **Algeria** | 2341 (1796,2964) | 33·3 (27·6,40) | 7552 (5867,9482) | 51·1 (43·2,60·6) | 53·5 (42,67·7) |
| **Bahrain** | 56 (42,73) | 25·8 (21,31·2) | 218 (168,277) | 37·3 (30·6,45·3) | 44·6 (35·5,55·7) |
| **Egypt** | 2772 (2106,3546) | 16·7 (13·6,20·1) | 12425 (9707,15719) | 36 (29·9,43·3) | 115·8 (100·9,133·4) |
| **Iran (Islamic Republic of)** | 8070 (6343,9938) | 53·6 (45·2,62·6) | 16359 (12953,19835) | 55·3 (48·3,63·2) | 3·2 (-0·6,7·7) |
| **Iraq** | 1510 (1135,1933) | 29·8 (24·4,35·8) | 5422 (4188,6858) | 42 (34·5,50) | 40·8 (31·5,51·2) |
| **Jordan** | 485 (396,573) | 48·4 (42·4,55) | 2187 (1676,2756) | 54·7 (44·8,65·7) | 13 (2·4,23·9) |
| **Kuwait** | 228 (178,287) | 31·5 (26·1,38) | 1245 (1004,1527) | 55·9 (47,65·9) | 77·2 (65·5,91·6) |
| **Lebanon** | 339 (258,428) | 38·6 (32,46·7) | 1239 (938,1583) | 61·4 (51·2,74·9) | 59 (45·5,72·6) |
| **Libya** | 357 (275,453) | 30·8 (25·2,37·2) | 1265 (968,1583) | 49·3 (41·4,58·7) | 60 (49·4,72·6) |
| **Morocco** | 2502 (1905,3181) | 31·7 (25·8,38) | 5999 (4531,7610) | 49·8 (41·5,59·8) | 57·2 (45·9,69·8) |
| **Oman** | 179 (136,226) | 27·6 (22·9,33·2) | 967 (729,1251) | 46·4 (39·4,55·4) | 68·4 (53·8,84·4) |
| **Palestine** | 177 (136,224) | 34·6 (28·3,41·6) | 794 (609,994) | 49·6 (40·9,59·2) | 43·4 (34,52·9) |
| **Qatar** | 83 (63,108) | 38 (30·8,46·1) | 1085 (929,1246) | 65·5 (58·6,73·6) | 72·5 (51·9,95·3) |
| **Saudi Arabia** | 1127 (852,1473) | 22·1 (18,26·8) | 5621 (4314,7253) | 32·9 (27,40·4) | 48·6 (39,57·5) |
| **Sudan** | 1158 (882,1491) | 20·2 (16·4,24·3) | 4157 (3195,5303) | 29·8 (24·7,36·1) | 47·6 (37·3,58·8) |
| **Syrian Arab Republic** | 1032 (773,1322) | 30·7 (25,37·1) | 1629 (1278,2059) | 45·5 (37·5,54·2) | 48·2 (40·8,59·9) |
| **Tunisia** | 935 (724,1207) | 37 (30·5,44·7) | 2229 (1710,2810) | 58·1 (48·4,69·4) | 56·9 (46·2,69·6) |
| **Turkey** | 8077 (7489,8681) | 47 (44·8,49·4) | 12449 (11482,13374) | 48·3 (45·9,50·5) | 2·7 (0·2,5·6) |
| **United Arab Emirates** | 208 (152,264) | 23 (19,26·9) | 1288 (1031,1582) | 25 (21·9,28·6) | 9·1 (0·8,19·8) |
| **Yemen** | 699 (531,896) | 20 (16·3,24·1) | 2977 (2289,3799) | 28·6 (23·6,34·2) | 42·5 (33·6,53·4) |
| **Oceania** | 46 (31,68) | 1·6 (1·2,2·1) | 101 (68,148) | 1·6 (1·2,2·1) | 0·6 (-2·6,4) |
| **American Samoa** | 0 (0,1) | 1·9 (1·5,2·5) | 0 (0,1) | 2 (1·6,2·6) | 4·8 (0·1,10) |
| **Cook Islands** | 0 (0,0) | 2·4 (1·8,3) | 0 (0,0) | 2·5 (2,3·2) | 6·7 (1·4,11·9) |
| **Fiji** | 7 (5,10) | 2 (1·6,2·6) | 8 (6,12) | 2·1 (1·6,2·7) | 4·2 (-0·6,9·1) |
| **Guam** | 1 (1,2) | 2 (1·5,2·5) | 1 (1,2) | 2 (1·6,2·6) | 3·2 (-1·2,8·4) |
| **Kiribati** | 1 (0,1) | 1·6 (1·2,2) | 1 (1,1) | 1·7 (1·3,2·2) | 11·1 (5·3,17·6) |
| **Marshall Islands** | 0 (0,0) | 1·7 (1·3,2·2) | 0 (0,1) | 1·8 (1·4,2·4) | 7·8 (2·1,14·5) |
| **Micronesia (Federated States of)** | 1 (0,1) | 1·6 (1·2,2·1) | 1 (1,1) | 1·8 (1·4,2·3) | 9·8 (4·3,15·1) |
| **Nauru** | 0 (0,0) | 1·4 (1·1,1·9) | 0 (0,0) | 1·6 (1·2,2) | 8·5 (4·2,14·8) |
| **Niue** | 0 (0,0) | 2·3 (1·8,3) | 0 (0,0) | 2·5 (1·9,3·1) | 4·8 (-0·4,10·6) |
| **Northern Mariana Islands** | 1 (0,1) | 2·2 (1·7,2·8) | 0 (0,1) | 2·3 (1·8,2·9) | 7·2 (2·3,12·3) |
| **Palau** | 0 (0,0) | 1·7 (1·3,2·2) | 0 (0,0) | 1·8 (1·4,2·3) | 5·9 (0·5,10·7) |
| **Papua New Guinea** | 26 (18,39) | 1·4 (1,1·9) | 72 (48,106) | 1·5 (1·1,1·9) | 3 (-2,8·5) |
| **Samoa** | 1 (1,2) | 2 (1·5,2·5) | 2 (1,2) | 2·1 (1·6,2·7) | 5·9 (1·1,10·8) |
| **Solomon Islands** | 2 (2,3) | 2 (1·5,2·5) | 6 (4,8) | 2·2 (1·7,2·8) | 10·9 (5·5,18·5) |
| **Tokelau** | 0 (0,0) | 1·8 (1·4,2·3) | 0 (0,0) | 1·9 (1·5,2·4) | 6·5 (2·2,11) |
| **Tonga** | 1 (1,1) | 2·3 (1·8,2·9) | 1 (1,1) | 2·4 (1·9,3·1) | 6·2 (1·5,11) |
| **Tuvalu** | 0 (0,0) | 1·6 (1·3,2·1) | 0 (0,0) | 1·7 (1·3,2·2) | 5·3 (0·6,11·2) |
| **Vanuatu** | 1 (1,2) | 1·9 (1·4,2·5) | 3 (2,4) | 2 (1·6,2·6) | 6·2 (0·3,11·6) |
| **South Asia** | 25230 (18668,33711) | 7·1 (5·7,8·7) | 56594 (42567,73416) | 8·4 (6·9,10·1) | 18·9 (14·4,23·4) |
| **Bangladesh** | 2331 (1699,3084) | 6·9 (5·5,8·4) | 4619 (3460,6047) | 7·8 (6·4,9·5) | 13·7 (9,18·2) |
| **Bhutan** | 15 (11,20) | 7·4 (6,9) | 26 (20,34) | 8·9 (7·3,10·6) | 19·4 (13·5,25·8) |
| **India** | 19775 (14617,26579) | 6·9 (5·6,8·5) | 42735 (32151,55594) | 8·2 (6·8,9·9) | 19·1 (14·2,23·6) |
| **Nepal** | 460 (343,613) | 7·4 (6,9·2) | 993 (745,1289) | 9 (7·4,10·7) | 20·8 (13·2,28) |
| **Pakistan** | 2649 (1985,3482) | 8·3 (6·8,10·2) | 8221 (6272,10607) | 9·8 (8·1,11·6) | 17·6 (12·2,23·6) |
| **Southeast Asia** | 4437 (3082,6338) | 2·2 (1·7,2·8) | 6919 (4891,9817) | 2·4 (1·9,3) | 10 (6·9,13·2) |
| **Cambodia** | 83 (57,119) | 2 (1·5,2·6) | 177 (122,251) | 2·2 (1·7,2·8) | 12 (6·2,17·4) |
| **Indonesia** | 1568 (1079,2257) | 1·9 (1·5,2·5) | 2542 (1760,3628) | 2·1 (1·6,2·7) | 9·2 (5·7,12·4) |
| **Lao People's Democratic Republic** | 38 (26,54) | 2·2 (1·7,2·9) | 86 (61,121) | 2·4 (1·8,3·1) | 8·8 (3·6,14·1) |
| **Malaysia** | 134 (91,196) | 1·8 (1·4,2·3) | 282 (193,409) | 2 (1·6,2·5) | 11·1 (6·3,17·5) |
| **Maldives** | 1 (1,2) | 1·8 (1·3,2·3) | 6 (4,8) | 1·9 (1·4,2·4) | 5·8 (0·9,10·9) |
| **Mauritius** | 14 (10,19) | 2·6 (2,3·3) | 13 (9,18) | 2·8 (2·2,3·5) | 7·3 (2·5,12·2) |
| **Myanmar** | 465 (327,660) | 2·4 (1·9,3·1) | 652 (462,893) | 2·6 (2·1,3·4) | 9·8 (5·1,14·9) |
| **Philippines** | 622 (434,883) | 2·4 (1·9,3·1) | 1209 (855,1701) | 2·5 (1·9,3·2) | 4 (1·9,6·6) |
| **Seychelles** | 1 (1,1) | 2·4 (1·9,3·1) | 1 (1,2) | 3·1 (2·4,3·8) | 27 (18·2,36·3) |
| **Sri Lanka** | 173 (119,253) | 2·2 (1·7,2·9) | 193 (137,273) | 2·5 (1·9,3·1) | 10·4 (3·6,17·4) |
| **Thailand** | 632 (438,907) | 2·4 (1·8,3) | 578 (408,809) | 2·6 (2,3·3) | 9·3 (4·7,13·9) |
| **Timor-Leste** | 6 (4,9) | 1·8 (1·3,2·3) | 11 (7,16) | 2 (1·5,2·5) | 11·4 (6·5,17·4) |
| **Viet Nam** | 693 (485,988) | 2·5 (1·9,3·2) | 1159 (828,1625) | 2·9 (2·2,3·6) | 16 (9·5,22·8) |
| **Southern Latin America** | 3426 (2647,4322) | 22·7 (18·9,26·8) | 5090 (3905,6337) | 23 (19,27·4) | 1·5 (-3·2,6·1) |
| **Uruguay** | 219 (168,277) | 23·9 (19·7,27·9) | 264 (204,328) | 26·2 (22·1,30·9) | 9·9 (4·3,16·8) |
| **Argentina** | 2342 (1812,2952) | 24·1 (20·1,28·4) | 3591 (2717,4459) | 24·1 (20·1,28·6) | 0·1 (-5·4,5·9) |
| **Chile** | 864 (657,1102) | 18·7 (15,22·4) | 1236 (944,1571) | 20 (16·3,24·2) | 7·4 (2·4,12·7) |
| **Southern Sub-Saharan Africa** | 1140 (824,1524) | 6·8 (5·5,8·4) | 2052 (1518,2750) | 7·3 (6,8·8) | 6·4 (2·7,10·5) |
| **Botswana** | 24 (17,32) | 5·9 (4·7,7·2) | 60 (45,81) | 6·4 (5·2,7·8) | 8·2 (3·8,13·4) |
| **Eswatini** | 15 (11,20) | 6·4 (5·1,7·9) | 29 (21,38) | 6·9 (5·5,8·3) | 6·9 (1·5,13·6) |
| **Lesotho** | 30 (21,40) | 6·7 (5·3,8·3) | 48 (36,64) | 7·1 (5·9,8·6) | 6·5 (0·1,14·2) |
| **Namibia** | 25 (18,33) | 5·8 (4·6,7·1) | 53 (39,71) | 6·3 (5·1,7·8) | 9·5 (4·5,15·5) |
| **South Africa** | 885 (643,1181) | 7·2 (5·9,8·9) | 1564 (1160,2094) | 7·6 (6·3,9·2) | 5·4 (1·6,9·2) |
| **Zimbabwe** | 163 (118,219) | 5·4 (4·3,6·7) | 298 (219,399) | 5·8 (4·7,7) | 7·1 (0·9,13·9) |
| **Tropical Latin America** | 5534 (4055,7424) | 17·1 (14·3,20·2) | 10032 (7317,13313) | 21·2 (18·1,25) | 24·3 (19·1,30·6) |
| **Brazil** | 5410 (3958,7258) | 17·2 (14·4,20·3) | 9749 (7103,12952) | 21·4 (18·2,25·1) | 24·5 (19·3,30·9) |
| **Paraguay** | 125 (92,169) | 13·4 (11·1,16·3) | 284 (213,373) | 15·1 (12·5,18·1) | 12·4 (6·3,19·3) |
| **Western Europe** | 86027 (68624,103003) | 68·1 (59·3,78·4) | 107205 (87700,126641) | 91·4 (80·9,103) | 34·1 (29·5,40) |
| **Andorra** | 14 (11,18) | 61·8 (53·7,71·1) | 20 (16,25) | 83·7 (72·9,97·1) | 35·4 (25·5,45·8) |
| **Austria** | 1530 (1149,1903) | 58·1 (48·9,68·7) | 2108 (1647,2500) | 83·4 (73·1,95·1) | 43·4 (30·7,58·8) |
| **Belgium** | 2154 (1711,2600) | 66 (58·6,74·4) | 2579 (1990,3207) | 83·3 (72·1,97·6) | 26·1 (14·9,36·7) |
| **Cyprus** | 82 (68,98) | 32·3 (28·8,36·8) | 234 (178,289) | 49·2 (41·4,58) | 52·1 (38·8,67·1) |
| **Denmark** | 1701 (1331,2043) | 101·8 (91·5,111·9) | 1801 (1384,2199) | 115·5 (101·4,134·7) | 13·5 (4·2,24·7) |
| **Finland** | 1131 (943,1323) | 65·9 (59·5,72·9) | 1187 (987,1393) | 82·3 (75,90·7) | 24·9 (17·5,33·7) |
| **France** | 13005 (9937,15789) | 62·8 (53·6,74) | 17271 (14505,20258) | 91·8 (81·5,103·1) | 46·2 (33·6,61·4) |
| **Germany** | 19640 (15251,23968) | 71·6 (62·3,84) | 20920 (16271,25539) | 90·5 (79·3,105·1) | 26·3 (14·8,37) |
| **Greece** | 835 (682,1010) | 24·6 (21·3,28·1) | 1048 (823,1274) | 37·6 (32·2,44·2) | 53 (42·9,65·8) |
| **Iceland** | 86 (78,96) | 101·1 (95·2,107·6) | 113 (91,138) | 110·6 (95·6,129·9) | 9·4 (-3,25·6) |
| **Ireland** | 1277 (1021,1562) | 110·1 (97·1,126·2) | 1886 (1432,2336) | 129·8 (111·5,155·6) | 17·9 (6·2,30·8) |
| **Israel** | 489 (386,607) | 29·8 (24·7,35·2) | 1011 (804,1250) | 36 (30·1,42·2) | 21 (13·9,27·6) |
| **Italy** | 12445 (9877,15162) | 65·3 (55·7,76·4) | 13322 (10607,16023) | 86·5 (74·8,101·3) | 32·5 (27,38·3) |
| **Luxembourg** | 104 (79,127) | 74·9 (64·8,87·5) | 189 (149,227) | 90·1 (78·3,103·8) | 20·4 (11·4,32·3) |
| **Malta** | 24 (19,29) | 19 (16·4,22·4) | 34 (27,42) | 26·6 (22·4,31·3) | 40·1 (29·7,51·1) |
| **Monaco** | 4 (3,5) | 45·2 (38·7,53·8) | 5 (4,6) | 56·6 (48·2,65·6) | 25·1 (16·7,33·9) |
| **Netherlands** | 4275 (3179,5277) | 80·3 (69·4,93·2) | 4354 (3366,5309) | 95·9 (83·4,111·2) | 19·3 (10·3,31·4) |
| **Norway** | 1083 (829,1340) | 82·8 (70·7,96·9) | 1817 (1340,2283) | 131·5 (111,154·2) | 58·8 (50·3,70) |
| **Portugal** | 1005 (793,1264) | 30·7 (25·7,36·4) | 808 (688,938) | 31·6 (28·8,34·7) | 2·9 (-7·3,13·4) |
| **San Marino** | 3 (2,4) | 37·5 (30·8,45·3) | 3 (2,4) | 41·3 (34,49·8) | 9·9 (3·9,15·6) |
| **Spain** | 6658 (5607,7751) | 49·7 (44·2,55·7) | 9090 (8100,10258) | 74·5 (67·8,81·2) | 50 (38·7,62·4) |
| **Sweden** | 3066 (2456,3654) | 126 (110·1,144·5) | 4241 (3324,5169) | 161·6 (140·2,187) | 28·3 (22·9,33·9) |
| **Switzerland** | 1948 (1472,2410) | 86·5 (75·8,100·1) | 2365 (1847,2885) | 94·9 (83·7,107·3) | 9·8 (1·1,22·7) |
| **United Kingdom** | 13396 (10400,16538) | 81·5 (70·9,93·4) | 20703 (16358,24964) | 120·1 (104·1,136·6) | 47·3 (41·5,55) |
| **Western Sub-Saharan Africa** | 3566 (2604,4814) | 6·5 (5·3,7·9) | 11288 (8482,14763) | 8·3 (7,9·9) | 28·6 (23·8,35·3) |
| **Benin** | 86 (63,115) | 6·4 (5·2,7·9) | 278 (207,365) | 7 (5·7,8·4) | 8·8 (1·4,14·5) |
| **Burkina Faso** | 164 (121,223) | 6·7 (5·4,8·2) | 488 (362,644) | 7·1 (5·8,8·6) | 6·2 (1·1,12·3) |
| **Cabo Verde** | 7 (5,9) | 7·7 (6·3,9·4) | 18 (14,24) | 8·7 (7·2,10·3) | 13 (5·5,19·2) |
| **Cameroon** | 173 (127,232) | 5·9 (4·8,7·3) | 678 (501,885) | 6·8 (5·5,8·1) | 13·7 (8,21·1) |
| **Chad** | 114 (83,152) | 6·9 (5·6,8·5) | 350 (260,463) | 7·3 (6,9) | 5·9 (0,12·2) |
| **CÃ´te d'Ivoire** | 213 (155,285) | 5·7 (4·6,7) | 583 (428,768) | 6·3 (5·1,7·6) | 10 (5·1,15·4) |
| **Gambia** | 20 (14,26) | 6·6 (5·3,8·1) | 60 (45,79) | 7·9 (6·6,9·6) | 20·1 (13·2,28·1) |
| **Ghana** | 421 (305,556) | 11·4 (9·6,13·4) | 1690 (1221,2208) | 20·6 (18,23·6) | 80·1 (62·7,101·4) |
| **Guinea** | 109 (79,146) | 6·3 (5·1,7·8) | 292 (219,384) | 7 (5·8,8·5) | 11 (5·6,17·6) |
| **Guinea-Bissau** | 20 (15,27) | 6·9 (5·6,8·4) | 53 (40,70) | 7·8 (6·5,9·2) | 13·1 (6·8,21·2) |
| **Liberia** | 42 (31,57) | 5·6 (4·5,6·8) | 113 (83,150) | 6·2 (5·1,7·5) | 12·1 (6·5,18·2) |
| **Mali** | 183 (134,244) | 7·6 (6·2,9·4) | 546 (410,700) | 8·1 (6·8,9·6) | 7·1 (1·2,15) |
| **Mauritania** | 51 (37,67) | 8·7 (7,10·4) | 122 (91,159) | 9·6 (8,11·4) | 10·5 (4·5,17·2) |
| **Niger** | 156 (114,209) | 7·1 (5·7,8·7) | 495 (369,648) | 7·7 (6·3,9·3) | 8·4 (2·8,15) |
| **Nigeria** | 1519 (1102,2058) | 5·7 (4·5,7) | 4739 (3497,6251) | 7·3 (5·9,8·7) | 28·1 (23·2,33·7) |
| **Sao Tome and Principe** | 2 (1,2) | 5 (4,6·3) | 4 (3,5) | 5·9 (4·8,7·1) | 16·5 (9·1,23·5) |
| **Senegal** | 146 (106,194) | 7 (5·6,8·6) | 398 (297,523) | 8·1 (6·7,9·6) | 15·3 (9·8,22·3) |
| **Sierra Leone** | 77 (55,104) | 5·8 (4·7,7·1) | 190 (140,253) | 6·4 (5·2,7·7) | 10·6 (4·1,16·2) |
| **Togo** | 65 (47,88) | 6·3 (5·1,7·7) | 191 (143,249) | 7·1 (5·8,8·4) | 12 (5·4,19·3) |
