# Supplementary material for: Global, regional, and national trends and burden of multiple sclerosis in adolescents and young adults: a data analysis from 1990 to 2021 and projections to 2040
Source: Front Immunol. 2025 Oct 22;16:1685316. doi: 10.3389/fimmu.2025.1685316 (PMC12586062; doi:10.3389/fimmu.2025.1685316)
Supplement: Supplementary file 1 [file DataSheet1.zip › Table 1 (29).DOCX]

| **Table S1: Search terms used for the systematic review of the literature on MS in the Global Burden of Disease 2021 Study** |
| --- |
| (multiple sclerosis[Title/Abstract] AND (prevalence[Title/Abstract] or incidence  [Title/Abstract] or mortality [Title/Abstract] or death [Title/Abstract]) AND "Cross-Sectional  Studies"[MeSH Terms]) ; Humans |
