# Supplementary material for: Global, regional, and national trends and burden of multiple sclerosis in adolescents and young adults: a data analysis from 1990 to 2021 and projections to 2040
Source: Front Immunol. 2025 Oct 22;16:1685316. doi: 10.3389/fimmu.2025.1685316 (PMC12586062; doi:10.3389/fimmu.2025.1685316)
Supplement: Supplementary file 2 [file DataSheet2.docx]

**Supplementary table and figure legends**

**Table S1:** Search terms used for the systematic review of the literature on MS in the Global Burden of Disease 2021 Study

**Table S2:** Prevalent cases of MS in 1990 and 2021 and the percentage change in the age-standardised rates (ASRs) per 100,000, by location (Generated from data available from <http://ghdx.healthdata.org/gbd-results-tool>)

**Table S3:** Deaths due to MS in 1990 and 2021 and the percentage change in the age-standardised rates (ASRs) per 100,000, by location (Generated from data available from <http://ghdx.healthdata.org/gbd-results-tool>)

**Table S4:** DALYs due to MS in 1990 and 2021 and the percentage change in the age-standardised rates (ASRs) per 100,000, by location (Generated from data available from <http://ghdx.healthdata.org/gbd-results-tool>)

**Figure S1:** The age-standardised point prevalence of multiple sclerosis in 2021 for the 21 Global Burden of Disease regions, by sex. (Generated from data available from <http://ghdx.healthdata.org/gbd-results-tool>).

**Figure S2:** The age-standardised death rates of multiple sclerosis in 2021 for the 21 Global Burden of Disease regions, by sex. (Generated from data available from <http://ghdx.healthdata.org/gbd-results-tool>).

**Figure S3:** The age-standardised DALY rates of multiple sclerosis in 2021 for the 21 Global Burden of Disease regions, by sex. DALY=disability adjusted life years. (Generated from data available from <http://ghdx.healthdata.org/gbd-results-tool>).

**Figure S4:** The percentage change in the age-standardised point prevalence of MS from 1990 to 2021 for the 21 Global Burden of Disease regions, by sex. (Generated from data available from <http://ghdx.healthdata.org/gbd-results-tool>).

**Figure S5:** The percentage change in the age-standardised death rates of MS from 1990 to 2021 for the 21 Global Burden of Disease regions, by sex. (Generated from data available from <http://ghdx.healthdata.org/gbd-results-tool>).

**Figure S6:** The percentage change in the age-standardised DALY rates of MS from 1990 to 2021 for the 21 Global Burden of Disease regions, by sex. DALY=disability adjusted life years. (Generated from data available from <http://ghdx.healthdata.org/gbd-results-tool>).

**Figure S7**: Age-standardised DALY rate of MS per 100,000 population in 2021, by country. DALY=disability adjusted life years. (Generated from data available from <http://ghdx.healthdata.org/gbd-results-tool>).

**Figure S8**: Global number of deaths and death rate of MS per 1,000 population, by age and sex, in 2021. (Generated from data available from <http://ghdx.healthdata.org/gbd-results-tool>).

**Figure S9**: Global number of DALYs and DALY rate of MS per 100,000 population, by age and sex, in 2021. (Generated from data available from <http://ghdx.healthdata.org/gbd-results-tool>).

**Figure S10**: Age-standardised DALY rates of MS for 204 countries and territories, by SDI, in 2021; Expected values based on the Socio-demographic Index and disease rates in all locations are shown as the black line. Each point shows the observed agestandardised DALY rate for each country in 2021. DALY=disability adjusted life years. SDI= Sociodemographic Index (Generated from data available from <http://ghdx.healthdata.org/gbd-results-tool>).

**Figure S11:** Percentage of DALYs due to MS attributable to smoking among males for 21 GBD regions in 2021. DALY=disability adjusted life years (Generated from data available from <http://ghdx.healthdata.org/gbd-results-tool>).

**Figure S12:** Percentage of DALYs due to MS attributable to smoking among females for 21 GBD regions in 2021. DALY=disability adjusted life years (Generated from data available from <http://ghdx.healthdata.org/gbd-results-tool>).

| **Table S1: Search terms used for the systematic review of the literature on MS in the Global Burden of Disease 2021 Study** |
| --- |
| (multiple sclerosis[Title/Abstract] AND (prevalence[Title/Abstract] or incidence  [Title/Abstract] or mortality [Title/Abstract] or death [Title/Abstract]) AND "Cross-Sectional  Studies"[MeSH Terms]) ; Humans |

**Table S2**

| **Table S2: Prevalent cases of MS in 1990 and 2021 and the percentage change in the age-standardised rates (ASRs) per 100,000, by location (Generated from data available from http://ghdx.healthdata.org/gbd-results-tool)** | | | | | |
| --- | --- | --- | --- | --- | --- |
|  | **1990** | | **2021** | | Percentage change in the ASRs per 100000 |
|  | No (95%UI) | ASRs per 100000 (95%UI) | No (95%UI) | ASRs per 100000 (95%UI) |  |
| **Global** | 324521 (258177,397847) | 22·3 (19·3,25·6) | 489310 (406397,583453) | 22·2 (19·8,24·8) | -0·4 (-3·6,3·3) |
| **Andean Latin America** | 607 (434,828) | 6·2 (5,7·7) | 1587 (1149,2151) | 9·2 (7·5,11) | 46·8 (40·5,54·3) |
| **Bolivia (Plurinational State of)** | 124 (89,168) | 7·7 (6·2,9·3) | 356 (257,479) | 11·5 (9·6,13·5) | 50·1 (41·4,60·5) |
| **Ecuador** | 133 (94,184) | 5·1 (4,6·3) | 351 (256,468) | 7·5 (6·1,9·1) | 47·5 (39·4,57·5) |
| **Peru** | 349 (250,478) | 6·4 (5·1,7·9) | 880 (632,1191) | 9·3 (7·5,11·2) | 45·5 (37·2,55·4) |
| **Australasia** | 2632 (2147,3141) | 40·3 (35·6,45·8) | 5197 (4077,6374) | 59·9 (52,68·8) | 48·7 (36·3,61·9) |
| **Australia** | 2240 (1837,2660) | 40·7 (36·2,46·1) | 4683 (3660,5768) | 64·9 (56·3,74·9) | 59·2 (44·5,74·5) |
| **New Zealand** | 392 (296,491) | 38·1 (31·8,44·3) | 514 (391,641) | 34·7 (29·8,40·3) | -9 (-13·7,-4·9) |
| **Caribbean** | 1009 (751,1320) | 9·3 (7·7,11·2) | 1657 (1259,2096) | 11·7 (9·7,13·8) | 25·1 (18·8,31·3) |
| **Antigua and Barbuda** | 3 (2,3) | 13·1 (10·8,15·4) | 5 (4,7) | 20·8 (18·2,23·9) | 59·1 (47·4,75·8) |
| **Bahamas** | 10 (7,12) | 10·3 (8·3,12·4) | 18 (14,23) | 13·3 (11,15·9) | 29·6 (22·2,38·8) |
| **Barbados** | 11 (8,14) | 13 (10·8,15·4) | 16 (12,21) | 20·6 (17·8,23·8) | 58·1 (45·6,71·4) |
| **Belize** | 4 (3,5) | 7·1 (5·7,8·8) | 13 (9,16) | 8·6 (7,10·3) | 20·2 (13·7,26·8) |
| **Bermuda** | 3 (2,4) | 13·4 (11,16) | 3 (2,3) | 16·7 (13·9,19·9) | 24·2 (17·3,31·7) |
| **Cuba** | 392 (292,514) | 11·1 (9·1,13·2) | 460 (353,575) | 15·2 (12·6,17·9) | 36·6 (26·9,45·9) |
| **Dominica** | 2 (1,2) | 7·4 (5·9,9·1) | 2 (1,2) | 8·4 (6·8,10) | 13·7 (6·4,19·3) |
| **Dominican Republic** | 168 (124,228) | 7·4 (6,9·2) | 329 (247,423) | 9 (7·3,10·7) | 21·2 (13·3,29·5) |
| **Grenada** | 2 (2,3) | 10 (8·2,11·9) | 4 (3,5) | 13·1 (10·9,15·5) | 30·7 (23,39·5) |
| **Guyana** | 14 (10,19) | 5·3 (4·2,6·6) | 16 (12,21) | 6·5 (5·3,7·9) | 22 (15·1,30·3) |
| **Haiti** | 162 (119,211) | 8·1 (6·6,9·7) | 463 (354,591) | 9·6 (7·9,11·5) | 19·4 (12·7,28·5) |
| **Jamaica** | 54 (40,72) | 7·8 (6·2,9·6) | 87 (64,113) | 9 (7·4,10·9) | 16·2 (9·7,23·2) |
| **Puerto Rico** | 109 (80,141) | 10·1 (8·3,12·1) | 119 (91,151) | 14·2 (11·8,16·8) | 40 (30·8,52·2) |
| **Saint Kitts and Nevis** | 2 (1,2) | 13 (10·8,15·4) | 3 (2,4) | 18 (15·3,21·3) | 38·4 (28,48·5) |
| **Saint Lucia** | 3 (2,4) | 7·5 (6,9·1) | 5 (4,7) | 9·1 (7·5,11) | 22·5 (14·9,30·9) |
| **Saint Vincent and the Grenadines** | 2 (2,3) | 7·3 (5·9,8·9) | 3 (2,4) | 8·9 (7·4,10·6) | 21·8 (15·2,29·7) |
| **Suriname** | 6 (5,9) | 5·5 (4·4,6·8) | 12 (9,16) | 6·8 (5·6,8·4) | 24·2 (18·3,32·2) |
| **Trinidad and Tobago** | 26 (19,35) | 6·6 (5·3,8·1) | 40 (30,51) | 8·3 (6·8,10·1) | 27·1 (18·7,33·7) |
| **United States Virgin Islands** | 3 (2,4) | 9·7 (7·9,11·6) | 2 (2,3) | 11·9 (9·7,14·1) | 22 (15·2,29) |
| **Central Asia** | 2769 (2058,3767) | 29·3 (25·8,33·1) | 4173 (3105,5565) | 29·1 (25·8,32·7) | -0·8 (-4·9,4) |
| **Armenia** | 149 (111,203) | 22·7 (19·4,26·2) | 155 (114,204) | 32·3 (28·3,36·6) | 42·3 (30·5,57·9) |
| **Azerbaijan** | 273 (198,368) | 17·1 (14·1,20·2) | 448 (331,590) | 18·9 (16,22·2) | 10·7 (1·8,19·3) |
| **Georgia** | 209 (152,286) | 19 (15·5,22·5) | 139 (101,186) | 24·3 (21,27·9) | 28·1 (16,42·2) |
| **Kazakhstan** | 841 (616,1116) | 52·2 (46·5,58·3) | 1063 (797,1418) | 59·2 (53·6,65·5) | 13·4 (4·5,23·7) |
| **Kyrgyzstan** | 157 (114,214) | 17·4 (14·3,20·5) | 262 (193,349) | 18·3 (15·4,21·4) | 5·3 (-1·7,12·1) |
| **Mongolia** | 77 (55,106) | 17·5 (14·3,21) | 138 (102,185) | 18·3 (15·3,21·7) | 4·8 (-1·6,12·2) |
| **Tajikistan** | 159 (115,214) | 16·1 (13·3,19) | 356 (265,479) | 15·9 (13·3,18·6) | -1·4 (-7·2,5·6) |
| **Turkmenistan** | 132 (98,177) | 29 (25·7,32·4) | 210 (156,285) | 32·2 (28·9,35·8) | 11·2 (5·4,18·4) |
| **Uzbekistan** | 773 (572,1036) | 24·2 (20·8,27·7) | 1403 (1030,1852) | 19·3 (16·5,22·5) | -20 (-25·2,-14·5) |
| **Central Europe** | 16913 (13355,20917) | 38·5 (33·7,43·7) | 16735 (14162,19431) | 42·8 (38·6,47·3) | 11·4 (7,16·9) |
| **Albania** | 397 (299,530) | 88·6 (80·7,97·4) | 431 (318,565) | 84·3 (76·5,93·3) | -4·9 (-11·1,1·9) |
| **Bosnia and Herzegovina** | 486 (373,618) | 27·2 (23·1,32) | 270 (220,324) | 28·2 (25·2,31·5) | 3·8 (-3·4,11·8) |
| **Bulgaria** | 790 (589,1038) | 46·4 (41·3,52·1) | 739 (550,948) | 50·5 (45·6,56) | 8·9 (2·6,15·4) |
| **Croatia** | 368 (282,465) | 20·8 (17·8,24·2) | 402 (307,496) | 32·5 (28·6,36·9) | 56·1 (46·9,68·7) |
| **Czechia** | 1229 (937,1560) | 34·9 (30·3,40) | 1148 (915,1406) | 35·2 (30·9,39·8) | 0·9 (-4·8,7·9) |
| **Hungary** | 1577 (1360,1817) | 44·7 (40·8,48·6) | 1093 (861,1336) | 35·5 (30·9,40·6) | -20·5 (-26·7,-13·7) |
| **Montenegro** | 86 (65,108) | 41·5 (36·4,47·2) | 93 (72,114) | 50·9 (45·7,56·7) | 22·6 (15·5,31·2) |
| **North Macedonia** | 204 (156,261) | 30·8 (26·5,35·8) | 311 (241,385) | 44·4 (38·9,50·1) | 44 (35·4,53·9) |
| **Poland** | 7951 (6250,9844) | 53·3 (45·4,62·1) | 8559 (7491,9659) | 57·5 (52·2,63) | 8 (-0·4,18·6) |
| **Romania** | 1492 (1156,1862) | 16·3 (13·6,19·2) | 1019 (809,1257) | 17·1 (14·5,19·9) | 4·8 (-0·2,9·6) |
| **Serbia** | 1327 (986,1644) | 36·6 (32·1,41·5) | 1641 (1296,1962) | 50·5 (45·5,55·8) | 38 (29·4,48·3) |
| **Slovakia** | 478 (368,598) | 22·2 (18·9,26) | 538 (425,660) | 27·4 (23·9,31·5) | 23·3 (16·3,32·2) |
| **Slovenia** | 257 (195,326) | 37·2 (32·4,42·6) | 247 (191,307) | 44·5 (39·6,50) | 19·4 (13·5,27·1) |
| **Central Latin America** | 3056 (2232,4119) | 6·6 (5·4,8·1) | 7683 (5710,9933) | 10·4 (8·5,12·2) | 56·9 (47·8,66·5) |
| **Colombia** | 454 (319,621) | 4·6 (3·7,5·8) | 973 (713,1294) | 6·7 (5·5,8·1) | 43·9 (35·7,53·1) |
| **Costa Rica** | 58 (42,77) | 7·2 (5·9,8·8) | 141 (101,186) | 10·7 (8·9,12·7) | 47·6 (37·5,59·8) |
| **El Salvador** | 72 (52,98) | 5·1 (4·1,6·4) | 126 (93,167) | 7 (5·7,8·5) | 36·1 (28·7,44·8) |
| **Guatemala** | 111 (78,151) | 5·2 (4·2,6·5) | 360 (264,485) | 7·8 (6·4,9·5) | 49·8 (40·4,59·8) |
| **Honduras** | 65 (46,88) | 5·2 (4·1,6·4) | 220 (161,294) | 7 (5·6,8·4) | 34·1 (27·3,43·1) |
| **Mexico** | 1907 (1409,2556) | 8 (6·5,9·7) | 5027 (3795,6421) | 13·1 (10·9,15·5) | 63·7 (52·8,75·7) |
| **Nicaragua** | 55 (40,75) | 6 (4·8,7·3) | 174 (125,236) | 10 (8·4,12) | 67·7 (55·9,83·2) |
| **Panama** | 35 (26,48) | 5·2 (4·2,6·3) | 81 (59,107) | 6·8 (5·6,8·3) | 32·5 (26·1,39·8) |
| **Venezuela (Bolivarian Republic of)** | 300 (217,413) | 5·4 (4·4,6·7) | 581 (422,765) | 7·7 (6·3,9·2) | 42·7 (32·5,52·6) |
| **Central Sub-Saharan Africa** | 678 (479,937) | 4 (3·1,5) | 1877 (1348,2591) | 4·3 (3·5,5·4) | 8·3 (3·8,13) |
| **Angola** | 155 (111,213) | 4·7 (3·7,5·9) | 505 (369,684) | 5·2 (4·2,6·5) | 11·4 (6·3,17·6) |
| **Central African Republic** | 36 (26,50) | 4·1 (3·2,5·1) | 77 (55,106) | 4·2 (3·3,5·2) | 2·8 (-2·4,7·8) |
| **Congo** | 29 (20,39) | 3·9 (3,4·9) | 79 (57,106) | 4·2 (3·4,5·2) | 9 (3·3,14·7) |
| **Democratic Republic of the Congo** | 442 (310,611) | 3·8 (3,4·8) | 1168 (835,1619) | 4·1 (3·2,5·1) | 6·2 (0·4,11·7) |
| **Equatorial Guinea** | 5 (3,7) | 3·7 (2·9,4·7) | 22 (16,31) | 4·4 (3·5,5·4) | 17·1 (9·6,24·6) |
| **Gabon** | 12 (8,16) | 3·9 (3·1,4·8) | 26 (19,36) | 4·4 (3·5,5·4) | 13·3 (7·5,19·3) |
| **East Asia** | 10128 (6877,14793) | 1·6 (1·2,2·1) | 12706 (9035,17894) | 2·3 (1·8,2·9) | 45·2 (38·7,52·6) |
| **China** | 9592 (6488,14052) | 1·6 (1·2,2) | 11750 (8275,16661) | 2·2 (1·7,2·8) | 43·3 (37,50·2) |
| **Democratic People's Republic of Korea** | 297 (213,413) | 3 (2·3,3·8) | 406 (292,553) | 3·4 (2·7,4·3) | 14·9 (7·8,21) |
| **Taiwan (Province of China)** | 238 (182,313) | 2·4 (2·1,2·9) | 550 (423,683) | 6 (4·9,7·1) | 149·9 (120·7,199·9) |
| **Eastern Europe** | 20412 (15736,25668) | 23·5 (20,27·4) | 21505 (18843,24332) | 27·2 (25,29·9) | 15·8 (6·6,28·1) |
| **Belarus** | 483 (365,622) | 13·3 (11·2,15·9) | 473 (366,609) | 16·1 (14,18·8) | 21·7 (14,31·3) |
| **Estonia** | 97 (74,124) | 16·5 (14·2,19·3) | 77 (58,97) | 17·6 (15·2,20·3) | 6·3 (-0·5,13) |
| **Latvia** | 168 (128,220) | 17·2 (14·8,20·1) | 120 (92,153) | 19·9 (17·3,22·8) | 15·6 (8·4,23·2) |
| **Lithuania** | 222 (168,295) | 16·6 (14·2,19·7) | 156 (118,197) | 19 (16·5,21·7) | 14·4 (7·5,22·2) |
| **Republic of Moldova** | 114 (82,155) | 5·6 (4·4,7·1) | 134 (103,174) | 8·8 (7·1,10·8) | 56·2 (39·6,80·9) |
| **Russian Federation** | 15189 (11753,19125) | 24·3 (20·7,28·4) | 16323 (14660,17941) | 29 (26·8,31·4) | 19·6 (7·3,34·8) |
| **Ukraine** | 4139 (3141,5321) | 25·4 (21·6,29·6) | 4222 (3345,5173) | 26·4 (23·1,30·3) | 3·9 (-3·3,11·4) |
| **Eastern Sub-Saharan Africa** | 2581 (1846,3532) | 4·5 (3·6,5·7) | 6580 (4781,8783) | 4·8 (3·8,5·9) | 5·6 (2·9,8·7) |
| **Burundi** | 69 (49,96) | 4 (3·1,4·9) | 178 (128,243) | 4 (3·1,4·9) | 0 (-4·4,4·9) |
| **Comoros** | 7 (5,9) | 5 (4,6·2) | 14 (10,19) | 5·3 (4·3,6·5) | 6·1 (0·9,11·6) |
| **Djibouti** | 7 (5,9) | 4·9 (3·9,6) | 25 (18,34) | 5·2 (4·3,6·4) | 7·9 (2·4,13) |
| **Eritrea** | 55 (40,75) | 5·3 (4·2,6·5) | 133 (97,174) | 5·7 (4·6,7) | 8·3 (2·5,14) |
| **Ethiopia** | 702 (500,960) | 4·6 (3·6,5·8) | 1704 (1236,2270) | 4·8 (3·8,5·9) | 2·9 (-0·2,6·4) |
| **Kenya** | 257 (184,354) | 4 (3·1,5) | 752 (545,1027) | 4·5 (3·6,5·6) | 13·6 (9·4,18·1) |
| **Madagascar** | 214 (155,289) | 5·8 (4·6,7·2) | 567 (418,745) | 5·9 (4·8,7·2) | 2·9 (-1·8,8) |
| **Malawi** | 149 (106,202) | 4·9 (3·8,6·1) | 332 (242,442) | 5·2 (4·1,6·4) | 6·2 (2·1,11) |
| **Mozambique** | 221 (160,301) | 5·5 (4·3,6·8) | 550 (404,731) | 5·9 (4·8,7·2) | 8 (2·1,14·3) |
| **Rwanda** | 89 (63,121) | 3·9 (3·1,4·9) | 193 (138,265) | 4·2 (3·3,5·2) | 7·1 (1·7,12·5) |
| **Somalia** | 101 (72,142) | 4·1 (3·2,5·2) | 273 (196,370) | 4·1 (3·3,5·1) | 1·5 (-3·5,6) |
| **South Sudan** | 76 (54,106) | 4·2 (3·3,5·3) | 123 (88,168) | 4·4 (3·5,5·4) | 5·5 (0·7,10·7) |
| **Uganda** | 185 (131,259) | 3·7 (2·9,4·7) | 515 (373,704) | 4 (3·1,5) | 7·1 (2·3,12·5) |
| **United Republic of Tanzania** | 329 (236,449) | 4·3 (3·4,5·4) | 853 (624,1146) | 4·6 (3·7,5·8) | 6·7 (1·6,11·5) |
| **Zambia** | 119 (85,162) | 5·1 (4,6·2) | 361 (264,481) | 5·5 (4·4,6·7) | 8·9 (3·5,15·9) |
| **High-income Asia Pacific** | 4716 (3513,6265) | 8·6 (7,10·6) | 4004 (3004,5188) | 9·2 (7·6,11·2) | 6·9 (3·4,10·3) |
| **Brunei Darussalam** | 5 (3,6) | 4·4 (3·5,5·5) | 9 (6,12) | 4·8 (3·9,5·9) | 9·1 (4·3,14·9) |
| **Japan** | 3127 (2325,4159) | 8·6 (7,10·6) | 2582 (1931,3351) | 9·4 (7·7,11·4) | 9 (5·8,12·5) |
| **Republic of Korea** | 1531 (1133,2020) | 8·8 (7·2,11) | 1330 (999,1730) | 9·4 (7·7,11·3) | 5·8 (-0·5,11·2) |
| **Singapore** | 53 (38,73) | 4·3 (3·4,5·5) | 84 (60,115) | 4·5 (3·7,5·7) | 5·3 (1·3,10·2) |
| **High-income North America** | 96565 (77789,114917) | 96·9 (84·3,112·1) | 115154 (103469,126764) | 103·6 (96·4,111·3) | 6·9 (-1·7,16·3) |
| **Canada** | 10875 (10376,11397) | 112·2 (109·4,115·3) | 13322 (12691,13949) | 134·2 (130·8,137·7) | 19·6 (16·8,22·3) |
| **Greenland** | 22 (19,25) | 56·3 (48·3,65) | 17 (15,20) | 60·3 (51·8,69·4) | 7·1 (-2·5,17·7) |
| **United States of America** | 85665 (67261,103614) | 95·2 (81·4,111·7) | 101813 (90508,113191) | 100 (92·1,108·3) | 5 (-4·4,15·5) |
| **North Africa and Middle East** | 33049 (27036,39707) | 34·5 (29·9,39·6) | 91169 (73900,111046) | 45·1 (39,52) | 30·6 (27,34·5) |
| **Afghanistan** | 699 (545,862) | 33·3 (27·5,39·4) | 3977 (3055,4945) | 47·1 (40,55·4) | 41·3 (30·7,51·5) |
| **Algeria** | 2341 (1796,2964) | 33·3 (27·6,40) | 7552 (5867,9482) | 51·1 (43·2,60·6) | 53·5 (42,67·7) |
| **Bahrain** | 56 (42,73) | 25·8 (21,31·2) | 218 (168,277) | 37·3 (30·6,45·3) | 44·6 (35·5,55·7) |
| **Egypt** | 2772 (2106,3546) | 16·7 (13·6,20·1) | 12425 (9707,15719) | 36 (29·9,43·3) | 115·8 (100·9,133·4) |
| **Iran (Islamic Republic of)** | 8070 (6343,9938) | 53·6 (45·2,62·6) | 16359 (12953,19835) | 55·3 (48·3,63·2) | 3·2 (-0·6,7·7) |
| **Iraq** | 1510 (1135,1933) | 29·8 (24·4,35·8) | 5422 (4188,6858) | 42 (34·5,50) | 40·8 (31·5,51·2) |
| **Jordan** | 485 (396,573) | 48·4 (42·4,55) | 2187 (1676,2756) | 54·7 (44·8,65·7) | 13 (2·4,23·9) |
| **Kuwait** | 228 (178,287) | 31·5 (26·1,38) | 1245 (1004,1527) | 55·9 (47,65·9) | 77·2 (65·5,91·6) |
| **Lebanon** | 339 (258,428) | 38·6 (32,46·7) | 1239 (938,1583) | 61·4 (51·2,74·9) | 59 (45·5,72·6) |
| **Libya** | 357 (275,453) | 30·8 (25·2,37·2) | 1265 (968,1583) | 49·3 (41·4,58·7) | 60 (49·4,72·6) |
| **Morocco** | 2502 (1905,3181) | 31·7 (25·8,38) | 5999 (4531,7610) | 49·8 (41·5,59·8) | 57·2 (45·9,69·8) |
| **Oman** | 179 (136,226) | 27·6 (22·9,33·2) | 967 (729,1251) | 46·4 (39·4,55·4) | 68·4 (53·8,84·4) |
| **Palestine** | 177 (136,224) | 34·6 (28·3,41·6) | 794 (609,994) | 49·6 (40·9,59·2) | 43·4 (34,52·9) |
| **Qatar** | 83 (63,108) | 38 (30·8,46·1) | 1085 (929,1246) | 65·5 (58·6,73·6) | 72·5 (51·9,95·3) |
| **Saudi Arabia** | 1127 (852,1473) | 22·1 (18,26·8) | 5621 (4314,7253) | 32·9 (27,40·4) | 48·6 (39,57·5) |
| **Sudan** | 1158 (882,1491) | 20·2 (16·4,24·3) | 4157 (3195,5303) | 29·8 (24·7,36·1) | 47·6 (37·3,58·8) |
| **Syrian Arab Republic** | 1032 (773,1322) | 30·7 (25,37·1) | 1629 (1278,2059) | 45·5 (37·5,54·2) | 48·2 (40·8,59·9) |
| **Tunisia** | 935 (724,1207) | 37 (30·5,44·7) | 2229 (1710,2810) | 58·1 (48·4,69·4) | 56·9 (46·2,69·6) |
| **Turkey** | 8077 (7489,8681) | 47 (44·8,49·4) | 12449 (11482,13374) | 48·3 (45·9,50·5) | 2·7 (0·2,5·6) |
| **United Arab Emirates** | 208 (152,264) | 23 (19,26·9) | 1288 (1031,1582) | 25 (21·9,28·6) | 9·1 (0·8,19·8) |
| **Yemen** | 699 (531,896) | 20 (16·3,24·1) | 2977 (2289,3799) | 28·6 (23·6,34·2) | 42·5 (33·6,53·4) |
| **Oceania** | 46 (31,68) | 1·6 (1·2,2·1) | 101 (68,148) | 1·6 (1·2,2·1) | 0·6 (-2·6,4) |
| **American Samoa** | 0 (0,1) | 1·9 (1·5,2·5) | 0 (0,1) | 2 (1·6,2·6) | 4·8 (0·1,10) |
| **Cook Islands** | 0 (0,0) | 2·4 (1·8,3) | 0 (0,0) | 2·5 (2,3·2) | 6·7 (1·4,11·9) |
| **Fiji** | 7 (5,10) | 2 (1·6,2·6) | 8 (6,12) | 2·1 (1·6,2·7) | 4·2 (-0·6,9·1) |
| **Guam** | 1 (1,2) | 2 (1·5,2·5) | 1 (1,2) | 2 (1·6,2·6) | 3·2 (-1·2,8·4) |
| **Kiribati** | 1 (0,1) | 1·6 (1·2,2) | 1 (1,1) | 1·7 (1·3,2·2) | 11·1 (5·3,17·6) |
| **Marshall Islands** | 0 (0,0) | 1·7 (1·3,2·2) | 0 (0,1) | 1·8 (1·4,2·4) | 7·8 (2·1,14·5) |
| **Micronesia (Federated States of)** | 1 (0,1) | 1·6 (1·2,2·1) | 1 (1,1) | 1·8 (1·4,2·3) | 9·8 (4·3,15·1) |
| **Nauru** | 0 (0,0) | 1·4 (1·1,1·9) | 0 (0,0) | 1·6 (1·2,2) | 8·5 (4·2,14·8) |
| **Niue** | 0 (0,0) | 2·3 (1·8,3) | 0 (0,0) | 2·5 (1·9,3·1) | 4·8 (-0·4,10·6) |
| **Northern Mariana Islands** | 1 (0,1) | 2·2 (1·7,2·8) | 0 (0,1) | 2·3 (1·8,2·9) | 7·2 (2·3,12·3) |
| **Palau** | 0 (0,0) | 1·7 (1·3,2·2) | 0 (0,0) | 1·8 (1·4,2·3) | 5·9 (0·5,10·7) |
| **Papua New Guinea** | 26 (18,39) | 1·4 (1,1·9) | 72 (48,106) | 1·5 (1·1,1·9) | 3 (-2,8·5) |
| **Samoa** | 1 (1,2) | 2 (1·5,2·5) | 2 (1,2) | 2·1 (1·6,2·7) | 5·9 (1·1,10·8) |
| **Solomon Islands** | 2 (2,3) | 2 (1·5,2·5) | 6 (4,8) | 2·2 (1·7,2·8) | 10·9 (5·5,18·5) |
| **Tokelau** | 0 (0,0) | 1·8 (1·4,2·3) | 0 (0,0) | 1·9 (1·5,2·4) | 6·5 (2·2,11) |
| **Tonga** | 1 (1,1) | 2·3 (1·8,2·9) | 1 (1,1) | 2·4 (1·9,3·1) | 6·2 (1·5,11) |
| **Tuvalu** | 0 (0,0) | 1·6 (1·3,2·1) | 0 (0,0) | 1·7 (1·3,2·2) | 5·3 (0·6,11·2) |
| **Vanuatu** | 1 (1,2) | 1·9 (1·4,2·5) | 3 (2,4) | 2 (1·6,2·6) | 6·2 (0·3,11·6) |
| **South Asia** | 25230 (18668,33711) | 7·1 (5·7,8·7) | 56594 (42567,73416) | 8·4 (6·9,10·1) | 18·9 (14·4,23·4) |
| **Bangladesh** | 2331 (1699,3084) | 6·9 (5·5,8·4) | 4619 (3460,6047) | 7·8 (6·4,9·5) | 13·7 (9,18·2) |
| **Bhutan** | 15 (11,20) | 7·4 (6,9) | 26 (20,34) | 8·9 (7·3,10·6) | 19·4 (13·5,25·8) |
| **India** | 19775 (14617,26579) | 6·9 (5·6,8·5) | 42735 (32151,55594) | 8·2 (6·8,9·9) | 19·1 (14·2,23·6) |
| **Nepal** | 460 (343,613) | 7·4 (6,9·2) | 993 (745,1289) | 9 (7·4,10·7) | 20·8 (13·2,28) |
| **Pakistan** | 2649 (1985,3482) | 8·3 (6·8,10·2) | 8221 (6272,10607) | 9·8 (8·1,11·6) | 17·6 (12·2,23·6) |
| **Southeast Asia** | 4437 (3082,6338) | 2·2 (1·7,2·8) | 6919 (4891,9817) | 2·4 (1·9,3) | 10 (6·9,13·2) |
| **Cambodia** | 83 (57,119) | 2 (1·5,2·6) | 177 (122,251) | 2·2 (1·7,2·8) | 12 (6·2,17·4) |
| **Indonesia** | 1568 (1079,2257) | 1·9 (1·5,2·5) | 2542 (1760,3628) | 2·1 (1·6,2·7) | 9·2 (5·7,12·4) |
| **Lao People's Democratic Republic** | 38 (26,54) | 2·2 (1·7,2·9) | 86 (61,121) | 2·4 (1·8,3·1) | 8·8 (3·6,14·1) |
| **Malaysia** | 134 (91,196) | 1·8 (1·4,2·3) | 282 (193,409) | 2 (1·6,2·5) | 11·1 (6·3,17·5) |
| **Maldives** | 1 (1,2) | 1·8 (1·3,2·3) | 6 (4,8) | 1·9 (1·4,2·4) | 5·8 (0·9,10·9) |
| **Mauritius** | 14 (10,19) | 2·6 (2,3·3) | 13 (9,18) | 2·8 (2·2,3·5) | 7·3 (2·5,12·2) |
| **Myanmar** | 465 (327,660) | 2·4 (1·9,3·1) | 652 (462,893) | 2·6 (2·1,3·4) | 9·8 (5·1,14·9) |
| **Philippines** | 622 (434,883) | 2·4 (1·9,3·1) | 1209 (855,1701) | 2·5 (1·9,3·2) | 4 (1·9,6·6) |
| **Seychelles** | 1 (1,1) | 2·4 (1·9,3·1) | 1 (1,2) | 3·1 (2·4,3·8) | 27 (18·2,36·3) |
| **Sri Lanka** | 173 (119,253) | 2·2 (1·7,2·9) | 193 (137,273) | 2·5 (1·9,3·1) | 10·4 (3·6,17·4) |
| **Thailand** | 632 (438,907) | 2·4 (1·8,3) | 578 (408,809) | 2·6 (2,3·3) | 9·3 (4·7,13·9) |
| **Timor-Leste** | 6 (4,9) | 1·8 (1·3,2·3) | 11 (7,16) | 2 (1·5,2·5) | 11·4 (6·5,17·4) |
| **Viet Nam** | 693 (485,988) | 2·5 (1·9,3·2) | 1159 (828,1625) | 2·9 (2·2,3·6) | 16 (9·5,22·8) |
| **Southern Latin America** | 3426 (2647,4322) | 22·7 (18·9,26·8) | 5090 (3905,6337) | 23 (19,27·4) | 1·5 (-3·2,6·1) |
| **Uruguay** | 219 (168,277) | 23·9 (19·7,27·9) | 264 (204,328) | 26·2 (22·1,30·9) | 9·9 (4·3,16·8) |
| **Argentina** | 2342 (1812,2952) | 24·1 (20·1,28·4) | 3591 (2717,4459) | 24·1 (20·1,28·6) | 0·1 (-5·4,5·9) |
| **Chile** | 864 (657,1102) | 18·7 (15,22·4) | 1236 (944,1571) | 20 (16·3,24·2) | 7·4 (2·4,12·7) |
| **Southern Sub-Saharan Africa** | 1140 (824,1524) | 6·8 (5·5,8·4) | 2052 (1518,2750) | 7·3 (6,8·8) | 6·4 (2·7,10·5) |
| **Botswana** | 24 (17,32) | 5·9 (4·7,7·2) | 60 (45,81) | 6·4 (5·2,7·8) | 8·2 (3·8,13·4) |
| **Eswatini** | 15 (11,20) | 6·4 (5·1,7·9) | 29 (21,38) | 6·9 (5·5,8·3) | 6·9 (1·5,13·6) |
| **Lesotho** | 30 (21,40) | 6·7 (5·3,8·3) | 48 (36,64) | 7·1 (5·9,8·6) | 6·5 (0·1,14·2) |
| **Namibia** | 25 (18,33) | 5·8 (4·6,7·1) | 53 (39,71) | 6·3 (5·1,7·8) | 9·5 (4·5,15·5) |
| **South Africa** | 885 (643,1181) | 7·2 (5·9,8·9) | 1564 (1160,2094) | 7·6 (6·3,9·2) | 5·4 (1·6,9·2) |
| **Zimbabwe** | 163 (118,219) | 5·4 (4·3,6·7) | 298 (219,399) | 5·8 (4·7,7) | 7·1 (0·9,13·9) |
| **Tropical Latin America** | 5534 (4055,7424) | 17·1 (14·3,20·2) | 10032 (7317,13313) | 21·2 (18·1,25) | 24·3 (19·1,30·6) |
| **Brazil** | 5410 (3958,7258) | 17·2 (14·4,20·3) | 9749 (7103,12952) | 21·4 (18·2,25·1) | 24·5 (19·3,30·9) |
| **Paraguay** | 125 (92,169) | 13·4 (11·1,16·3) | 284 (213,373) | 15·1 (12·5,18·1) | 12·4 (6·3,19·3) |
| **Western Europe** | 86027 (68624,103003) | 68·1 (59·3,78·4) | 107205 (87700,126641) | 91·4 (80·9,103) | 34·1 (29·5,40) |
| **Andorra** | 14 (11,18) | 61·8 (53·7,71·1) | 20 (16,25) | 83·7 (72·9,97·1) | 35·4 (25·5,45·8) |
| **Austria** | 1530 (1149,1903) | 58·1 (48·9,68·7) | 2108 (1647,2500) | 83·4 (73·1,95·1) | 43·4 (30·7,58·8) |
| **Belgium** | 2154 (1711,2600) | 66 (58·6,74·4) | 2579 (1990,3207) | 83·3 (72·1,97·6) | 26·1 (14·9,36·7) |
| **Cyprus** | 82 (68,98) | 32·3 (28·8,36·8) | 234 (178,289) | 49·2 (41·4,58) | 52·1 (38·8,67·1) |
| **Denmark** | 1701 (1331,2043) | 101·8 (91·5,111·9) | 1801 (1384,2199) | 115·5 (101·4,134·7) | 13·5 (4·2,24·7) |
| **Finland** | 1131 (943,1323) | 65·9 (59·5,72·9) | 1187 (987,1393) | 82·3 (75,90·7) | 24·9 (17·5,33·7) |
| **France** | 13005 (9937,15789) | 62·8 (53·6,74) | 17271 (14505,20258) | 91·8 (81·5,103·1) | 46·2 (33·6,61·4) |
| **Germany** | 19640 (15251,23968) | 71·6 (62·3,84) | 20920 (16271,25539) | 90·5 (79·3,105·1) | 26·3 (14·8,37) |
| **Greece** | 835 (682,1010) | 24·6 (21·3,28·1) | 1048 (823,1274) | 37·6 (32·2,44·2) | 53 (42·9,65·8) |
| **Iceland** | 86 (78,96) | 101·1 (95·2,107·6) | 113 (91,138) | 110·6 (95·6,129·9) | 9·4 (-3,25·6) |
| **Ireland** | 1277 (1021,1562) | 110·1 (97·1,126·2) | 1886 (1432,2336) | 129·8 (111·5,155·6) | 17·9 (6·2,30·8) |
| **Israel** | 489 (386,607) | 29·8 (24·7,35·2) | 1011 (804,1250) | 36 (30·1,42·2) | 21 (13·9,27·6) |
| **Italy** | 12445 (9877,15162) | 65·3 (55·7,76·4) | 13322 (10607,16023) | 86·5 (74·8,101·3) | 32·5 (27,38·3) |
| **Luxembourg** | 104 (79,127) | 74·9 (64·8,87·5) | 189 (149,227) | 90·1 (78·3,103·8) | 20·4 (11·4,32·3) |
| **Malta** | 24 (19,29) | 19 (16·4,22·4) | 34 (27,42) | 26·6 (22·4,31·3) | 40·1 (29·7,51·1) |
| **Monaco** | 4 (3,5) | 45·2 (38·7,53·8) | 5 (4,6) | 56·6 (48·2,65·6) | 25·1 (16·7,33·9) |
| **Netherlands** | 4275 (3179,5277) | 80·3 (69·4,93·2) | 4354 (3366,5309) | 95·9 (83·4,111·2) | 19·3 (10·3,31·4) |
| **Norway** | 1083 (829,1340) | 82·8 (70·7,96·9) | 1817 (1340,2283) | 131·5 (111,154·2) | 58·8 (50·3,70) |
| **Portugal** | 1005 (793,1264) | 30·7 (25·7,36·4) | 808 (688,938) | 31·6 (28·8,34·7) | 2·9 (-7·3,13·4) |
| **San Marino** | 3 (2,4) | 37·5 (30·8,45·3) | 3 (2,4) | 41·3 (34,49·8) | 9·9 (3·9,15·6) |
| **Spain** | 6658 (5607,7751) | 49·7 (44·2,55·7) | 9090 (8100,10258) | 74·5 (67·8,81·2) | 50 (38·7,62·4) |
| **Sweden** | 3066 (2456,3654) | 126 (110·1,144·5) | 4241 (3324,5169) | 161·6 (140·2,187) | 28·3 (22·9,33·9) |
| **Switzerland** | 1948 (1472,2410) | 86·5 (75·8,100·1) | 2365 (1847,2885) | 94·9 (83·7,107·3) | 9·8 (1·1,22·7) |
| **United Kingdom** | 13396 (10400,16538) | 81·5 (70·9,93·4) | 20703 (16358,24964) | 120·1 (104·1,136·6) | 47·3 (41·5,55) |
| **Western Sub-Saharan Africa** | 3566 (2604,4814) | 6·5 (5·3,7·9) | 11288 (8482,14763) | 8·3 (7,9·9) | 28·6 (23·8,35·3) |
| **Benin** | 86 (63,115) | 6·4 (5·2,7·9) | 278 (207,365) | 7 (5·7,8·4) | 8·8 (1·4,14·5) |
| **Burkina Faso** | 164 (121,223) | 6·7 (5·4,8·2) | 488 (362,644) | 7·1 (5·8,8·6) | 6·2 (1·1,12·3) |
| **Cabo Verde** | 7 (5,9) | 7·7 (6·3,9·4) | 18 (14,24) | 8·7 (7·2,10·3) | 13 (5·5,19·2) |
| **Cameroon** | 173 (127,232) | 5·9 (4·8,7·3) | 678 (501,885) | 6·8 (5·5,8·1) | 13·7 (8,21·1) |
| **Chad** | 114 (83,152) | 6·9 (5·6,8·5) | 350 (260,463) | 7·3 (6,9) | 5·9 (0,12·2) |
| **CÃ´te d'Ivoire** | 213 (155,285) | 5·7 (4·6,7) | 583 (428,768) | 6·3 (5·1,7·6) | 10 (5·1,15·4) |
| **Gambia** | 20 (14,26) | 6·6 (5·3,8·1) | 60 (45,79) | 7·9 (6·6,9·6) | 20·1 (13·2,28·1) |
| **Ghana** | 421 (305,556) | 11·4 (9·6,13·4) | 1690 (1221,2208) | 20·6 (18,23·6) | 80·1 (62·7,101·4) |
| **Guinea** | 109 (79,146) | 6·3 (5·1,7·8) | 292 (219,384) | 7 (5·8,8·5) | 11 (5·6,17·6) |
| **Guinea-Bissau** | 20 (15,27) | 6·9 (5·6,8·4) | 53 (40,70) | 7·8 (6·5,9·2) | 13·1 (6·8,21·2) |
| **Liberia** | 42 (31,57) | 5·6 (4·5,6·8) | 113 (83,150) | 6·2 (5·1,7·5) | 12·1 (6·5,18·2) |
| **Mali** | 183 (134,244) | 7·6 (6·2,9·4) | 546 (410,700) | 8·1 (6·8,9·6) | 7·1 (1·2,15) |
| **Mauritania** | 51 (37,67) | 8·7 (7,10·4) | 122 (91,159) | 9·6 (8,11·4) | 10·5 (4·5,17·2) |
| **Niger** | 156 (114,209) | 7·1 (5·7,8·7) | 495 (369,648) | 7·7 (6·3,9·3) | 8·4 (2·8,15) |
| **Nigeria** | 1519 (1102,2058) | 5·7 (4·5,7) | 4739 (3497,6251) | 7·3 (5·9,8·7) | 28·1 (23·2,33·7) |
| **Sao Tome and Principe** | 2 (1,2) | 5 (4,6·3) | 4 (3,5) | 5·9 (4·8,7·1) | 16·5 (9·1,23·5) |
| **Senegal** | 146 (106,194) | 7 (5·6,8·6) | 398 (297,523) | 8·1 (6·7,9·6) | 15·3 (9·8,22·3) |
| **Sierra Leone** | 77 (55,104) | 5·8 (4·7,7·1) | 190 (140,253) | 6·4 (5·2,7·7) | 10·6 (4·1,16·2) |
| **Togo** | 65 (47,88) | 6·3 (5·1,7·7) | 191 (143,249) | 7·1 (5·8,8·4) | 12 (5·4,19·3) |

**Table S3**

| **Table S3: Deaths due to MS in 1990 and 2021 and the percentage change in the age-standardised rates (ASRs) per 100,000, by location (Generated from data available from [http://ghdx·healthdata·org/gbd-results-tool](http://ghdx.healthdata.org/gbd-results-tool))** | | | | | |
| --- | --- | --- | --- | --- | --- |
|  | **1990** | | **2021** | | Percentage change in the ASRs per 100000 |
|  | No (95%UI) | ASRs per 100000 (95%UI) | No (95%UI) | ASRs per 100000 (95%UI) |  |
| **Global** | 1276 (1185,1362) | 0·2 (0·2,0·2) | 1424 (1253,1607) | 0·2 (0·2,0·2) | -12·8 (-17·5,-8·3) |
| **Andean Latin America** | 2 (2,3) | 0·1 (0,0·1) | 7 (5,10) | 0·1 (0·1,0·1) | 84 (38·7,141·9) |
| **Bolivia (Plurinational State of)** | 0 (0,1) | 0·1 (0,0·1) | 2 (1,3) | 0·1 (0·1,0·2) | 73·3 (-2·6,235·8) |
| **Ecuador** | 1 (1,1) | 0·1 (0·1,0·1) | 3 (2,3) | 0·1 (0·1,0·2) | 66·3 (29·6,115·6) |
| **Peru** | 1 (1,1) | 0 (0,0·1) | 3 (2,5) | 0·1 (0·1,0·1) | 101·1 (32·6,199·5) |
| **Australasia** | 8 (7,8) | 0·4 (0·4,0·5) | 11 (9,12) | 0·4 (0·4,0·5) | 5·5 (-10·9,21·6) |
| **Australia** | 6 (6,7) | 0·4 (0·4,0·4) | 9 (8,10) | 0·4 (0·4,0·5) | 8·5 (-9·3,26·4) |
| **New Zealand** | 1 (1,1) | 0·5 (0·5,0·6) | 2 (1,2) | 0·5 (0·4,0·6) | -5·6 (-22,14) |
| **Caribbean** | 12 (11,13) | 0·2 (0·2,0·2) | 15 (12,20) | 0·2 (0·2,0·2) | 21·9 (5·1,40·7) |
| **Antigua and Barbuda** | 0 (0,0) | 0·2 (0·2,0·3) | 0 (0,0) | 0·3 (0·3,0·3) | 32 (11,57·4) |
| **Bahamas** | 0 (0,0) | 0·3 (0·3,0·3) | 0 (0,1) | 0·4 (0·3,0·5) | 45·5 (8·6,89·9) |
| **Barbados** | 0 (0,0) | 0·3 (0·3,0·4) | 0 (0,0) | 0·4 (0·3,0·6) | 39·8 (4·8,82·2) |
| **Belize** | 0 (0,0) | 0·1 (0,0·1) | 0 (0,0) | 0·1 (0·1,0·1) | 100·9 (67·2,138·3) |
| **Bermuda** | 0 (0,0) | 0·3 (0·2,0·3) | 0 (0,0) | 0·2 (0·2,0·3) | -19·1 (-35·3,0·7) |
| **Cuba** | 6 (6,7) | 0·2 (0·2,0·3) | 5 (4,5) | 0·3 (0·3,0·4) | 33·3 (11·7,57) |
| **Dominica** | 0 (0,0) | 0·1 (0·1,0·1) | 0 (0,0) | 0·1 (0·1,0·2) | 59·5 (9·6,130·2) |
| **Dominican Republic** | 1 (1,1) | 0 (0,0·1) | 2 (1,3) | 0·1 (0,0·1) | 42·1 (-12·3,158·7) |
| **Grenada** | 0 (0,0) | 0·3 (0·2,0·3) | 0 (0,0) | 0·3 (0·3,0·4) | 22·3 (-0·7,50·5) |
| **Guyana** | 0 (0,0) | 0·1 (0·1,0·1) | 0 (0,0) | 0·1 (0·1,0·1) | 67·8 (13·3,136·1) |
| **Haiti** | 1 (1,2) | 0·1 (0,0·2) | 4 (2,8) | 0·1 (0·1,0·3) | 43·3 (-20·2,155·6) |
| **Jamaica** | 0 (0,0) | 0·1 (0·1,0·1) | 1 (1,1) | 0·2 (0·1,0·2) | 91·3 (41·5,149·5) |
| **Puerto Rico** | 2 (2,2) | 0·3 (0·3,0·3) | 2 (1,2) | 0·3 (0·2,0·3) | 4·9 (-16·1,28·2) |
| **Saint Kitts and Nevis** | 0 (0,0) | 0·5 (0·4,0·5) | 0 (0,0) | 0·4 (0·3,0·5) | -6·8 (-25·5,14·7) |
| **Saint Lucia** | 0 (0,0) | 0·2 (0·2,0·2) | 0 (0,0) | 0·2 (0·2,0·2) | 17·6 (-6·5,44·9) |
| **Saint Vincent and the Grenadines** | 0 (0,0) | 0·1 (0·1,0·1) | 0 (0,0) | 0·1 (0·1,0·1) | 35·1 (8·5,68·5) |
| **Suriname** | 0 (0,0) | 0·1 (0·1,0·1) | 0 (0,0) | 0·1 (0·1,0·2) | 41·3 (-9·4,112·6) |
| **Trinidad and Tobago** | 0 (0,0) | 0·1 (0·1,0·1) | 1 (0,1) | 0·2 (0·1,0·2) | 43·2 (4·5,89·7) |
| **United States Virgin Islands** | 0 (0,0) | 0·2 (0·1,0·2) | 0 (0,0) | 0·2 (0·1,0·2) | -2·1 (-40·1,54·1) |
| **Central Asia** | 16 (14,18) | 0·2 (0·2,0·3) | 12 (9,15) | 0·1 (0·1,0·2) | -35·6 (-50·9,-20·1) |
| **Armenia** | 0 (0,1) | 0·2 (0·1,0·2) | 0 (0,0) | 0·2 (0·2,0·2) | 2 (-20,29·3) |
| **Azerbaijan** | 1 (0,1) | 0·1 (0·1,0·1) | 1 (0,1) | 0·1 (0,0·1) | -21·6 (-62·4,68·5) |
| **Georgia** | 0 (0,1) | 0·1 (0·1,0·1) | 0 (0,0) | 0·1 (0·1,0·1) | 16·8 (-2·2,43·3) |
| **Kazakhstan** | 10 (9,12) | 0·5 (0·4,0·6) | 5 (3,7) | 0·3 (0·2,0·3) | -50·2 (-68·4,-31·2) |
| **Kyrgyzstan** | 1 (0,1) | 0·1 (0·1,0·1) | 1 (0,1) | 0·1 (0·1,0·1) | -12·6 (-32,11·4) |
| **Mongolia** | 0 (0,1) | 0·3 (0·1,0·6) | 1 (0,2) | 0·3 (0·2,0·6) | 20 (-56·9,265·8) |
| **Tajikistan** | 0 (0,0) | 0·1 (0,0·1) | 1 (0,1) | 0·1 (0,0·1) | -11·2 (-54·3,52·8) |
| **Turkmenistan** | 1 (1,2) | 0·4 (0·3,0·5) | 1 (1,2) | 0·3 (0·2,0·4) | -28·3 (-58·2,5·8) |
| **Uzbekistan** | 2 (1,2) | 0·1 (0·1,0·1) | 3 (2,3) | 0·1 (0·1,0·1) | -10·7 (-33·9,20·6) |
| **Central Europe** | 202 (192,215) | 0·9 (0·8,1) | 65 (58,74) | 0·6 (0·5,0·6) | -38·1 (-45,-31·2) |
| **Albania** | 8 (5,12) | 1·6 (1·1,2·1) | 4 (2,7) | 1·1 (0·6,1·8) | -30·6 (-64·5,28·2) |
| **Bosnia and Herzegovina** | 4 (3,5) | 0·5 (0·4,0·7) | 1 (1,2) | 0·3 (0·2,0·5) | -37·9 (-63·4,10·9) |
| **Bulgaria** | 11 (10,12) | 0·8 (0·7,0·8) | 5 (4,7) | 0·6 (0·5,0·8) | -16·1 (-34,5·3) |
| **Croatia** | 5 (5,6) | 0·7 (0·7,0·8) | 2 (1,2) | 0·5 (0·4,0·5) | -38·5 (-49·5,-26·7) |
| **Czechia** | 15 (14,17) | 1·1 (1,1·2) | 6 (5,7) | 0·6 (0·5,0·7) | -49 (-58,-38·9) |
| **Hungary** | 15 (13,18) | 0·8 (0·8,0·9) | 4 (4,5) | 0·5 (0·4,0·6) | -38·6 (-48·1,-26·6) |
| **Montenegro** | 1 (0,1) | 0·7 (0·4,1) | 1 (0,1) | 0·7 (0·5,0·9) | -1·2 (-40·3,63) |
| **North Macedonia** | 2 (1,2) | 0·6 (0·4,0·8) | 1 (1,2) | 0·5 (0·3,0·7) | -14·7 (-46·4,37) |
| **Poland** | 89 (85,93) | 1·2 (1·2,1·3) | 23 (21,26) | 0·6 (0·6,0·7) | -46·8 (-52·8,-40) |
| **Romania** | 27 (24,30) | 0·6 (0·5,0·6) | 6 (5,7) | 0·3 (0·3,0·4) | -46·6 (-55·4,-35·2) |
| **Serbia** | 12 (8,20) | 0·8 (0·5,1·2) | 7 (4,11) | 0·7 (0·5,1) | -7·1 (-45·3,53·2) |
| **Slovakia** | 5 (3,7) | 0·6 (0·4,0·8) | 3 (2,5) | 0·5 (0·3,0·7) | -16·9 (-46·2,31·3) |
| **Slovenia** | 4 (3,4) | 1·1 (1·1,1·2) | 1 (1,1) | 0·5 (0·4,0·7) | -53·1 (-61·5,-42·4) |
| **Central Latin America** | 29 (27,30) | 0·1 (0·1,0·1) | 94 (84,106) | 0·2 (0·2,0·2) | 110·9 (84·7,140·1) |
| **Colombia** | 6 (5,6) | 0·1 (0·1,0·1) | 13 (11,15) | 0·1 (0·1,0·2) | 46·6 (18·9,78·3) |
| **Costa Rica** | 0 (0,1) | 0·1 (0·1,0·1) | 2 (2,2) | 0·2 (0·2,0·3) | 106 (75·6,138·9) |
| **El Salvador** | 0 (0,0) | 0 (0,0·1) | 1 (1,2) | 0·1 (0·1,0·1) | 128·5 (57·5,237·7) |
| **Guatemala** | 1 (1,1) | 0·1 (0·1,0·1) | 3 (3,4) | 0·1 (0·1,0·1) | 60·6 (31·7,92) |
| **Honduras** | 0 (0,0) | 0 (0,0) | 0 (0,1) | 0 (0,0) | 45·8 (-24·9,202·8) |
| **Mexico** | 17 (16,17) | 0·1 (0·1,0·1) | 60 (53,68) | 0·3 (0·2,0·3) | 134·5 (99·6,170·7) |
| **Nicaragua** | 0 (0,0) | 0·1 (0,0·1) | 1 (1,2) | 0·1 (0·1,0·1) | 88·7 (31·9,173·1) |
| **Panama** | 0 (0,0) | 0·1 (0·1,0·1) | 1 (1,1) | 0·1 (0·1,0·2) | 112·4 (62·1,167·4) |
| **Venezuela (Bolivarian Republic of)** | 3 (3,4) | 0·1 (0·1,0·1) | 13 (10,17) | 0·3 (0·2,0·3) | 121·3 (67·4,189·4) |
| **Central Sub-Saharan Africa** | 1 (0,1) | 0 (0,0) | 3 (2,5) | 0 (0,0) | 62·2 (4·5,159·6) |
| **Angola** | 0 (0,0) | 0 (0,0) | 1 (0,2) | 0 (0,0) | 93·1 (12,334·5) |
| **Central African Republic** | 0 (0,0) | 0 (0,0) | 0 (0,0) | 0 (0,0) | 31·3 (-13·6,123·8) |
| **Congo** | 0 (0,0) | 0 (0,0) | 0 (0,0) | 0 (0,0) | 52·5 (-12·3,147·6) |
| **Democratic Republic of the Congo** | 0 (0,1) | 0 (0,0) | 2 (1,3) | 0 (0,0) | 53 (-5·6,162·7) |
| **Equatorial Guinea** | 0 (0,0) | 0 (0,0) | 0 (0,0) | 0 (0,0) | 139·4 (-0·9,608·8) |
| **Gabon** | 0 (0,0) | 0 (0,0) | 0 (0,0) | 0 (0,0) | 59·3 (-16·3,242·7) |
| **East Asia** | 14 (9,22) | 0 (0,0) | 17 (13,22) | 0 (0,0) | 13·3 (-30·6,92) |
| **China** | 14 (8,21) | 0 (0,0) | 16 (12,21) | 0 (0,0) | 11·7 (-33·4,94·5) |
| **Democratic People's Republic of Korea** | 0 (0,1) | 0 (0,0) | 0 (0,1) | 0 (0,0) | 30·4 (-17·4,134·8) |
| **Taiwan (Province of China)** | 0 (0,0) | 0 (0,0) | 1 (0,1) | 0 (0,0) | 69·1 (43·9,94·5) |
| **Eastern Europe** | 358 (342,375) | 0·5 (0·5,0·6) | 142 (123,163) | 0·3 (0·3,0·4) | -37·9 (-45·3,-29·7) |
| **Belarus** | 11 (10,13) | 0·4 (0·4,0·5) | 6 (5,8) | 0·3 (0·2,0·4) | -27·1 (-44·4,-7·4) |
| **Estonia** | 4 (3,4) | 1 (0·9,1·1) | 1 (1,1) | 0·4 (0·3,0·4) | -63·4 (-70·4,-55·7) |
| **Latvia** | 7 (6,8) | 1·2 (1·1,1·3) | 2 (1,2) | 0·6 (0·5,0·7) | -50·3 (-59·5,-39·9) |
| **Lithuania** | 9 (8,10) | 1·1 (1,1·2) | 2 (2,3) | 0·6 (0·5,0·7) | -46 (-56·1,-35) |
| **Republic of Moldova** | 2 (2,3) | 0·2 (0·2,0·2) | 1 (1,1) | 0·1 (0·1,0·1) | -46·4 (-56·4,-36) |
| **Russian Federation** | 219 (210,232) | 0·5 (0·5,0·5) | 83 (75,90) | 0·3 (0·3,0·3) | -36·5 (-43·6,-28·8) |
| **Ukraine** | 105 (95,118) | 0·6 (0·6,0·7) | 47 (32,65) | 0·4 (0·3,0·5) | -36·7 (-55·8,-14·5) |
| **Eastern Sub-Saharan Africa** | 3 (1,5) | 0 (0,0) | 12 (4,18) | 0 (0,0) | 65·9 (18·2,178·2) |
| **Burundi** | 0 (0,0) | 0 (0,0) | 0 (0,0) | 0 (0,0) | 22·7 (-33,93·1) |
| **Comoros** | 0 (0,0) | 0 (0,0) | 0 (0,0) | 0 (0,0) | 78·2 (5·7,349·9) |
| **Djibouti** | 0 (0,0) | 0 (0,0) | 0 (0,0) | 0 (0,0) | 67·1 (-2·3,210) |
| **Eritrea** | 0 (0,0) | 0 (0,0) | 0 (0,1) | 0 (0,0·1) | 86·1 (13·9,248·5) |
| **Ethiopia** | 1 (0,1) | 0 (0,0) | 3 (1,5) | 0 (0,0) | 47·3 (-11·4,119·8) |
| **Kenya** | 0 (0,0) | 0 (0,0) | 1 (1,2) | 0 (0,0) | 98·4 (32·9,344·8) |
| **Madagascar** | 0 (0,0) | 0 (0,0) | 1 (0,2) | 0 (0,0) | 41·8 (-10·8,143·1) |
| **Malawi** | 0 (0,0) | 0 (0,0) | 1 (0,1) | 0 (0,0) | 92·1 (25·2,259·8) |
| **Mozambique** | 0 (0,0) | 0 (0,0) | 1 (0,2) | 0 (0,0) | 92·7 (22·3,325·1) |
| **Rwanda** | 0 (0,0) | 0 (0,0) | 0 (0,1) | 0 (0,0) | 37·9 (-22·9,119·6) |
| **Somalia** | 0 (0,0) | 0 (0,0) | 0 (0,1) | 0 (0,0) | 24·4 (-29·2,90·3) |
| **South Sudan** | 0 (0,0) | 0 (0,0) | 0 (0,0) | 0 (0,0) | 65·5 (0·9,196·7) |
| **Uganda** | 0 (0,0) | 0 (0,0) | 1 (0,2) | 0 (0,0) | 124·9 (34·5,537·7) |
| **United Republic of Tanzania** | 0 (0,1) | 0 (0,0) | 2 (1,3) | 0 (0,0) | 62·5 (-4,239·3) |
| **Zambia** | 0 (0,0) | 0 (0,0) | 1 (0,2) | 0 (0,0) | 76·4 (-5·8,303) |
| **High-income Asia Pacific** | 10 (9,11) | 0 (0,0) | 7 (6,8) | 0 (0,0) | -11·1 (-17,-5·2) |
| **Brunei Darussalam** | 0 (0,0) | 0 (0,0·1) | 0 (0,0) | 0 (0,0·1) | 8·7 (-47,94·2) |
| **Japan** | 6 (6,6) | 0 (0,0) | 5 (5,5) | 0 (0,0) | 4 (-1·6,9·4) |
| **Republic of Korea** | 4 (3,5) | 0 (0,0) | 2 (1,3) | 0 (0,0) | -42·1 (-55·6,-26) |
| **Singapore** | 0 (0,0) | 0 (0,0) | 0 (0,0) | 0 (0,0) | -44·7 (-51,-37) |
| **High-income North America** | 176 (170,182) | 0·6 (0·6,0·6) | 161 (155,168) | 0·8 (0·8,0·9) | 40·4 (31·7,49·2) |
| **Canada** | 18 (16,20) | 0·7 (0·7,0·8) | 21 (19,24) | 0·8 (0·8,0·9) | 14·6 (1·5,27·2) |
| **Greenland** | 0 (0,0) | 0·1 (0,0·1) | 0 (0,0) | 0·2 (0·1,0·3) | 202·2 (86·9,413·6) |
| **United States of America** | 158 (153,163) | 0·6 (0·5,0·6) | 140 (134,146) | 0·8 (0·8,0·9) | 43·8 (34·5,53·6) |
| **North Africa and Middle East** | 53 (30,78) | 0·1 (0·1,0·2) | 199 (161,239) | 0·2 (0·2,0·2) | 80 (24·6,214·4) |
| **Afghanistan** | 1 (0,2) | 0·1 (0,0·2) | 10 (4,19) | 0·2 (0·1,0·4) | 156·2 (54·7,455·1) |
| **Algeria** | 3 (2,5) | 0·1 (0,0·1) | 19 (11,31) | 0·2 (0·1,0·3) | 156·7 (43·2,380·3) |
| **Bahrain** | 0 (0,0) | 0 (0,0) | 0 (0,0) | 0·1 (0·1,0·1) | 996·3 (668·3,1426·4) |
| **Egypt** | 1 (0,1) | 0 (0,0) | 2 (1,3) | 0 (0,0) | 58·2 (11·7,140·6) |
| **Iran (Islamic Republic of)** | 22 (11,33) | 0·3 (0·1,0·4) | 74 (61,92) | 0·4 (0·3,0·5) | 53·4 (0·7,190·7) |
| **Iraq** | 1 (1,3) | 0·1 (0,0·1) | 6 (3,11) | 0·1 (0·1,0·1) | 56·9 (-24·1,286·9) |
| **Jordan** | 1 (1,2) | 0·2 (0·2,0·3) | 4 (3,6) | 0·2 (0·2,0·3) | -5·3 (-43·4,60·7) |
| **Kuwait** | 0 (0,0) | 0 (0,0) | 1 (0,1) | 0·1 (0,0·1) | 24716·6 (19820·5,31944·2) |
| **Lebanon** | 0 (0,1) | 0·1 (0·1,0·2) | 1 (1,2) | 0·1 (0·1,0·2) | 26 (-24·3,115·7) |
| **Libya** | 1 (0,1) | 0·1 (0·1,0·1) | 6 (3,10) | 0·4 (0·3,0·6) | 352·1 (160·9,740·5) |
| **Morocco** | 3 (1,5) | 0·1 (0,0·1) | 15 (6,28) | 0·2 (0·1,0·3) | 206·6 (72·8,650·3) |
| **Oman** | 0 (0,0) | 0·1 (0,0·1) | 2 (1,3) | 0·2 (0·1,0·3) | 162·2 (3·2,675·2) |
| **Palestine** | 0 (0,1) | 0·2 (0·1,0·2) | 2 (1,3) | 0·3 (0·2,0·4) | 105·2 (12·2,315·8) |
| **Qatar** | 0 (0,0) | 0 (0,0) | 0 (0,0) | 0 (0,0·1) | 120·1 (29·6,351·7) |
| **Saudi Arabia** | 1 (0,1) | 0 (0,0) | 8 (4,15) | 0·1 (0·1,0·1) | 188·4 (42·8,619·8) |
| **Sudan** | 1 (1,2) | 0 (0,0·1) | 11 (5,19) | 0·1 (0·1,0·2) | 177·2 (38·1,603·6) |
| **Syrian Arab Republic** | 1 (0,2) | 0·1 (0,0·1) | 2 (1,3) | 0·1 (0·1,0·1) | 54·4 (-18·5,230·6) |
| **Tunisia** | 1 (0,2) | 0·1 (0,0·1) | 6 (3,10) | 0·2 (0·1,0·4) | 169·6 (44·1,500·6) |
| **Turkey** | 14 (7,27) | 0·2 (0·1,0·2) | 25 (16,37) | 0·2 (0·2,0·3) | 32·9 (-22,152·2) |
| **United Arab Emirates** | 0 (0,0) | 0·1 (0,0·1) | 1 (0,2) | 0·1 (0,0·1) | 8·2 (-36·3,145·1) |
| **Yemen** | 0 (0,1) | 0 (0,0·1) | 5 (2,9) | 0·1 (0·1,0·2) | 176·5 (53,665·1) |
| **Oceania** | 0 (0,0) | 0 (0,0) | 0 (0,0) | 0 (0,0) | 25·6 (-11·9,89·1) |
| **American Samoa** | 0 (0,0) | 0 (0,0) | 0 (0,0) | 0 (0,0) | 39·5 (-14·2,143) |
| **Cook Islands** | 0 (0,0) | 0 (0,0) | 0 (0,0) | 0 (0,0) | 32·5 (-22·5,155) |
| **Fiji** | 0 (0,0) | 0 (0,0) | 0 (0,0) | 0 (0,0) | 31·6 (-24·4,167·8) |
| **Guam** | 0 (0,0) | 0 (0,0) | 0 (0,0) | 0 (0,0) | -15·4 (-51·7,55·7) |
| **Kiribati** | 0 (0,0) | 0 (0,0) | 0 (0,0) | 0 (0,0) | 20·1 (-29·7,109·2) |
| **Marshall Islands** | 0 (0,0) | 0 (0,0) | 0 (0,0) | 0 (0,0) | 33·9 (-23·3,178·9) |
| **Micronesia (Federated States of)** | 0 (0,0) | 0 (0,0) | 0 (0,0) | 0 (0,0) | 33·5 (-18·3,135·4) |
| **Nauru** | 0 (0,0) | 0 (0,0) | 0 (0,0) | 0 (0,0) | 35 (-23·8,144·7) |
| **Niue** | 0 (0,0) | 0 (0,0) | 0 (0,0) | 0 (0,0) | 103·3 (15·7,293·8) |
| **Northern Mariana Islands** | 0 (0,0) | 0 (0,0) | 0 (0,0) | 0 (0,0) | 21·3 (-23·5,101·1) |
| **Palau** | 0 (0,0) | 0 (0,0) | 0 (0,0) | 0 (0,0) | 55·4 (-17·8,187) |
| **Papua New Guinea** | 0 (0,0) | 0 (0,0) | 0 (0,0) | 0 (0,0) | 39·7 (-19·2,174·8) |
| **Samoa** | 0 (0,0) | 0 (0,0) | 0 (0,0) | 0 (0,0) | 31·8 (-24·8,133·9) |
| **Solomon Islands** | 0 (0,0) | 0 (0,0) | 0 (0,0) | 0 (0,0) | 50·5 (-6,230·6) |
| **Tokelau** | 0 (0,0) | 0 (0,0) | 0 (0,0) | 0 (0,0) | 115·9 (19,318·6) |
| **Tonga** | 0 (0,0) | 0 (0,0) | 0 (0,0) | 0 (0,0) | 50·9 (-12·4,200·7) |
| **Tuvalu** | 0 (0,0) | 0 (0,0) | 0 (0,0) | 0 (0,0) | 38·9 (-15·6,171·8) |
| **Vanuatu** | 0 (0,0) | 0 (0,0) | 0 (0,0) | 0 (0,0) | 47·7 (-14·5,202·9) |
| **South Asia** | 15 (7,25) | 0 (0,0) | 56 (34,75) | 0 (0,0) | 85·6 (24·9,286·3) |
| **Bangladesh** | 1 (0,3) | 0 (0,0) | 4 (1,8) | 0 (0,0) | 79·7 (5·6,315·3) |
| **Bhutan** | 0 (0,0) | 0 (0,0) | 0 (0,0) | 0 (0,0) | 99·6 (20·1,400·7) |
| **India** | 12 (6,20) | 0 (0,0) | 42 (27,56) | 0 (0,0) | 87 (22·2,291·6) |
| **Nepal** | 0 (0,0) | 0 (0,0) | 1 (0,2) | 0 (0,0) | 94·1 (24·5,342·1) |
| **Pakistan** | 2 (0,3) | 0 (0,0) | 9 (4,14) | 0 (0,0) | 82 (11·3,364·8) |
| **Southeast Asia** | 10 (7,13) | 0 (0,0) | 25 (21,30) | 0 (0,0) | 76·4 (30·9,173·4) |
| **Cambodia** | 0 (0,0) | 0 (0,0) | 0 (0,1) | 0 (0,0) | 114·8 (28·7,269·8) |
| **Indonesia** | 2 (1,2) | 0 (0,0) | 6 (4,9) | 0 (0,0) | 119·7 (33·1,286) |
| **Lao People's Democratic Republic** | 0 (0,0) | 0 (0,0) | 0 (0,0) | 0 (0,0) | 113·2 (20·5,347·2) |
| **Malaysia** | 0 (0,1) | 0 (0,0) | 2 (1,2) | 0 (0,0) | 65·2 (-8·8,239·9) |
| **Maldives** | 0 (0,0) | 0 (0,0) | 0 (0,0) | 0 (0,0) | 66 (-8·4,187) |
| **Mauritius** | 0 (0,0) | 0 (0,0) | 0 (0,0) | 0·1 (0,0·1) | 27425·1 (22127·7,33661·5) |
| **Myanmar** | 1 (0,1) | 0 (0,0) | 2 (1,3) | 0 (0,0) | 94·6 (6·5,264·9) |
| **Philippines** | 5 (4,6) | 0 (0,0) | 11 (9,13) | 0 (0,0) | 31·1 (-3·6,107·9) |
| **Seychelles** | 0 (0,0) | 0 (0,0) | 0 (0,0) | 0 (0,0) | 217·8 (64,490·1) |
| **Sri Lanka** | 0 (0,1) | 0 (0,0) | 0 (0,1) | 0 (0,0) | 8·2 (-36·3,70·9) |
| **Thailand** | 1 (0,1) | 0 (0,0) | 1 (1,2) | 0 (0,0) | 95 (24·3,242) |
| **Timor-Leste** | 0 (0,0) | 0 (0,0) | 0 (0,0) | 0 (0,0) | 136·7 (42·1,396·6) |
| **Viet Nam** | 1 (0,2) | 0 (0,0) | 3 (1,5) | 0 (0,0) | 122·2 (33·1,423·2) |
| **Southern Latin America** | 17 (16,19) | 0·3 (0·3,0·3) | 14 (12,16) | 0·2 (0·1,0·2) | -40·4 (-47·2,-33·7) |
| **Uruguay** | 2 (1,2) | 0·4 (0·4,0·5) | 1 (1,2) | 0·3 (0·3,0·4) | -25·6 (-35·6,-16) |
| **Argentina** | 13 (11,14) | 0·3 (0·3,0·3) | 10 (9,12) | 0·2 (0·2,0·2) | -36·7 (-44·3,-29) |
| **Chile** | 3 (3,3) | 0·2 (0·1,0·2) | 2 (2,2) | 0·1 (0·1,0·1) | -48·5 (-55·7,-41·3) |
| **Southern Sub-Saharan Africa** | 4 (3,6) | 0·1 (0·1,0·1) | 5 (4,7) | 0·1 (0·1,0·1) | 29·7 (4,75·7) |
| **Botswana** | 0 (0,0) | 0 (0,0) | 0 (0,0) | 0 (0,0) | 27·6 (-32·7,200·8) |
| **Eswatini** | 0 (0,0) | 0 (0,0) | 0 (0,0) | 0 (0,0) | 69·4 (-15·9,345·8) |
| **Lesotho** | 0 (0,0) | 0 (0,0) | 0 (0,0) | 0 (0,0) | 97·8 (-5,561·6) |
| **Namibia** | 0 (0,0) | 0 (0,0) | 0 (0,0) | 0 (0,0) | 61 (-9·9,228·2) |
| **South Africa** | 4 (3,6) | 0·1 (0·1,0·1) | 5 (4,7) | 0·1 (0·1,0·2) | 23·6 (-1·1,66·8) |
| **Zimbabwe** | 0 (0,0) | 0 (0,0) | 0 (0,0) | 0 (0,0) | 53·5 (-6·5,200·5) |
| **Tropical Latin America** | 18 (17,18) | 0·1 (0·1,0·1) | 33 (31,35) | 0·1 (0·1,0·1) | 20·9 (11·1,31·2) |
| **Brazil** | 17 (17,18) | 0·1 (0·1,0·1) | 32 (30,35) | 0·1 (0·1,0·1) | 19·5 (9·9,29·7) |
| **Paraguay** | 0 (0,0) | 0·1 (0,0·1) | 1 (0,1) | 0·1 (0·1,0·2) | 128·7 (59·4,236·3) |
| **Western Europe** | 254 (245,264) | 0·7 (0·6,0·7) | 201 (190,211) | 0·7 (0·7,0·8) | 10·5 (3·7,17·1) |
| **Andorra** | 0 (0,0) | 0·6 (0·4,1·1) | 0 (0,0) | 0·6 (0·4,0·9) | -5·2 (-52·3,84·9) |
| **Austria** | 5 (4,5) | 0·6 (0·6,0·7) | 4 (4,5) | 0·7 (0·7,0·8) | 15·7 (1·3,29·8) |
| **Belgium** | 7 (6,8) | 0·7 (0·6,0·7) | 7 (6,8) | 0·8 (0·7,0·8) | 12·8 (0,25·1) |
| **Cyprus** | 0 (0,0) | 0·4 (0·2,0·7) | 0 (0,1) | 0·4 (0·3,0·6) | -4·4 (-46·6,100·3) |
| **Denmark** | 5 (5,6) | 1·3 (1·2,1·5) | 4 (3,4) | 1·2 (1·1,1·4) | -8·5 (-23,7·6) |
| **Finland** | 4 (4,5) | 0·7 (0·6,0·8) | 3 (3,4) | 0·7 (0·7,0·8) | 6·4 (-8·2,21·4) |
| **France** | 33 (30,37) | 0·5 (0·5,0·6) | 24 (20,27) | 0·6 (0·5,0·6) | 6·8 (-8·6,21·5) |
| **Germany** | 66 (61,73) | 0·8 (0·7,0·8) | 33 (29,37) | 0·8 (0·8,0·9) | 7·7 (-1·6,20) |
| **Greece** | 4 (3,4) | 0·3 (0·3,0·4) | 4 (4,5) | 0·6 (0·5,0·7) | 75·6 (55·2,97·2) |
| **Iceland** | 0 (0,0) | 0·7 (0·7,0·8) | 0 (0,0) | 0·8 (0·7,0·9) | 12·9 (-2·9,31·6) |
| **Ireland** | 3 (2,3) | 1 (0·9,1) | 3 (3,4) | 0·8 (0·7,0·9) | -12·8 (-27·5,0·8) |
| **Israel** | 1 (1,1) | 0·2 (0·2,0·2) | 2 (1,2) | 0·2 (0·2,0·3) | 8 (-4·4,21·6) |
| **Italy** | 27 (25,28) | 0·4 (0·4,0·4) | 18 (17,20) | 0·5 (0·4,0·5) | 28·3 (17·8,39·1) |
| **Luxembourg** | 0 (0,0) | 0·8 (0·7,0·8) | 0 (0,0) | 0·7 (0·6,0·7) | -16·3 (-26·8,-4·7) |
| **Malta** | 0 (0,0) | 0·3 (0·2,0·3) | 0 (0,0) | 0·3 (0·3,0·4) | 16·7 (0,36·6) |
| **Monaco** | 0 (0,0) | 0·2 (0·1,0·3) | 0 (0,0) | 0·3 (0·2,0·5) | 58·8 (-13,186·4) |
| **Netherlands** | 11 (10,13) | 0·8 (0·8,0·9) | 10 (9,11) | 0·9 (0·8,0·9) | 3·9 (-6·4,15·6) |
| **Norway** | 3 (3,3) | 1·1 (1,1·2) | 3 (2,3) | 1 (0·9,1·1) | -7·2 (-14·6,1·2) |
| **Portugal** | 4 (3,4) | 0·3 (0·3,0·3) | 2 (2,3) | 0·3 (0·3,0·3) | 1·8 (-9·1,13·2) |
| **San Marino** | 0 (0,0) | 0 (0,0) | 0 (0,0) | 0 (0,0) | -22·5 (-63·1,38·6) |
| **Spain** | 13 (11,14) | 0·3 (0·3,0·3) | 8 (7,10) | 0·3 (0·3,0·3) | 8·5 (-3·4,22·2) |
| **Sweden** | 5 (4,5) | 0·7 (0·6,0·7) | 5 (4,6) | 0·8 (0·7,0·9) | 16·3 (0·4,32·5) |
| **Switzerland** | 7 (6,9) | 1·1 (1,1·2) | 5 (4,6) | 0·9 (0·8,1) | -20 (-32·9,-6·3) |
| **United Kingdom** | 56 (54,57) | 1·1 (1·1,1·1) | 65 (63,68) | 1·3 (1·3,1·4) | 20·4 (14·7,26) |
| **Western Sub-Saharan Africa** | 75 (40,113) | 0 (0,0·1) | 343 (209,499) | 0·1 (0,0·1) | 71·5 (5·6,203·7) |
| **Benin** | 2 (0,4) | 0 (0,0·1) | 9 (3,22) | 0·1 (0,0·1) | 63·7 (-54,557·3) |
| **Burkina Faso** | 3 (1,8) | 0 (0,0·1) | 14 (3,39) | 0·1 (0,0·2) | 64 (-59·5,610·4) |
| **Cabo Verde** | 0 (0,1) | 0 (0,0·1) | 0 (0,1) | 0·1 (0,0·1) | 11 (-69·7,346·6) |
| **Cameroon** | 5 (2,11) | 0 (0,0·1) | 26 (8,66) | 0·1 (0,0·2) | 55·9 (-65·6,536·8) |
| **Chad** | 1 (0,4) | 0 (0,0·1) | 8 (2,23) | 0 (0,0·1) | 76·9 (-59·8,639·3) |
| **CÃ´te d'Ivoire** | 5 (1,13) | 0 (0,0·1) | 19 (6,53) | 0·1 (0,0·2) | 78·4 (-54·1,595·1) |
| **Gambia** | 0 (0,1) | 0 (0,0·1) | 2 (1,6) | 0·1 (0,0·2) | 133·7 (-51,959·9) |
| **Ghana** | 10 (3,24) | 0·1 (0,0·1) | 35 (11,88) | 0·1 (0,0·2) | 39·4 (-62·9,450·3) |
| **Guinea** | 2 (0,5) | 0 (0,0·1) | 9 (3,24) | 0·1 (0,0·2) | 107·4 (-44·7,765·6) |
| **Guinea-Bissau** | 1 (0,1) | 0 (0,0·1) | 2 (1,5) | 0·1 (0,0·2) | 62·6 (-55,475·3) |
| **Liberia** | 1 (0,2) | 0 (0,0·1) | 4 (1,11) | 0·1 (0,0·2) | 99·7 (-47·5,608·7) |
| **Mali** | 4 (1,10) | 0 (0,0·1) | 20 (4,48) | 0·1 (0,0·2) | 60·6 (-56·6,452·3) |
| **Mauritania** | 1 (0,3) | 0 (0,0·1) | 4 (1,10) | 0·1 (0,0·2) | 69 (-57·3,553·9) |
| **Niger** | 2 (0,7) | 0 (0,0·1) | 10 (1,29) | 0 (0,0·1) | 31 (-69·1,435·6) |
| **Nigeria** | 31 (14,53) | 0 (0,0·1) | 154 (82,274) | 0·1 (0,0·1) | 90·2 (-2·9,306·7) |
| **Sao Tome and Principe** | 0 (0,0) | 0 (0,0) | 0 (0,0) | 0 (0,0) | 115·3 (-65,1061·4) |
| **Senegal** | 4 (1,10) | 0 (0,0·1) | 14 (4,37) | 0·1 (0,0·2) | 60·8 (-65·4,546·5) |
| **Sierra Leone** | 1 (0,4) | 0 (0,0·1) | 6 (2,16) | 0·1 (0,0·1) | 118·3 (-53·9,1098·5) |
| **Togo** | 2 (1,4) | 0 (0,0·1) | 6 (2,15) | 0·1 (0,0·2) | 48·2 (-62·2,417·5) |

**Table S4**

| **Table S4: DALYs due to MS in 1990 and 2021 and the percentage change in the age-standardised rates (ASRs) per 100,000, by location (Generated from data available from [http://ghdx·healthdata·org/gbd-results-tool](http://ghdx.healthdata.org/gbd-results-tool))** | | | | | |
| --- | --- | --- | --- | --- | --- |
|  | **1990** | | **2021** | | Percentage change in the ASRs per 100000 |
|  | No (95%UI) | ASRs per 100000 (95%UI) | No (95%UI) | ASRs per 100000 (95%UI) |  |
| **Global** | 160530 (130796,196736) | 12·8 (11·1,14·7) | 215869 (173537,268521) | 11·4 (9·8,13·2) | -11 (-14,-8) |
| **Andean Latin America** | 310 (241,401) | 3·7 (3,4·6) | 875 (653,1130) | 5·9 (4·7,7·3) | 62·1 (38·3,89·5) |
| **Bolivia (Plurinational State of)** | 63 (40,94) | 4·7 (3·2,6·8) | 195 (117,296) | 7·5 (5·3,10·5) | 58·2 (13·6,127·7) |
| **Ecuador** | 88 (73,108) | 4 (3·5,4·6) | 249 (199,306) | 6·2 (5,7·5) | 55·8 (31·9,88·4) |
| **Peru** | 159 (119,212) | 3·2 (2·5,4·1) | 431 (305,581) | 5·4 (4·1,7) | 66·1 (34·3,109·2) |
| **Australasia** | 1133 (882,1429) | 23·6 (20·1,27·4) | 1954 (1447,2579) | 28·8 (23·7,34·4) | 22·2 (9·5,36·6) |
| **Australia** | 955 (733,1226) | 23 (19·5,27) | 1722 (1262,2304) | 29·7 (24·4,36) | 29 (14,46·4) |
| **New Zealand** | 178 (135,232) | 26·2 (22·7,30·1) | 233 (177,294) | 24·1 (20·4,27·6) | -8·1 (-20·4,4·8) |
| **Caribbean** | 985 (865,1134) | 9·1 (8·2,10·4) | 1353 (1111,1683) | 10·7 (9·3,12·6) | 17·9 (5·5,31) |
| **Antigua and Barbuda** | 2 (2,3) | 11·9 (10·4,13·6) | 4 (3,5) | 15·7 (13·5,18) | 32 (14·7,52·3) |
| **Bahamas** | 12 (11,14) | 13·8 (12·4,15·2) | 29 (23,36) | 19·9 (15·8,24·4) | 44·4 (13,79·3) |
| **Barbados** | 14 (12,15) | 15·6 (14·1,17·4) | 17 (13,22) | 21·7 (17·3,26·5) | 39·2 (11·2,70·2) |
| **Belize** | 2 (2,3) | 4 (3·3,4·8) | 9 (8,11) | 6·4 (5·5,7·6) | 61·8 (43·8,84) |
| **Bermuda** | 3 (2,3) | 13 (11·6,14·6) | 2 (1,2) | 12 (10·1,14·5) | -7·4 (-20·6,7·9) |
| **Cuba** | 466 (415,523) | 12·3 (11·2,13·7) | 388 (321,466) | 15·5 (13·3,18) | 25·5 (9·3,43·5) |
| **Dominica** | 1 (1,1) | 4·6 (3·7,5·7) | 1 (1,2) | 6·6 (5,8·9) | 41·9 (11·1,81·5) |
| **Dominican Republic** | 97 (72,124) | 4·1 (3·2,5·2) | 204 (138,293) | 5·3 (4,6·9) | 30·7 (3,78·3) |
| **Grenada** | 4 (3,4) | 13·7 (12·2,15·3) | 5 (4,6) | 16·1 (13·5,18·7) | 17·8 (-0·7,39·2) |
| **Guyana** | 9 (7,11) | 3·9 (3·3,4·6) | 15 (11,19) | 6 (4·6,7·6) | 54·9 (19·1,97·4) |
| **Haiti** | 118 (75,198) | 6·2 (3·8,9·5) | 369 (223,619) | 8·2 (5,13·5) | 33·5 (-11·5,97) |
| **Jamaica** | 30 (24,37) | 5 (4·3,6) | 71 (55,92) | 8·1 (6·4,10·3) | 61·6 (31·1,98·7) |
| **Puerto Rico** | 155 (138,171) | 13·1 (12,14·5) | 122 (102,146) | 14·6 (12·1,17·4) | 11·3 (-6·1,30·9) |
| **Saint Kitts and Nevis** | 3 (2,3) | 20·8 (19·1,22·9) | 3 (2,4) | 19·4 (16·1,23·2) | -7 (-23·6,10·8) |
| **Saint Lucia** | 3 (3,4) | 8·3 (7·5,9·4) | 6 (5,7) | 10 (8·2,12) | 19·9 (1·7,41) |
| **Saint Vincent and the Grenadines** | 2 (1,2) | 5·4 (4·6,6·3) | 3 (2,3) | 7·2 (6,8·6) | 33·2 (14·8,55·9) |
| **Suriname** | 6 (4,8) | 4·8 (3·7,5·7) | 12 (8,17) | 6·5 (4·7,8·7) | 36·4 (1·6,81·8) |
| **Trinidad and Tobago** | 23 (20,27) | 6·4 (5·7,7·2) | 45 (35,58) | 9·3 (7·4,11·8) | 44·7 (14·3,80·1) |
| **United States Virgin Islands** | 3 (2,4) | 8·9 (6·4,11·5) | 3 (1,4) | 9·9 (6·8,13·7) | 10·2 (-24·9,55·6) |
| **Central Asia** | 1691 (1381,2068) | 13·2 (10·9,15·7) | 1866 (1387,2455) | 10·8 (8·4,13·7) | -18·3 (-27·9,-9·8) |
| **Armenia** | 71 (54,92) | 9·9 (7·7,12·3) | 56 (40,76) | 11·6 (9,14·4) | 17·6 (-0·5,37·6) |
| **Azerbaijan** | 115 (81,159) | 6·8 (5·2,8·9) | 167 (111,235) | 6·7 (5·1,8·9) | -0·8 (-19·1,21·2) |
| **Georgia** | 84 (61,113) | 7·2 (5·5,9·1) | 51 (34,70) | 8·7 (6·5,11) | 20·9 (5·7,39·1) |
| **Kazakhstan** | 819 (696,975) | 26·2 (21·9,30·9) | 573 (408,762) | 21·1 (15·9,26·8) | -19·8 (-35·2,-5·5) |
| **Kyrgyzstan** | 77 (59,99) | 7·7 (6·2,9·5) | 110 (81,145) | 7·2 (5·6,9·2) | -6·5 (-18·5,7) |
| **Mongolia** | 42 (25,72) | 10·6 (6·7,16·9) | 85 (51,138) | 11·9 (7·8,18·5) | 12·1 (-39·5,99·6) |
| **Tajikistan** | 61 (43,86) | 5·8 (4·3,7·8) | 137 (91,197) | 5·6 (4·1,7·6) | -3·9 (-22·6,18·2) |
| **Turkmenistan** | 112 (91,137) | 16·7 (13·8,19·5) | 142 (96,193) | 15·1 (10·7,19·1) | -9·6 (-32·9,13·9) |
| **Uzbekistan** | 310 (222,418) | 8·6 (6·6,11·1) | 545 (382,743) | 7 (5·4,8·9) | -18·4 (-29·9,-6·5) |
| **Central Europe** | 15909 (14141,17986) | 43·3 (40,47·1) | 8182 (6642,9853) | 29·9 (26·3,33·7) | -31·1 (-36·7,-25·5) |
| **Albania** | 586 (411,819) | 72·8 (55·5,93) | 341 (208,551) | 56·7 (40·3,82·3) | -22·2 (-49·5,17) |
| **Bosnia and Herzegovina** | 360 (272,464) | 26·4 (20·7,33·6) | 138 (101,189) | 18·7 (13·8,25) | -29 (-49·8,5) |
| **Bulgaria** | 839 (724,960) | 38·6 (34·5,43·2) | 502 (408,624) | 35·6 (30·3,41·7) | -7·9 (-22,8·3) |
| **Croatia** | 409 (361,468) | 31·1 (28·1,34·6) | 203 (157,254) | 23·3 (19·7,27·1) | -25·1 (-36·4,-13) |
| **Czechia** | 1191 (1025,1405) | 48·6 (44·3,53·6) | 642 (507,805) | 28 (23·4,33·1) | -42·4 (-50·9,-33·2) |
| **Hungary** | 1277 (1086,1492) | 42·8 (38·4,47·8) | 540 (416,679) | 27 (23·1,31·2) | -36·9 (-44·5,-26·7) |
| **Montenegro** | 62 (44,87) | 34·9 (25·5,47·3) | 54 (40,70) | 35·3 (27·4,44) | 1·3 (-31·2,45·7) |
| **North Macedonia** | 154 (115,201) | 28·2 (21·9,35·7) | 152 (110,204) | 27·2 (21·2,34·8) | -3·3 (-26·5,29·6) |
| **Poland** | 7113 (6283,8003) | 60 (55·3,65·1) | 3594 (2860,4368) | 36·3 (31·3,41) | -39·5 (-45·1,-33·5) |
| **Romania** | 1942 (1719,2178) | 26·7 (24·1,29·4) | 622 (502,753) | 15·4 (13,17·7) | -42·3 (-51·1,-33·4) |
| **Serbia** | 1052 (736,1494) | 37·9 (28,52·3) | 829 (567,1112) | 38·4 (28·8,49·5) | 1·3 (-30·7,41·6) |
| **Slovakia** | 399 (298,539) | 26·5 (20·9,33·9) | 327 (233,448) | 23·5 (17·9,31·4) | -11 (-36·8,25·2) |
| **Slovenia** | 272 (235,313) | 50·6 (45·8,55·6) | 119 (92,152) | 28·5 (23·5,33·9) | -43·6 (-51·7,-34·2) |
| **Central Latin America** | 2554 (2227,2964) | 5·5 (4·9,6·2) | 7656 (6611,8873) | 10·5 (9·2,11·9) | 91·2 (71·8,111·6) |
| **Colombia** | 478 (418,541) | 4·8 (4·3,5·3) | 1027 (852,1216) | 6·8 (5·7,8·1) | 43·7 (22,66) |
| **Costa Rica** | 44 (37,53) | 5·8 (5·1,6·7) | 147 (122,174) | 10·8 (9·4,12·4) | 87·7 (65·3,111·9) |
| **El Salvador** | 42 (32,54) | 3·1 (2·5,3·8) | 99 (75,130) | 5·7 (4·4,7·1) | 83·9 (48·2,137·1) |
| **Guatemala** | 87 (75,103) | 4·1 (3·6,4·7) | 286 (234,347) | 6·3 (5·3,7·6) | 55·6 (37·2,75·1) |
| **Honduras** | 24 (17,34) | 2·1 (1·6,2·8) | 79 (54,116) | 2·9 (2·1,3·8) | 34·3 (8·5,67·2) |
| **Mexico** | 1527 (1326,1777) | 6·3 (5·6,7·2) | 4909 (4178,5697) | 13 (11·2,14·9) | 107·7 (84·8,132·3) |
| **Nicaragua** | 34 (26,44) | 3·5 (2·9,4·4) | 111 (82,149) | 6·1 (4·7,7·6) | 70·7 (38·2,112) |
| **Panama** | 26 (21,31) | 3·7 (3·2,4·4) | 82 (65,100) | 6·7 (5·4,8·1) | 80 (48·8,114·4) |
| **Venezuela (Bolivarian Republic of)** | 291 (251,337) | 5·8 (5·2,6·5) | 916 (710,1166) | 11·9 (9·4,14·9) | 105·4 (62·3,160·4) |
| **Central Sub-Saharan Africa** | 236 (161,347) | 1·6 (1·1,2·2) | 727 (495,1022) | 1·9 (1·4,2·6) | 23·3 (5·2,43·3) |
| **Angola** | 53 (36,79) | 1·8 (1·2,2·6) | 203 (133,300) | 2·4 (1·6,3·3) | 32·5 (10,61·4) |
| **Central African Republic** | 12 (8,19) | 1·6 (1·1,2·3) | 28 (19,40) | 1·8 (1·2,2·5) | 10·6 (-3·9,31·4) |
| **Congo** | 11 (7,15) | 1·7 (1·2,2·2) | 34 (23,47) | 2·1 (1·6,2·8) | 25·6 (2,58·3) |
| **Democratic Republic of the Congo** | 154 (103,230) | 1·5 (1,2·1) | 440 (293,625) | 1·8 (1·2,2·4) | 18·7 (1,43·6) |
| **Equatorial Guinea** | 2 (1,2) | 1·5 (1,2·1) | 10 (7,14) | 2·2 (1·6,3) | 51·2 (8·6,121·5) |
| **Gabon** | 4 (3,6) | 1·7 (1·3,2·2) | 11 (8,16) | 2·2 (1·6,2·9) | 30·3 (0,80·6) |
| **East Asia** | 3703 (2513,5419) | 0·6 (0·5,0·9) | 4573 (3163,6426) | 0·9 (0·6,1·1) | 35·7 (14·2,55·7) |
| **China** | 3519 (2383,5146) | 0·6 (0·5,0·9) | 4241 (2912,6008) | 0·8 (0·6,1·1) | 33·6 (11·5,54) |
| **Democratic People's Republic of Korea** | 100 (66,149) | 1·1 (0·8,1·5) | 144 (95,204) | 1·3 (0·9,1·8) | 19 (3·6,39·7) |
| **Taiwan (Province of China)** | 85 (61,116) | 0·9 (0·7,1·2) | 188 (130,246) | 2·2 (1·6,2·8) | 137·1 (112·4,171·7) |
| **Eastern Europe** | 25878 (23698,28604) | 28·7 (26·6,31·1) | 13759 (11693,15783) | 20·4 (17·7,22·8) | -28·8 (-36·1,-21·8) |
| **Belarus** | 770 (682,871) | 21 (19,23·3) | 467 (376,575) | 16·5 (13·3,20) | -21·3 (-35·9,-5·2) |
| **Estonia** | 237 (210,267) | 45·6 (41·5,49·6) | 64 (52,76) | 18·5 (15·5,21·5) | -59·4 (-66·6,-52·4) |
| **Latvia** | 442 (390,502) | 51·4 (46·9,56) | 126 (103,150) | 27·1 (23,31·3) | -47·2 (-56,-38·1) |
| **Lithuania** | 574 (511,648) | 48·3 (42·9,54·1) | 177 (146,206) | 27·2 (22·7,31·6) | -43·6 (-53·2,-33) |
| **Republic of Moldova** | 161 (139,183) | 10 (9,11·1) | 83 (67,100) | 6·9 (5·7,8·2) | -31·7 (-41·9,-20·9) |
| **Russian Federation** | 16558 (15002,18460) | 27 (24·8,29·7) | 9048 (7662,10474) | 19·8 (17·5,22·2) | -26·6 (-33·2,-19·8) |
| **Ukraine** | 7136 (6302,8021) | 33·5 (30·6,37) | 3795 (2837,4896) | 23·8 (17·8,30·1) | -29·2 (-45·9,-11·2) |
| **Eastern Sub-Saharan Africa** | 898 (607,1326) | 1·7 (1·2,2·4) | 2552 (1697,3593) | 2 (1·4,2·7) | 19·8 (8·2,35·7) |
| **Burundi** | 24 (16,36) | 1·5 (1,2·1) | 65 (43,92) | 1·6 (1,2·2) | 5·3 (-8·2,25·6) |
| **Comoros** | 2 (2,4) | 1·9 (1·3,2·7) | 6 (4,8) | 2·4 (1·7,3·3) | 24·4 (4·3,53·3) |
| **Djibouti** | 2 (1,3) | 1·9 (1·2,2·7) | 10 (6,14) | 2·3 (1·5,3·3) | 23·5 (2·5,53·7) |
| **Eritrea** | 19 (12,28) | 2 (1·4,2·9) | 52 (34,77) | 2·6 (1·7,3·6) | 27·6 (8·4,57·6) |
| **Ethiopia** | 244 (165,365) | 1·7 (1·2,2·5) | 649 (421,946) | 2 (1·3,2·7) | 12·8 (-4·1,35·8) |
| **Kenya** | 91 (61,130) | 1·5 (1,2) | 299 (213,403) | 2 (1·5,2·6) | 34 (17·8,57·1) |
| **Madagascar** | 75 (49,107) | 2·1 (1·5,3) | 218 (144,313) | 2·4 (1·6,3·4) | 12·7 (-2·6,36·7) |
| **Malawi** | 51 (34,77) | 1·8 (1·2,2·5) | 130 (81,196) | 2·3 (1·4,3·3) | 25·1 (9·1,50·3) |
| **Mozambique** | 75 (48,111) | 2 (1·3,2·8) | 215 (127,333) | 2·5 (1·6,3·6) | 26·4 (9·1,56·5) |
| **Rwanda** | 34 (23,48) | 1·6 (1·2,2·2) | 77 (51,112) | 1·8 (1·3,2·5) | 15·3 (-5·3,42·4) |
| **Somalia** | 33 (21,50) | 1·4 (1,2·1) | 91 (59,132) | 1·5 (1,2·1) | 5·7 (-6·4,22·1) |
| **South Sudan** | 25 (16,37) | 1·5 (1,2·1) | 45 (29,66) | 1·8 (1·1,2·5) | 18·3 (2·6,44·9) |
| **Uganda** | 60 (39,90) | 1·3 (0·9,1·8) | 201 (132,288) | 1·7 (1·1,2·4) | 30·5 (13·8,59·6) |
| **United Republic of Tanzania** | 117 (77,170) | 1·7 (1·1,2·3) | 339 (224,487) | 2 (1·4,2·8) | 21·1 (1,48·7) |
| **Zambia** | 44 (29,65) | 2 (1·3,2·8) | 153 (102,216) | 2·5 (1·8,3·5) | 27·2 (0·6,56·1) |
| **High-income Asia Pacific** | 1917 (1429,2553) | 3·7 (2·9,4·7) | 1523 (1096,2006) | 3·7 (2·8,4·7) | -0·3 (-3·8,2·7) |
| **Brunei Darussalam** | 2 (1,3) | 2·1 (1·4,3·3) | 4 (2,6) | 2·3 (1·4,3·7) | 7·7 (-14·1,44·2) |
| **Japan** | 1236 (898,1654) | 3·6 (2·8,4·6) | 1001 (728,1320) | 3·9 (3,4·9) | 6·7 (3·7,9·6) |
| **Republic of Korea** | 658 (493,873) | 3·9 (3·1,5) | 490 (355,658) | 3·4 (2·6,4·5) | -12·3 (-20·4,-4·7) |
| **Singapore** | 21 (15,29) | 1·8 (1·4,2·3) | 28 (19,40) | 1·6 (1·2,2·1) | -11·9 (-18,-6·6) |
| **High-income North America** | 35119 (26940,44246) | 44 (36·7,51·9) | 38917 (30004,48310) | 49·2 (41·8,56·5) | 11·8 (5·6,18·5) |
| **Canada** | 3879 (2952,4869) | 52·1 (44·3,60·5) | 4717 (3555,5865) | 59·2 (49,69·1) | 13·7 (4·9,23·2) |
| **Greenland** | 6 (4,8) | 16·5 (11·9,21·5) | 5 (3,7) | 21·5 (16·6,27·9) | 30·1 (9·1,58·3) |
| **United States of America** | 31233 (23765,39779) | 43·1 (35·9,51·1) | 34195 (26374,42720) | 48 (40·8,55·2) | 11·4 (4·8,18·9) |
| **North Africa and Middle East** | 12059 (8953,16145) | 12·6 (9·7,16·5) | 35679 (27758,45344) | 17·9 (14·5,21·7) | 41·5 (24,63·3) |
| **Afghanistan** | 241 (159,351) | 11·6 (7·9,16·6) | 1645 (1118,2365) | 19·7 (13·9,27) | 69·4 (35·8,111·3) |
| **Algeria** | 820 (559,1142) | 11·4 (8·4,15·6) | 3056 (2136,4179) | 20·4 (16·1,26·2) | 79·3 (40·9,121·1) |
| **Bahrain** | 16 (10,23) | 7 (4·7,9·7) | 73 (49,103) | 12·7 (9·6,16·5) | 80·2 (50·9,115·6) |
| **Egypt** | 817 (545,1146) | 4·8 (3·3,6·6) | 3450 (2159,4952) | 9·7 (6·6,13·6) | 100·6 (71·5,133·2) |
| **Iran (Islamic Republic of)** | 3470 (2440,4608) | 22·9 (16·7,29·7) | 8549 (6957,10532) | 28 (23·3,33·1) | 22·1 (-0·1,54·7) |
| **Iraq** | 496 (335,722) | 9·8 (7·2,13·4) | 1787 (1175,2510) | 13·6 (9·7,17·9) | 38·8 (15,69) |
| **Jordan** | 200 (147,273) | 20·9 (16·5,26·8) | 836 (595,1128) | 21·7 (17,27·4) | 3·8 (-17·5,33·4) |
| **Kuwait** | 62 (40,92) | 8·2 (5·5,11·4) | 360 (240,513) | 15·9 (11·5,21) | 94·9 (66·8,132·2) |
| **Lebanon** | 113 (76,162) | 13·3 (9·8,18·1) | 399 (274,572) | 19·6 (15·1,25·7) | 47·3 (19·1,79·1) |
| **Libya** | 131 (91,187) | 11·3 (8·4,14·8) | 679 (457,957) | 27·9 (21·3,36·8) | 147·4 (86·2,237·1) |
| **Morocco** | 847 (572,1198) | 10·7 (7·5,14·6) | 2420 (1600,3446) | 20·4 (15·2,27·5) | 90·4 (48·8,145·5) |
| **Oman** | 60 (40,86) | 9·4 (6·9,12·5) | 355 (239,496) | 17·4 (12·3,23·1) | 85·2 (37·4,161·8) |
| **Palestine** | 67 (45,95) | 13·8 (10·1,18·7) | 337 (244,444) | 22·9 (18·2,28·6) | 66·3 (26·6,113·4) |
| **Qatar** | 23 (14,35) | 10·2 (6·8,14·4) | 298 (196,420) | 17·4 (12·4,22·7) | 71·2 (44·2,106·7) |
| **Saudi Arabia** | 351 (230,513) | 6·9 (4·8,9·3) | 1977 (1319,2814) | 11·3 (8·5,14·9) | 64 (36·7,99·9) |
| **Sudan** | 399 (274,563) | 7·1 (5·1,9·6) | 1771 (1237,2423) | 12·7 (9·6,16·2) | 78·7 (40·3,142·4) |
| **Syrian Arab Republic** | 340 (231,472) | 10 (7·3,13·3) | 536 (363,767) | 14·7 (10·9,19·7) | 47·4 (20,78·3) |
| **Tunisia** | 317 (220,458) | 12·5 (9·1,17·1) | 894 (601,1242) | 23·1 (17·1,29·8) | 84·3 (49·9,135·9) |
| **Turkey** | 2998 (2188,4079) | 17·6 (13·4,22·9) | 4738 (3552,6143) | 19·4 (15·5,23·7) | 10·3 (-10·6,36·5) |
| **United Arab Emirates** | 66 (43,95) | 7·9 (5·6,10·8) | 400 (253,582) | 8·3 (6·2,10·9) | 6·1 (-12·5,31·7) |
| **Yemen** | 221 (142,328) | 6·7 (4·7,9·4) | 1086 (743,1576) | 11·3 (8·3,15·6) | 69 (39·7,114·6) |
| **Oceania** | 13 (8,21) | 0·5 (0·3,0·7) | 29 (17,45) | 0·5 (0·3,0·7) | 0·7 (-2·6,4·3) |
| **American Samoa** | 0 (0,0) | 0·5 (0·4,0·8) | 0 (0,0) | 0·6 (0·4,0·8) | 4·8 (0·2,10) |
| **Cook Islands** | 0 (0,0) | 0·7 (0·4,1) | 0 (0,0) | 0·7 (0·5,1) | 6·8 (1·4,11·9) |
| **Fiji** | 2 (1,3) | 0·6 (0·4,0·8) | 2 (1,4) | 0·6 (0·4,0·8) | 4·2 (-0·5,9·5) |
| **Guam** | 0 (0,1) | 0·6 (0·4,0·8) | 0 (0,1) | 0·6 (0·4,0·8) | 3·2 (-1·2,8·5) |
| **Kiribati** | 0 (0,0) | 0·4 (0·3,0·6) | 0 (0,0) | 0·5 (0·3,0·7) | 11·2 (5·5,17·8) |
| **Marshall Islands** | 0 (0,0) | 0·5 (0·3,0·7) | 0 (0,0) | 0·5 (0·3,0·7) | 8 (2·2,14·6) |
| **Micronesia (Federated States of)** | 0 (0,0) | 0·5 (0·3,0·7) | 0 (0,0) | 0·5 (0·3,0·7) | 9·8 (4·4,15·7) |
| **Nauru** | 0 (0,0) | 0·4 (0·3,0·6) | 0 (0,0) | 0·4 (0·3,0·6) | 8·6 (4·4,14·8) |
| **Niue** | 0 (0,0) | 0·7 (0·4,0·9) | 0 (0,0) | 0·7 (0·5,1) | 5·7 (0·7,11·3) |
| **Northern Mariana Islands** | 0 (0,0) | 0·6 (0·4,0·9) | 0 (0,0) | 0·7 (0·4,0·9) | 7·2 (2·3,12·3) |
| **Palau** | 0 (0,0) | 0·5 (0·3,0·7) | 0 (0,0) | 0·5 (0·3,0·7) | 6·1 (1,11·1) |
| **Papua New Guinea** | 7 (4,12) | 0·4 (0·3,0·6) | 20 (12,32) | 0·4 (0·3,0·6) | 3·2 (-1·9,8·6) |
| **Samoa** | 0 (0,1) | 0·6 (0·4,0·8) | 0 (0,1) | 0·6 (0·4,0·8) | 6 (1·2,11) |
| **Solomon Islands** | 1 (0,1) | 0·6 (0·4,0·8) | 2 (1,3) | 0·6 (0·4,0·9) | 11 (5·6,18·7) |
| **Tokelau** | 0 (0,0) | 0·5 (0·3,0·7) | 0 (0,0) | 0·5 (0·4,0·8) | 7·5 (3·1,12·3) |
| **Tonga** | 0 (0,0) | 0·6 (0·4,0·9) | 0 (0,0) | 0·7 (0·5,1) | 6·2 (1·4,11) |
| **Tuvalu** | 0 (0,0) | 0·5 (0·3,0·7) | 0 (0,0) | 0·5 (0·3,0·7) | 5·4 (0·7,11·2) |
| **Vanuatu** | 0 (0,1) | 0·5 (0·3,0·8) | 1 (0,1) | 0·6 (0·4,0·8) | 6·3 (0·4,11·9) |
| **South Asia** | 8001 (5408,11623) | 2·3 (1·7,3·2) | 19216 (13387,26558) | 3 (2·2,3·9) | 28·4 (18·5,39·2) |
| **Bangladesh** | 730 (477,1081) | 2·2 (1·6,3·1) | 1562 (1063,2233) | 2·8 (2,3·7) | 22·7 (10,39·6) |
| **Bhutan** | 5 (3,7) | 2·4 (1·7,3·3) | 9 (6,12) | 3 (2·2,4·1) | 28·1 (15·9,46·5) |
| **India** | 6287 (4258,9095) | 2·3 (1·6,3·1) | 14499 (10087,19985) | 2·9 (2·2,3·8) | 28·8 (17·4,40·7) |
| **Nepal** | 141 (93,208) | 2·4 (1·7,3·3) | 324 (217,467) | 3 (2·2,4·2) | 28·9 (17·7,44·2) |
| **Pakistan** | 838 (554,1215) | 2·7 (1·9,3·8) | 2822 (1974,3947) | 3·5 (2·5,4·6) | 26·5 (15,41·1) |
| **Southeast Asia** | 1862 (1353,2596) | 0·9 (0·7,1·2) | 3487 (2702,4544) | 1·2 (0·9,1·4) | 30·8 (16·5,49·4) |
| **Cambodia** | 29 (20,43) | 0·7 (0·5,1) | 74 (48,111) | 1 (0·7,1·3) | 34·1 (15·6,64) |
| **Indonesia** | 562 (380,812) | 0·7 (0·5,0·9) | 1098 (776,1500) | 0·9 (0·7,1·2) | 32·4 (15,60·3) |
| **Lao People's Democratic Republic** | 13 (8,20) | 0·8 (0·5,1·1) | 36 (23,53) | 1 (0·7,1·4) | 30·2 (12,60·4) |
| **Malaysia** | 66 (43,98) | 0·9 (0·6,1·3) | 169 (122,228) | 1·3 (1,1·6) | 36·3 (0·8,86·9) |
| **Maldives** | 0 (0,1) | 0·6 (0·4,0·9) | 2 (1,3) | 0·7 (0·5,1) | 17·3 (0·7,41·4) |
| **Mauritius** | 4 (2,6) | 0·7 (0·5,1) | 15 (13,17) | 2·9 (2·6,3·4) | 303·1 (207·9,468·6) |
| **Myanmar** | 171 (116,254) | 0·9 (0·6,1·2) | 280 (194,398) | 1·1 (0·8,1·6) | 28·6 (6·7,57·8) |
| **Philippines** | 474 (364,610) | 1·7 (1·3,2·1) | 986 (799,1216) | 2 (1·7,2·4) | 18·5 (-2·2,54·8) |
| **Seychelles** | 0 (0,0) | 0·8 (0·6,1·1) | 1 (0,1) | 1·2 (0·9,1·7) | 54·7 (30·8,89·6) |
| **Sri Lanka** | 73 (52,104) | 0·9 (0·7,1·2) | 83 (58,117) | 1 (0·8,1·3) | 9·7 (-6·6,29·8) |
| **Thailand** | 217 (144,315) | 0·8 (0·6,1·1) | 250 (177,342) | 1 (0·8,1·4) | 26·6 (12·4,46·6) |
| **Timor-Leste** | 2 (1,3) | 0·6 (0·4,0·8) | 4 (3,6) | 0·8 (0·5,1·1) | 31·7 (16·3,56·9) |
| **Viet Nam** | 247 (158,378) | 0·9 (0·6,1·3) | 484 (321,699) | 1·2 (0·9,1·7) | 38·6 (17·7,68·5) |
| **Southern Latin America** | 1928 (1579,2387) | 15·2 (13·3,17·7) | 2189 (1630,2779) | 11·4 (9·4,13·7) | -24·8 (-32,-17·8) |
| **Uruguay** | 152 (126,180) | 21·1 (18·7,23·7) | 154 (124,191) | 17·9 (15·3,20·9) | -15·2 (-24·4,-4·4) |
| **Argentina** | 1374 (1125,1687) | 16·4 (14·3,19·2) | 1572 (1167,1977) | 12·4 (10·2,14·9) | -23·9 (-31·9,-15·6) |
| **Chile** | 402 (313,515) | 10·4 (8·7,12·4) | 462 (329,624) | 8·1 (6·4,10·2) | -22·2 (-31·2,-12) |
| **Southern Sub-Saharan Africa** | 576 (439,751) | 4·6 (3·6,5·7) | 886 (672,1148) | 5·3 (4·3,6·4) | 14·9 (-0·4,34·1) |
| **Botswana** | 7 (5,10) | 2 (1·4,2·7) | 19 (13,28) | 2·2 (1·6,3) | 11·3 (-3·6,27·2) |
| **Eswatini** | 5 (3,7) | 2·2 (1·6,2·9) | 9 (6,13) | 2·6 (1·9,3·4) | 18·1 (0·9,41·8) |
| **Lesotho** | 9 (6,13) | 2·2 (1·5,3) | 15 (10,22) | 2·6 (1·9,3·5) | 18·7 (4,38·7) |
| **Namibia** | 8 (5,11) | 2 (1·4,2·7) | 17 (11,24) | 2·3 (1·7,3·1) | 18·6 (4·2,38·3) |
| **South Africa** | 502 (392,642) | 5·5 (4·3,6·8) | 742 (577,944) | 6·2 (5·1,7·5) | 12·1 (-3·9,31·9) |
| **Zimbabwe** | 46 (28,69) | 1·5 (1,2·1) | 84 (52,123) | 1·6 (1·1,2·2) | 7·1 (1,13·9) |
| **Tropical Latin America** | 2581 (1985,3307) | 7·7 (6·3,9·5) | 4715 (3675,6054) | 9·5 (7·8,11·5) | 23·7 (17·3,31·4) |
| **Brazil** | 2534 (1950,3241) | 7·8 (6·3,9·6) | 4591 (3565,5898) | 9·6 (7·9,11·6) | 23·4 (17,31·4) |
| **Paraguay** | 47 (34,64) | 5·1 (3·9,6·8) | 125 (88,169) | 7·3 (5·8,9·4) | 42·3 (20,73·4) |
| **Western Europe** | 37087 (29297,45959) | 39·1 (33·9,44·8) | 39336 (30393,49273) | 45·3 (38·2,52·1) | 15·9 (10·5,20·4) |
| **Andorra** | 6 (4,8) | 37·3 (27·8,52·2) | 8 (5,10) | 40·6 (29·4,54·7) | 8·6 (-27·1,59·9) |
| **Austria** | 672 (515,860) | 35·9 (31,42·1) | 790 (581,1016) | 43·4 (36,50·6) | 20·8 (7·7,34·5) |
| **Belgium** | 942 (733,1183) | 38·9 (33·7,44·4) | 1057 (794,1387) | 45·2 (37·7,52·9) | 16·2 (4·2,28·8) |
| **Cyprus** | 36 (24,54) | 21 (13·6,30·3) | 88 (62,121) | 25·2 (19·7,32·2) | 20·2 (-16,76) |
| **Denmark** | 747 (578,941) | 69·8 (62·1,79·4) | 676 (501,886) | 65·8 (56·6,76·1) | -5·7 (-16·8,5·3) |
| **Finland** | 530 (416,656) | 40·3 (35·1,45·9) | 491 (379,627) | 44·1 (37·6,50·7) | 9·3 (-1,20) |
| **France** | 5336 (3992,6869) | 33·4 (28·3,39·3) | 5871 (4236,7600) | 40·4 (33·2,47·8) | 20·8 (10·3,32·6) |
| **Germany** | 8934 (6966,11412) | 43·8 (38,51) | 7304 (5439,9702) | 47·2 (40·3,54·9) | 7·7 (-2·1,18·3) |
| **Greece** | 444 (365,549) | 17·8 (15·5,20·3) | 503 (394,622) | 29·2 (25·5,33·1) | 64·3 (48·3,81·9) |
| **Iceland** | 33 (25,40) | 49·5 (41·6,56·4) | 45 (35,58) | 54·7 (45·5,64·9) | 10·5 (-2·9,23·4) |
| **Ireland** | 482 (360,636) | 58·2 (49·1,67) | 658 (476,856) | 58·8 (47·8,70) | 1 (-10·4,12·2) |
| **Israel** | 186 (136,246) | 15·2 (12·6,18·3) | 368 (261,495) | 17·1 (14,20·5) | 12·1 (1·3,25·2) |
| **Italy** | 4817 (3729,6101) | 29·6 (24·6,35·4) | 4534 (3343,5916) | 36·8 (30,44·6) | 24·2 (17·4,30·5) |
| **Luxembourg** | 45 (35,58) | 44·4 (37·7,51·7) | 65 (46,86) | 42·6 (35·6,50·1) | -4 (-14·6,7·2) |
| **Malta** | 11 (9,14) | 13·6 (11·9,15·7) | 15 (11,19) | 17·2 (14·7,20·3) | 26·6 (13·2,41·6) |
| **Monaco** | 1 (1,2) | 18·1 (13·7,23·5) | 2 (1,3) | 24·9 (18·3,33) | 37·1 (5·6,81·4) |
| **Netherlands** | 1762 (1320,2249) | 47·1 (40·7,54) | 1694 (1243,2144) | 50·9 (42·8,58·8) | 8·1 (-1·3,18·9) |
| **Norway** | 456 (358,578) | 55·7 (49·1,63) | 615 (444,799) | 62·9 (52·3,74·2) | 13 (4·4,21·5) |
| **Portugal** | 482 (377,606) | 18·4 (15·6,21·6) | 353 (278,433) | 18·4 (15·8,21·3) | -0·1 (-10·6,11·4) |
| **San Marino** | 1 (1,1) | 9·8 (6·4,13·9) | 1 (1,1) | 10·7 (7·2,15·2) | 9·7 (-6·1,30·6) |
| **Spain** | 2509 (1871,3177) | 22·3 (18·5,26·7) | 2859 (2013,3869) | 28·5 (22·8,34·4) | 27·6 (13·4,43·3) |
| **Sweden** | 1066 (788,1367) | 53·6 (44·1,64·8) | 1391 (980,1840) | 63·5 (51,77·8) | 18·5 (8·4,27) |
| **Switzerland** | 931 (740,1154) | 56 (48·5,64·7) | 911 (670,1193) | 49·7 (41·8,57·8) | -11·2 (-22·1,-1) |
| **United Kingdom** | 6629 (5456,7981) | 57·1 (51·2,63·5) | 9004 (7203,10947) | 71·3 (61·8,80·8) | 24·9 (19·6,29·2) |
| **Western Sub-Saharan Africa** | 6087 (3592,8747) | 4·2 (2·9,5·7) | 26395 (17195,37211) | 6·4 (4·7,8·5) | 52·5 (13·4,109) |
| **Benin** | 138 (52,314) | 4·1 (2·2,7·8) | 671 (250,1558) | 5·7 (3,11·4) | 39·6 (-36·8,213·7) |
| **Burkina Faso** | 254 (81,611) | 4·1 (2·1,8) | 1060 (353,2800) | 5·7 (2·7,12·3) | 37·7 (-37·9,217·9) |
| **Cabo Verde** | 16 (5,38) | 5·5 (2·6,10·5) | 28 (10,69) | 6·1 (3·1,12·4) | 11·8 (-49·5,140·8) |
| **Cameroon** | 390 (169,817) | 4·7 (2·6,8·5) | 1932 (688,4695) | 6·6 (3·1,14·2) | 40·9 (-46·5,275·5) |
| **Chad** | 133 (46,328) | 3·6 (2,6·9) | 656 (213,1616) | 5 (2·5,9·8) | 38·4 (-35·3,188·5) |
| **CÃ´te d'Ivoire** | 392 (149,931) | 3·9 (2·1,7·9) | 1463 (542,3736) | 5·9 (2·9,13·1) | 50·4 (-39·3,272·5) |
| **Gambia** | 32 (11,79) | 4·2 (2·2,8·2) | 180 (60,417) | 7·6 (3·4,15·6) | 83·2 (-28·9,345) |
| **Ghana** | 827 (345,1772) | 7·3 (4·1,13·1) | 2857 (1169,6468) | 11·2 (6·8,20·3) | 54·3 (-27·2,237·2) |
| **Guinea** | 146 (57,338) | 3·8 (2·1,7·1) | 706 (264,1691) | 6·2 (3·1,12·8) | 62·2 (-26·5,250·4) |
| **Guinea-Bissau** | 41 (16,92) | 5·2 (2·7,10·1) | 141 (58,331) | 7·4 (3·8,15) | 44 (-38·9,216·1) |
| **Liberia** | 72 (29,164) | 3·9 (2·2,7·6) | 327 (118,774) | 6·4 (2·9,13·6) | 64·3 (-32·4,257·2) |
| **Mali** | 312 (101,749) | 5·1 (2·5,10·3) | 1478 (456,3402) | 7·1 (3·3,14) | 38·4 (-38·6,188·2) |
| **Mauritania** | 88 (39,192) | 5·7 (3·4,10·2) | 311 (121,706) | 8·2 (4·2,16·2) | 44 (-38·8,222·5) |
| **Niger** | 203 (59,517) | 4 (2,7·8) | 848 (215,2094) | 4·7 (2·3,9·4) | 19·6 (-43·3,159·8) |
| **Nigeria** | 2508 (1409,3942) | 3·6 (2·4,5·2) | 11740 (6803,19930) | 5·9 (3·9,9) | 62·9 (7·6,160·1) |
| **Sao Tome and Principe** | 1 (1,2) | 1·9 (1·2,2·9) | 4 (2,8) | 2·7 (1·7,4·4) | 41·3 (-19·6,150·1) |
| **Senegal** | 288 (94,727) | 4·9 (2·5,10·2) | 1028 (372,2606) | 7·1 (3·6,15·4) | 42·6 (-47,240) |
| **Sierra Leone** | 103 (39,275) | 3·4 (1·9,7) | 481 (173,1129) | 5·6 (2·8,11·3) | 66·2 (-36·1,286·6) |
| **Togo** | 144 (61,309) | 4·8 (2·7,8·9) | 484 (184,1060) | 6·5 (3·2,12·9) | 34·9 (-46·1,193·4) |

**Figure S1**

**
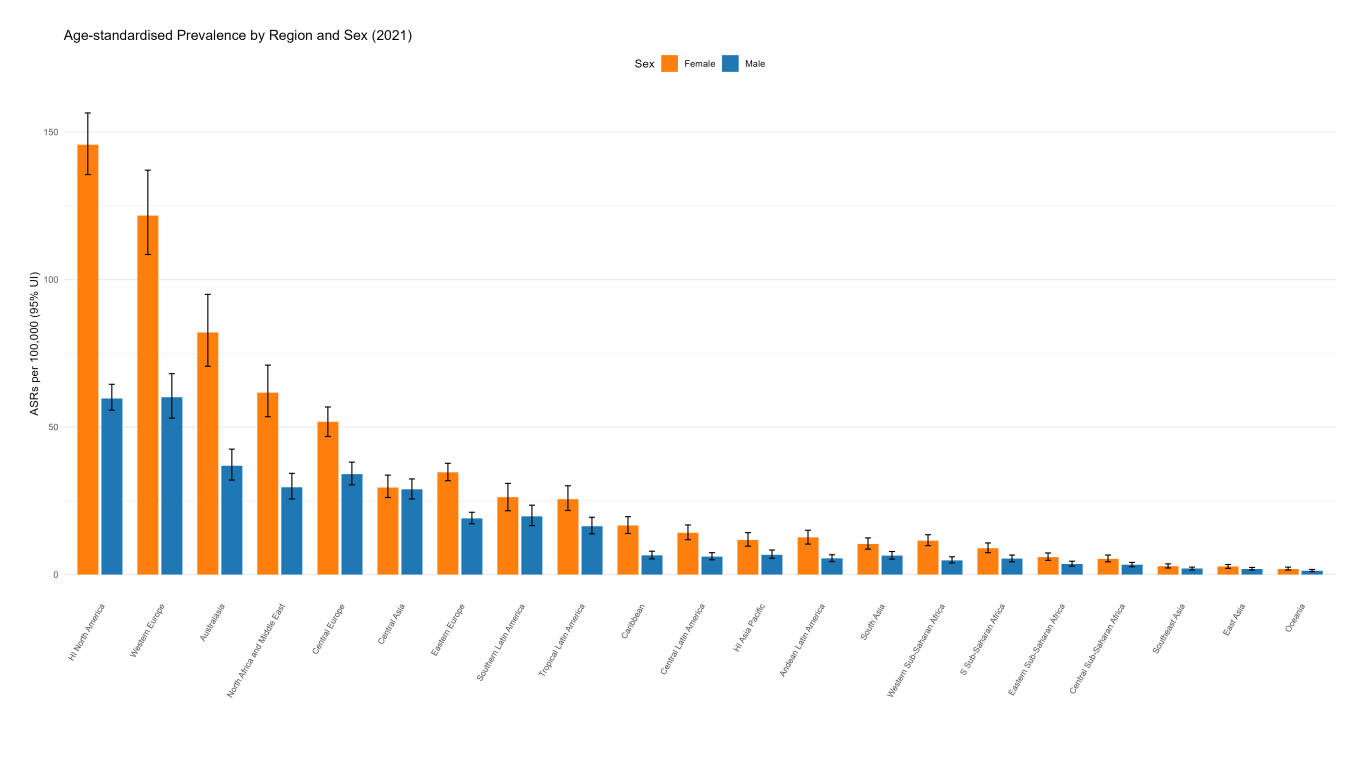
**

**Figure S2**

**
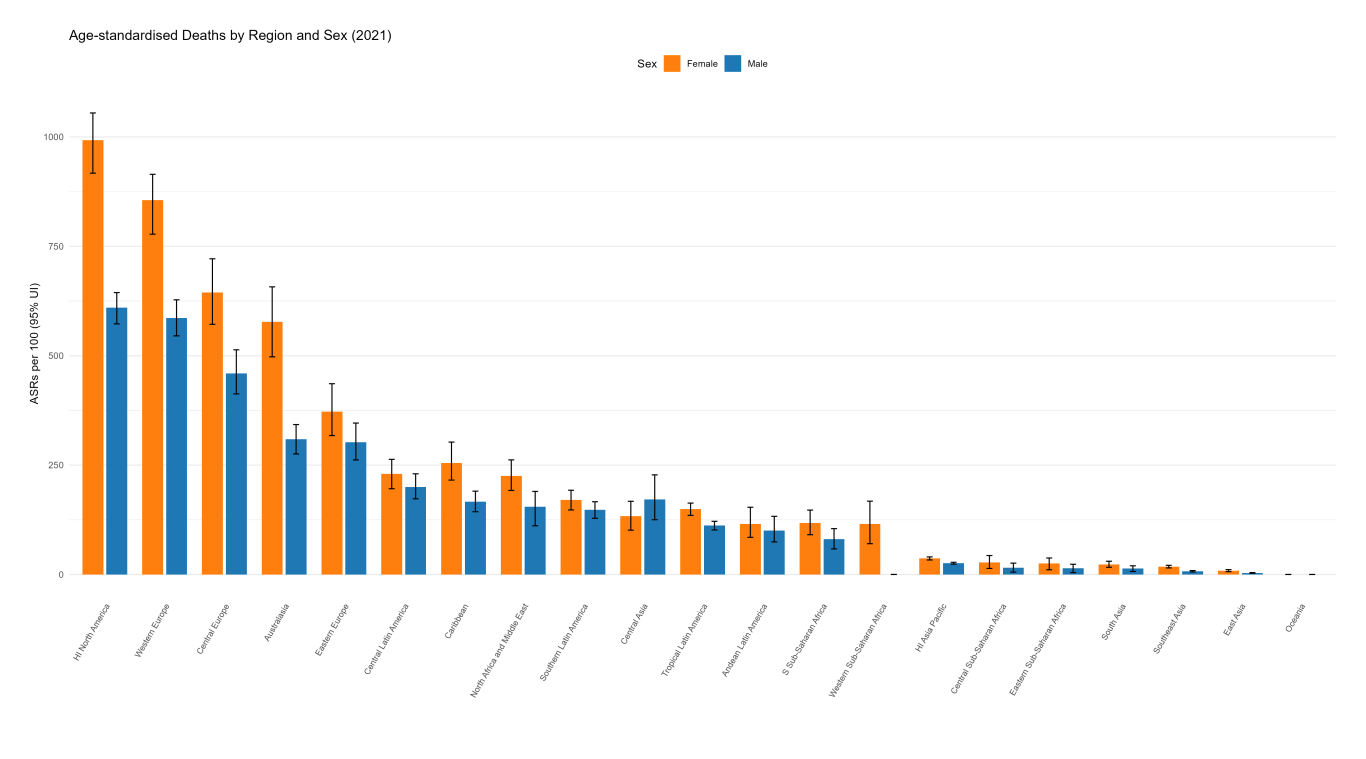
**

**Figure S3**

**
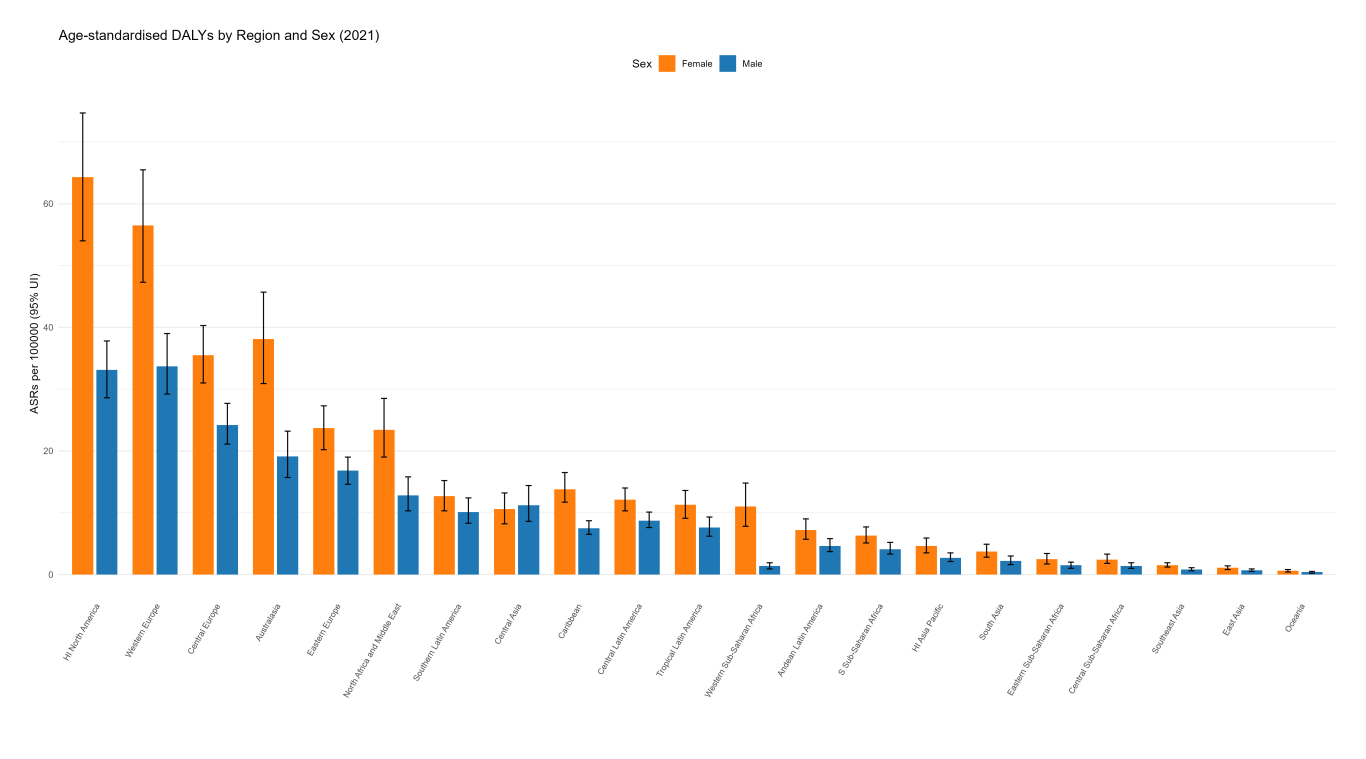
**

**Figure S4**

**
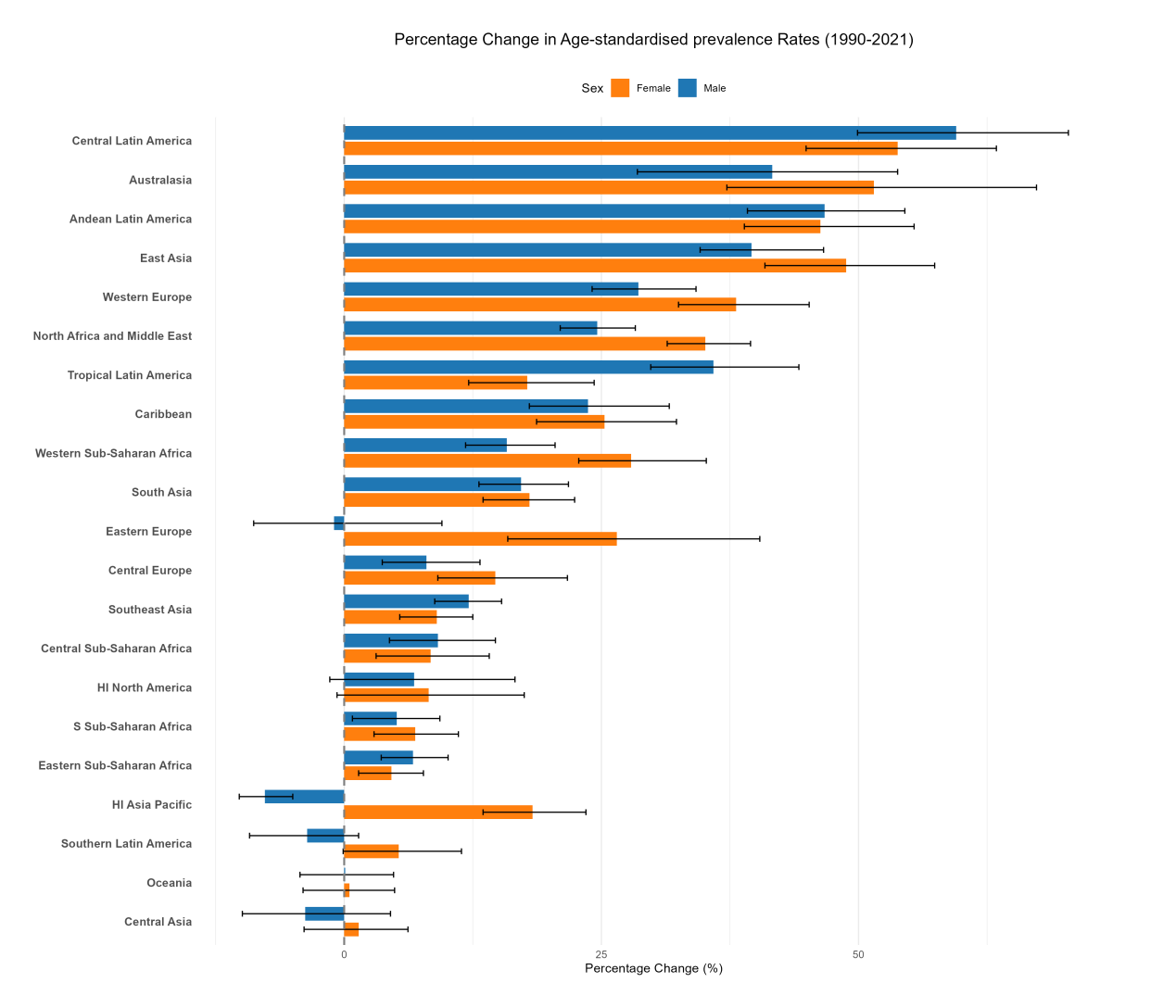
**

**Figure S5**

**
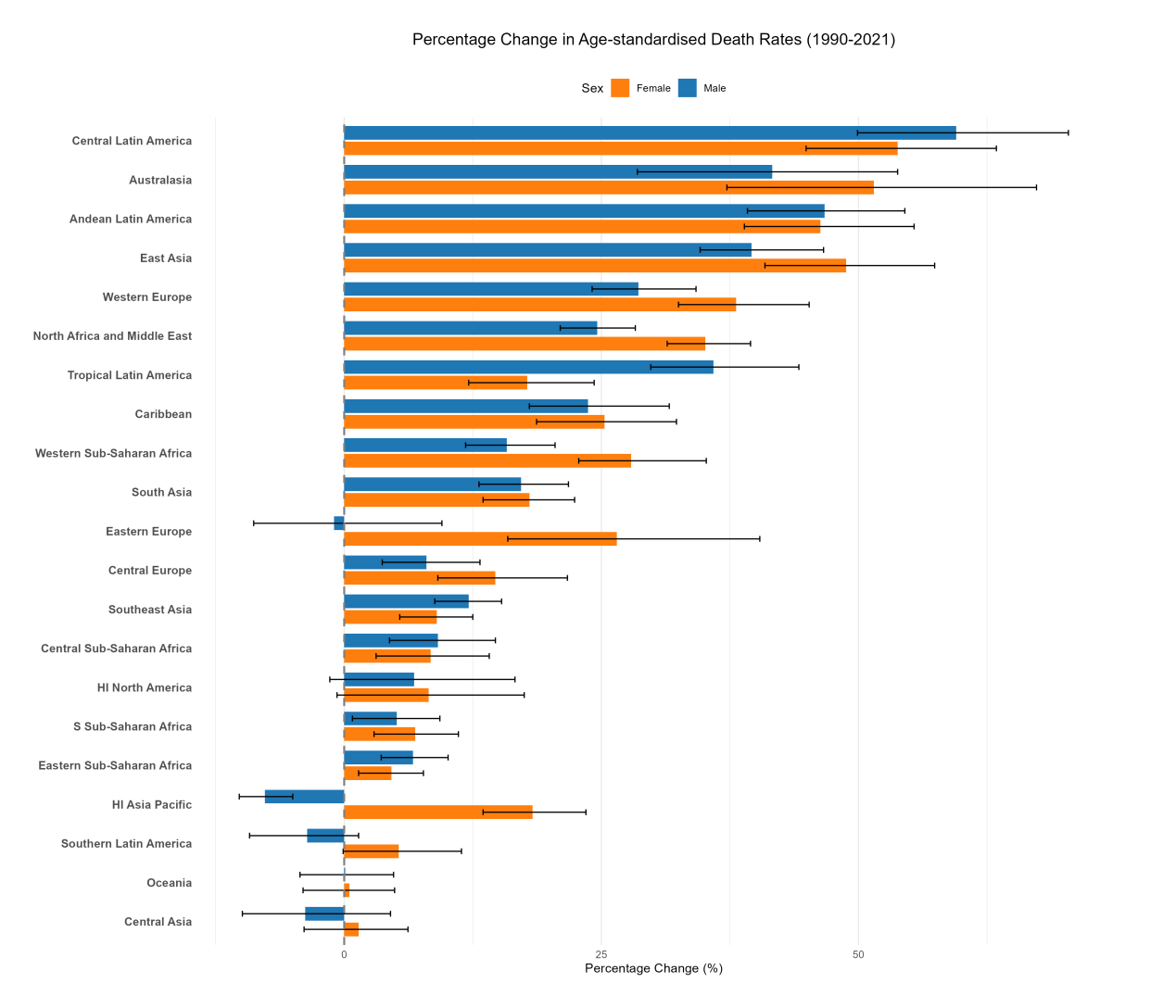
**

**Figure S6**

**
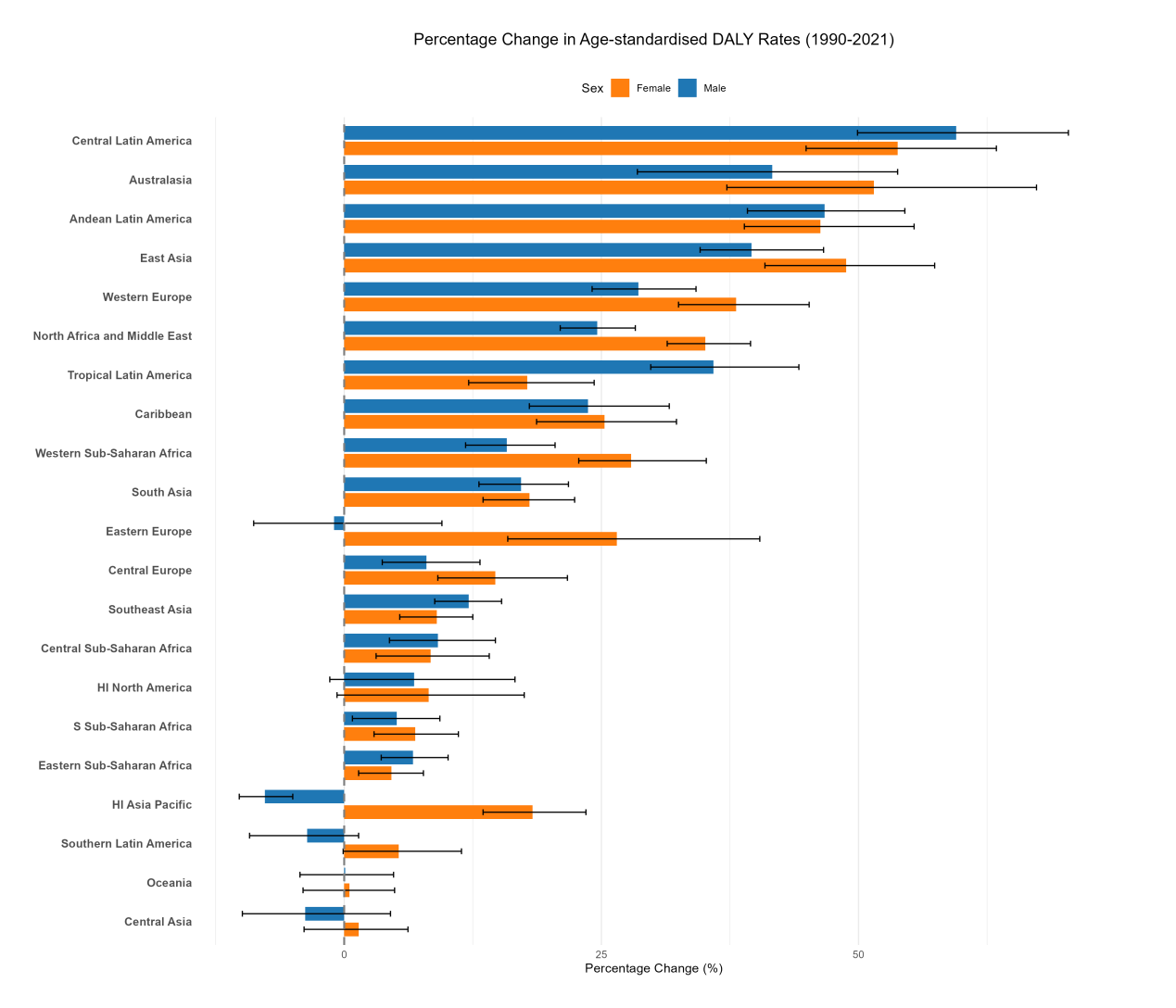
**

**Figure S7**

**
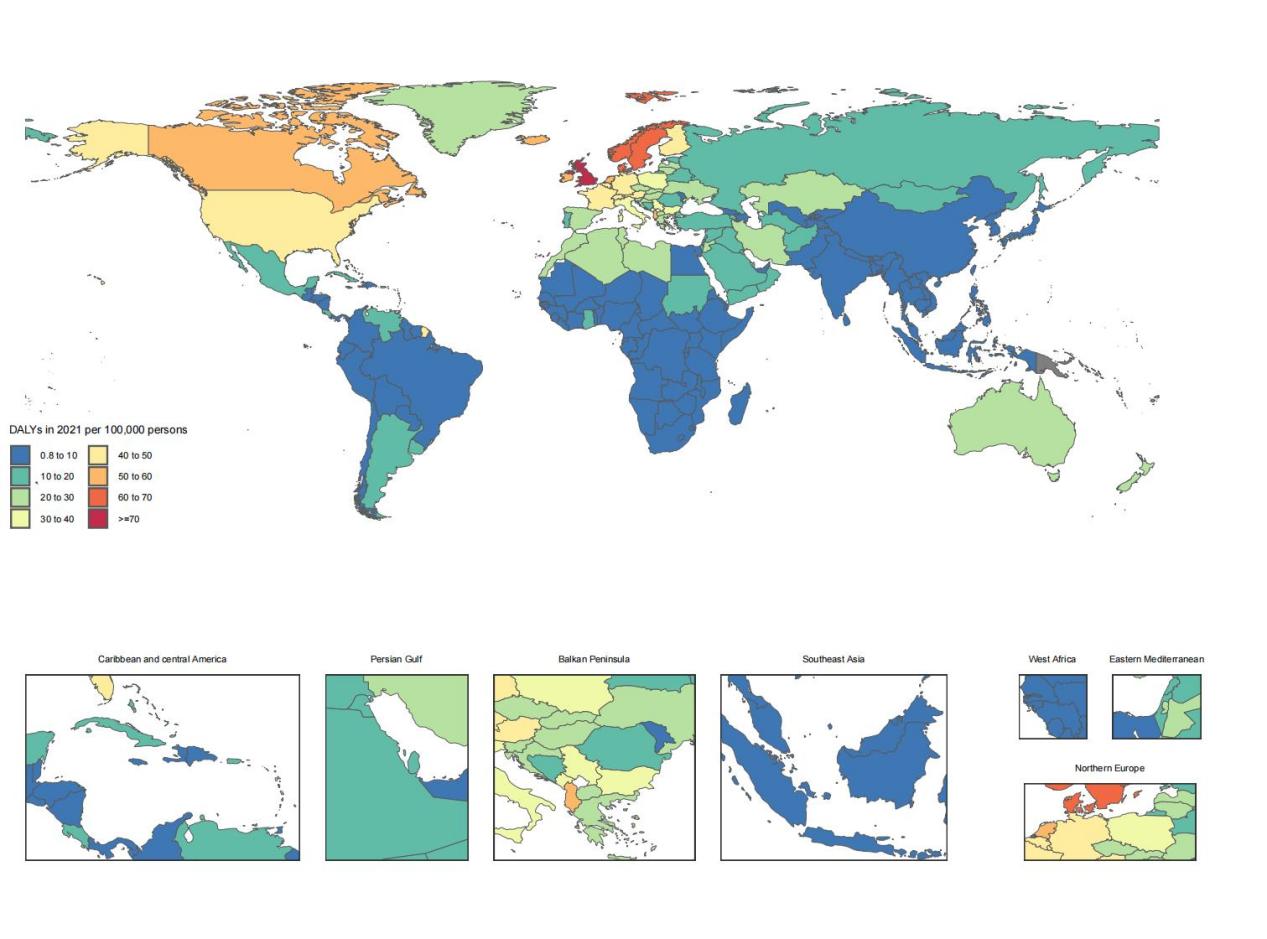
**

**Figure S8**

**
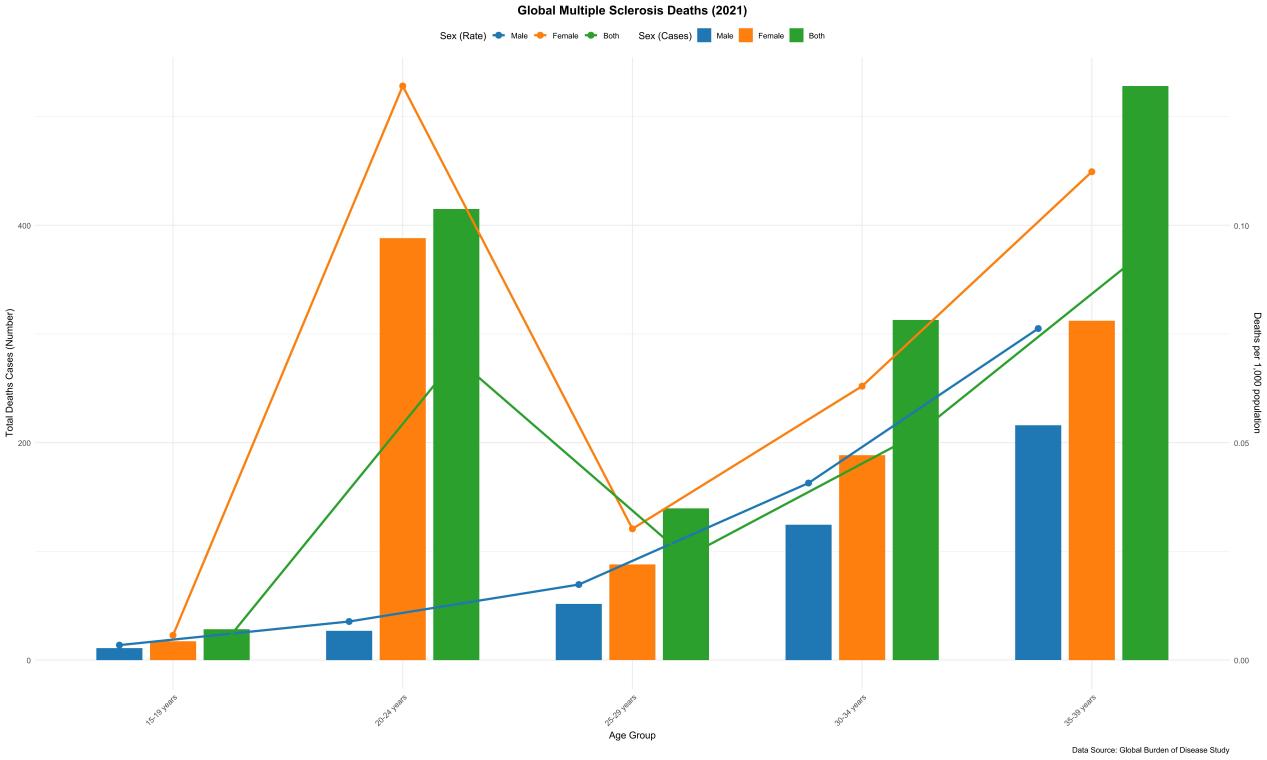
**

**Figure S9**

**
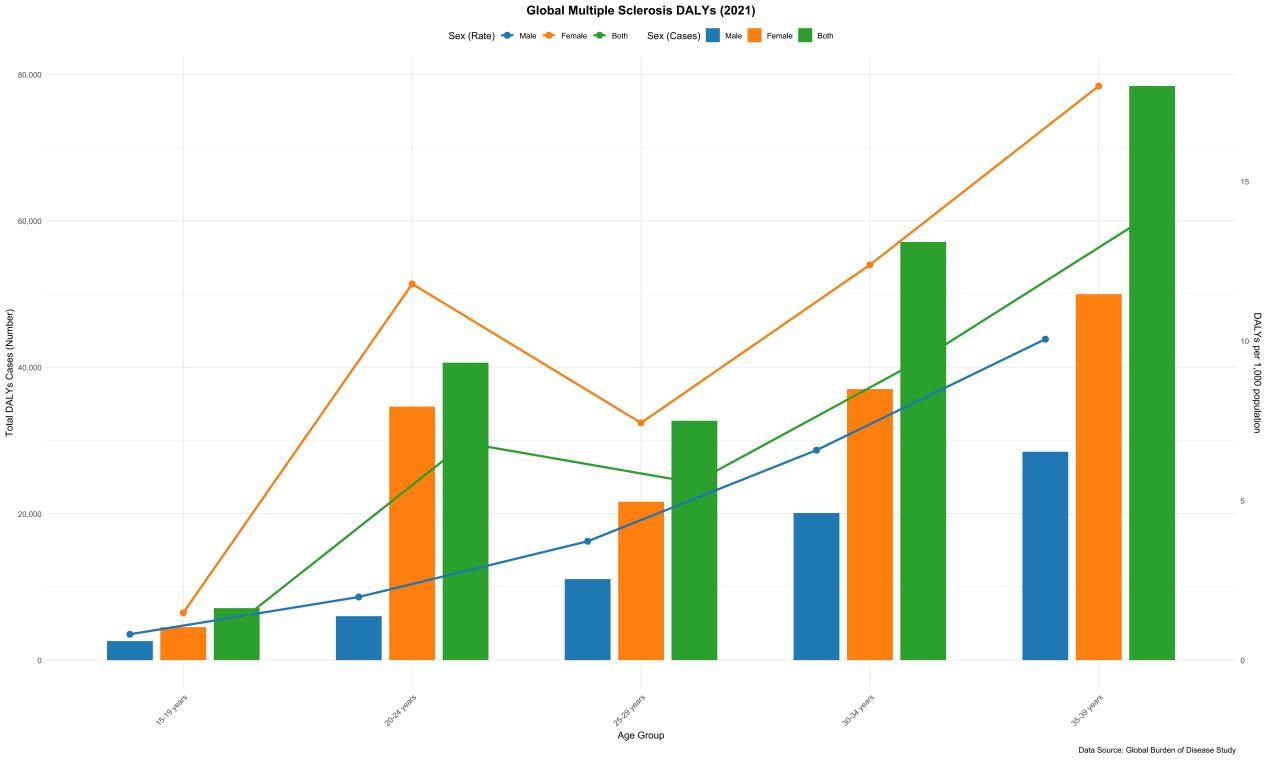
**

**Figure S10**

**
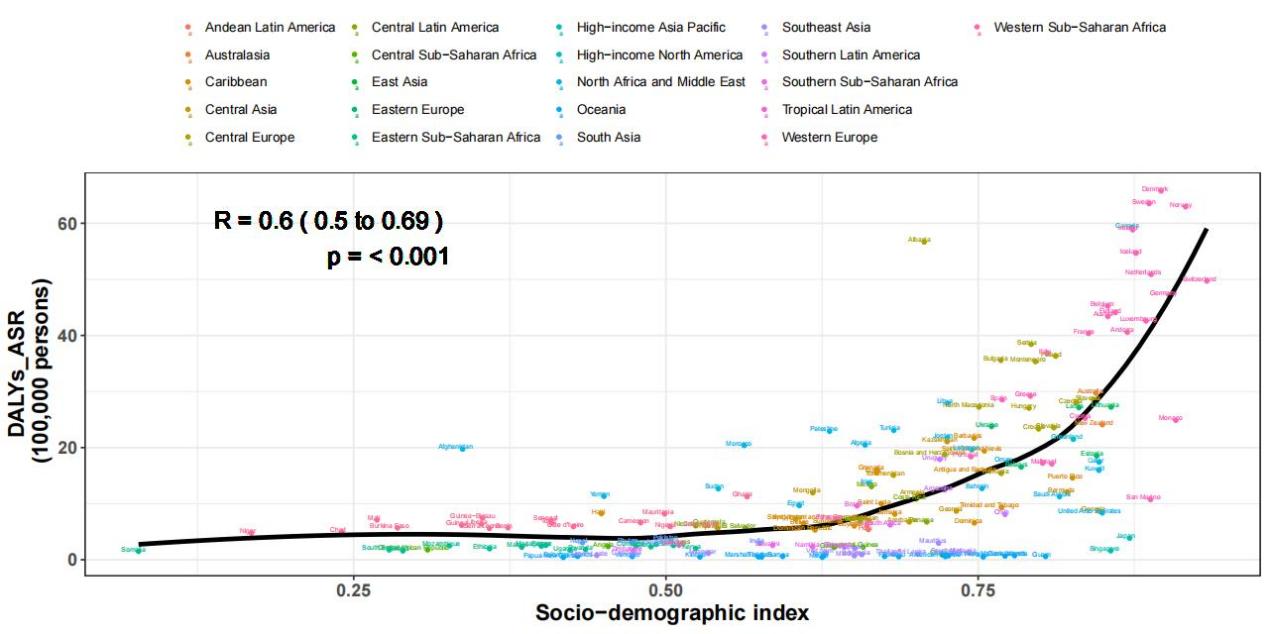
**

**Figure S11**

**
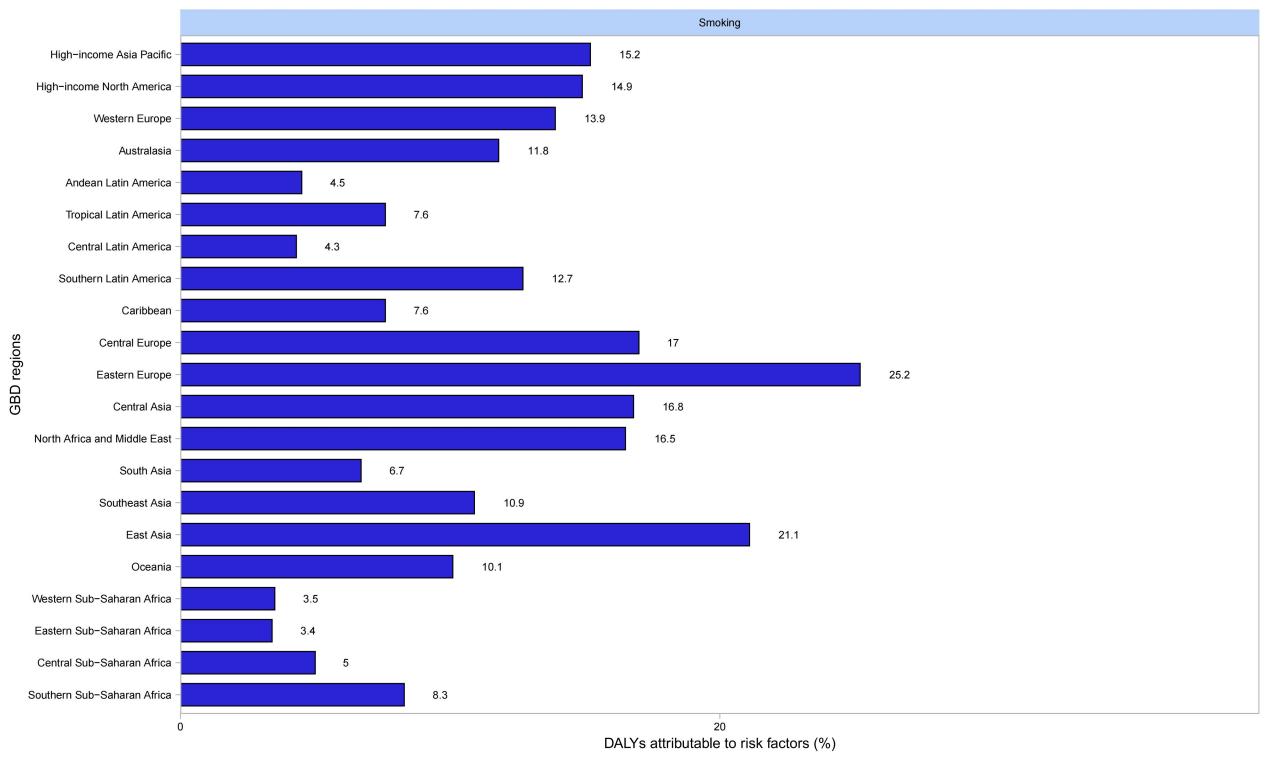
**

**Figure S12**

**
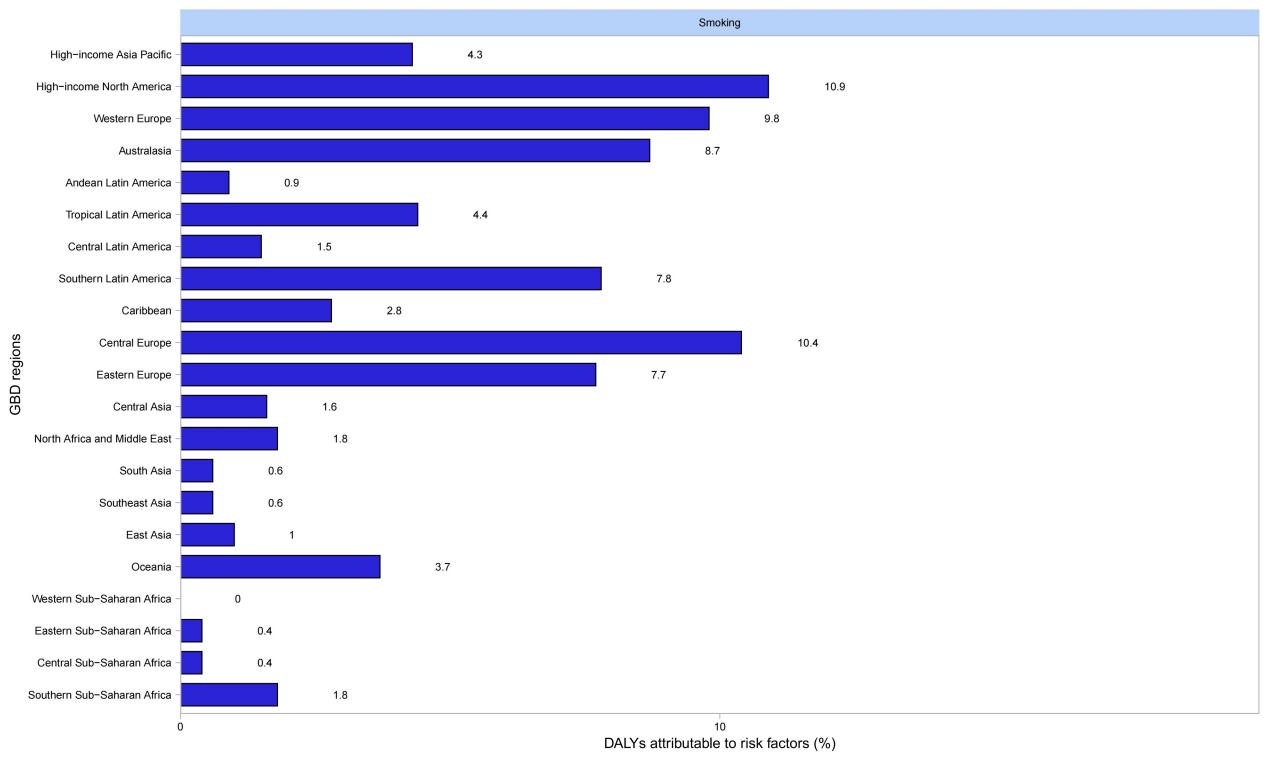
**
